# Four New Triterpenoids from *Callicarpa kwangtungensis*

**DOI:** 10.3390/molecules20059071

**Published:** 2015-05-19

**Authors:** Guo-Ping Zhou, Yan Yu, Ming-Ming Yuan, Tengfei Ji, Hui-Zheng Fu, Rui-Jian Zhong

**Affiliations:** 1 Jiangxi Provincial Institute for Drug and Food Control, Nanchang 330029, China; E-Mails: 13870868633@139.com (G.-P.Z.); 15070029890@163.com (Y.Y.); 13426688499@163.com (M.-M.Y.); fhzfhz620@sohu.com (H.-Z.F.); 2 State Key Laboratory of Bioactive Substance and Function of Natural Medicines, Institute of Materia Medica, Chinese Academy of Medical Sciences & Peking Union Medical College, Beijing 100050, China; 3 Pharmaceutical Department of Nanchang University, Nanchang 330006, China

**Keywords:** *Callicarpa kwangtungensis*, Verbenaceae, triterpenoids, NMR, MS

## Abstract

Four new triterpenoids which were identifed as 2α,3β,6β,19α-tetrahydroxy- oleanolic acid 28-*O*-β-d-glucopyranoside (**1**), 2-*O*-β-d-glucopyranosyloxy-3α,19α-di-hydroxyoleanolic acid (**2**), 2-*O*-β-d-glucopyranosyloxy-3α,19α-dihydroxyursolic acid (**3**), 2α,3α,6β,19α-tetrahydroxyursolic acid 28-*O*-β-d-glucopyranoside (**4**), were isolated from the aerial parts of *Callicarpa kwangtungensis* together with three known triterpenoids identified as 2α,3β,21β-trihydroxyursolic acid 28-*O*-β-d-glucopyranoside (**5**), 2α,3α,19α,23-tetrahydroxyoleanolic acid 28-*O*-β-d-glucopyranoside (**6**), 2α,3α,19α,23-tetrahydroxyursolic acid 28-*O*-β-d-glucopyranoside (**7**). Their structures were elucidated by the combination of mass spectrometry (MS), one and two-dimensional NMR experiments.

## 1. Introduction

*Callicarpa kwangtungensis* Chun, belonging to the family Verbenaceae, is distributed widely in the Guangdong, Guangxi, and Jiangxi provinces of China [1]. The aerial parts of *C. kwangtungensis* are used in Chinese herbal medicine for the treatment of bleeding wounds and hematemesis [1]. Previous phytochemical studies of the genus *Callicarpa* led to the isolation of flavonoids, triterpenoids, and phenylpropanoid glycosides. Some sesquiterpenoids such as callicarpenal, intermedeol, α-humulane were isolated from *Callicarpa americana*, *C. japonica* and *C. pedunculata*; They also include a few diterpenoids such as 16,17-dihydroxy-3-oxophyllocladane, 16-hydroxy-17-acetoxy-3-oxophyllocladane, isopropylidenocalliterpenone, and calliphyllin from *C. acuminata*, *C. formosana*, *C. macrophylla*, *C. maingayi* and *C. pentandra*; and many triterpenoids such as 2a,3a,24-trihydroxyoleanolic acid, ursolic acid and β-amyrin from *C. formosana*; oleanolic acid, betulin and α-amyrin from *C. macrophylla*; maslinic acid, bauerenol, 2α,3β-dihydroxyursolic acid from *C. bodinieri*; several phenylpropanoid glycosides from *C. pentandra*, *C. kwangtungensis* and *C. furfuraceae* [2,3,4]. In our previous investigations, a novel phenylpropanoid glycoside was isolated from the aerial parts of *C. kwangtungensis* [5,6].

**Figure 1:**
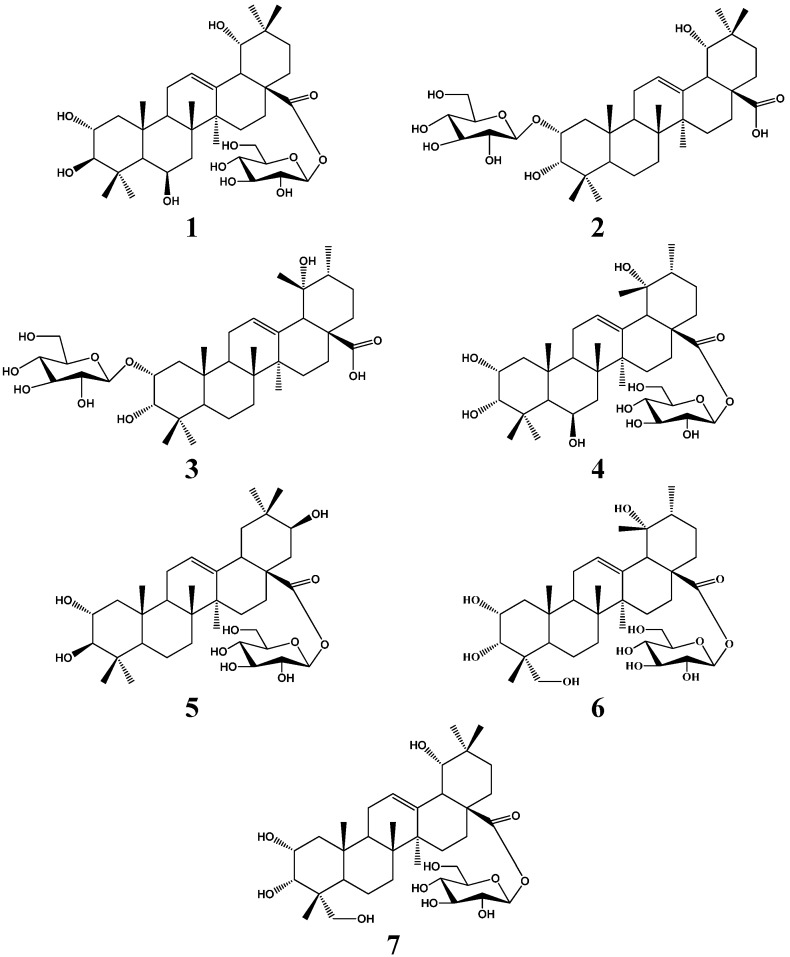
Structures of compounds **1**–**7**.

As part of a study of the chemical constituents of *C. kwangtungensis*, a 95% EtOH extract of the aerial parts of *C. kwangtungensis* has now been investigated. Four new triterpenoids have been isolated and identified as 2α,3β,6β,19α-tetrahydroxyoleanolic acid 28-*O*-β-d-glucopyranoside (**1**), 2-*O*-β-d-glucopyranosyloxy-3α,19α-dihydroxyoleanolic acid (**2**), 2-*O*-β-d-glucopyranosyloxy-3α,19α-dihydroxyursolic acid (**3**), 2α,3α,6β,19α-tetrahydroxyursolic acid 28-*O*-β-d-glucopyranoside (**4**), In addition three known triterpenoids identified as 2α,3β,21β-trihydroxyursolic acid 28-*O*-β-d-glucopyranoside (**5**), 2α,3α,19α,23-tetrahydroxyoleanolic acid 28-*O*-β-d-glucopyranoside (**6**) and 2α,3α,19α,23-tetrahydroxyursolic acid 28-*O*-β-d-glucopyranoside (**7**) have also been isolated (Figure 1). We report herein the isolation and structure elucidation of these compounds.

## 2. Results and Discussion

The EtOH extract of the aerial parts of *Callicarpa kwangtungensis* was successively partitioned with petroleum ether, EtOAc, and *n*-BuOH. The EtOAc-soluble portion was separated by a combination of silica gel, ODS column chromatography, and preparative HPLC to afford four new triterpenoids: 2α,3β,6β,19α-tetrahydroxyoleanolic acid 28-*O*-β-d-glucopyranoside (**1**), 2-*O*-β-d-glucopyranosyloxy-3α,19α-dihydroxyoleanolic acid (**2**), 2-*O*-β-d-glucopyranosyloxy-3α,19α-dihydroxyursolic acid (**3**), 2α,3α,6β,19α-tetrahydroxyursolic acid 28-*O*-β-d-glucopyranoside (**4**), together with three known triterpenoids 2α,3β,21β-trihydroxyursolic acid 28-*O*-β-d-glucopyranoside (**5**), 2α,3α,19α,23-tetrahydroxyoleanolic acid 28-*O*-β-d-glucopyranoside (**6**) and 2α,3α,19α,23-tetra-hydroxyursolic acid 28-*O*-β-d-glucopyranoside (**7**). Their structures were elucidated by extensive NMR techniques mainly including 1D NMR (^1^H- and ^13^C-NMR), 2D NMR (COSY, NOESY, HSQC, and HMBC), and ESI-MS.

**Figure 2:**
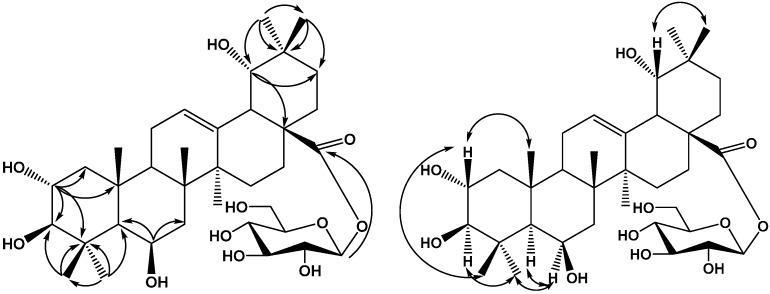
Key HMBC and NOESY correlations of compound **1**.

Compound **1** was obtained as a white amorphous powder, which gave a positive result in the Liebermann-Burchard test. Acid hydrolysis of compound **1** with 2 mol/L HCl/1,4-dioxane (1:1, *v/v*) furnished glucose, identified by TLC by comparison with an authentic sample. The positive optical rotation (${\left[\alpha \right]}_{D}^{20}$ +45.1, *c* 0.03, H_2_O) indicated the d-configuration of glucose, The sugar identity was further confirmed by the chemical shifts and coupling constants in the ^1^H- and ^13^C-NMR spectra. The HR-ESI-MS of 1 showed a quasi-molecular ion peak at *m*/*z* 665.3901 [M−H]^−^, indicating a molecular formula of C_36_H_58_O_10_ (calcd. for C_36_H_57_O_10_, 665.3909, Δamu 2.6 ppm). The ^1^H- and ^13^C-NMR spectra of **1** in pyridine-d_5_ showed typical signals for an oleanane pentacyclic triterpenoid skeleton including seven tertiary methyl groups [δ_H_ 0.99,1.17, 1.49, 1.66, 1.79, 1.79, 1.83, (each 3H, s)], as well as one olefinic proton at δ_H_ 5.61 (1H, br s), two olefinic carbons (δ_C_ 124.5 and 144.2) and an ester carbonyl at δ_C_ 177.17. The ^1^H-NMR spectra of **1** exhibited four oxymethine protons at δ_H_ 4.87 (1H, s), 4.30 (1H, m), 3.63 (1H, s), and 3.43 (1H, d, *J* = 9.0 Hz). The data thus suggested that **1** is an oleanane-type triterpene with four hydroxy groups, a trisubstituted double bond, and a carboxyl. Comparison of the NMR spectroscopic data of **1** with those of arjunetin [7] demonstrated that the two compounds were almost identical, except for an additional hydroxyl group at C-6 (δ_C_ 68.3). These data suggested that **1** is a 6-oxygenated derivative of arjunetin, which was further confirmed by HMBC and NOESY experiments on **1**. The existence of four hydroxy groups at C-2, C-3, C-6 and C-19 was supported by the HMBC spectrum. HMBC correlations (Figure 2) were observed between H-1 (δ_H_ 2.35 and δ_H_ 1.40) and C-25 (δ_C_ 18.9), C-4 (δ_C_ 40.2), C-2 (δ_C_ 69.3), C-3 (δ_C_ 84.4); between H-2 (δ_H_ 4.30) and C-3 (δ_C_ 84.4); H-3 (δ_H_ 3.43) and C-2 (δ_C_ 69.3), C-4 (δ_C_ 40.2), C-24 (δ_C_ 19.0); between H-19 (δ_H_ 3.63) and C-21 (δ_C_ 29.5), C-17 (δ_C_ 47.0); between H-5 (δ_H_ 1.21), H-7 (δ_H_ 2.00) and C-6 (δ_C_ 68.3). The orientations of the hydroxyls at C-2, C-3, C-6 and C-19 were determined using NOESY correlations. The NOESY correlation of H-3 (δ_H_ 3.43) with H-23 (δ_H_ 1.43) indicated that the hydroxyl at C-3 should be β-oriented; the NOESY correlations of H-2 (δ_H_ 4.30) with H-24 (δ_H_ 1.79) and H-25 (δ_H_ 1.79) implied that 2-OH group had an α-orientation; the NOESY correlations of H-6 (δ_H_ 4.87) with H-5 (δ_H_ 1.21) and H-23 (δ_H_ 1.49) implied that the 6-OH group had a β-orientation; the NOESY correlations of H-19 (δ_H_ 3.63) with H-30 (δ_H_ 1.66) implied that the 19-OH group had an α-orientation; Therefore, the aglycon moiety of **1** was identified as 2α,3β,6β,19α-tetrahydroxyoleanolic acid. In the ^1^H-NMR spectrum of **1**, the relatively large ^3^*J*_H-1,H-2_ coupling constant of the anomeric proton at δ_H_ 6.36 of the d-glucopyranosyl moiety (*J* = 7.8 Hz) indicated a β-configuration for d-glucose. HMBC correlations between the anomeric proton at δ_H_ 6.36 (1H, d, *J* = 7.8 Hz) and the carbon signal at C-28 (δ_C_ 177.7) indicated that a β-d-glucopyranosyl moiety was attached to the C-28 position of the aglycone. On the basis of the foregoing evidence, the structure of **1** was determined as 2α,3β,6β,19α-tetrahydroxyoleanolic acid 28-*O*-β-d-glucopyranoside.

**Figure 3:**
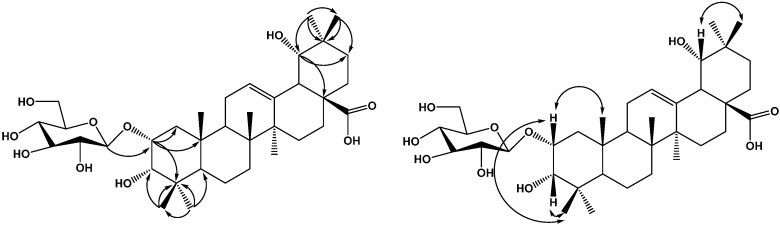
Key HMBC and NOESY correlations of compound **2**.

Compound **2** was obtained as a white amorphous powder, which gave a positive result in the Liebermann-Burchard test. Acid hydrolysis of compound **2** with 2 mol/L HCl/1,4-dioxane (1:1, *v/v*) furnished glucose, identified by TLC comparison with an authentic sample. The positive optical rotation (${\left[\alpha \right]}_{D}^{20}$ +46.2, c 0.03, H_2_O) indicated the d-configuration of glucose. In the (−) and (+)-ESI-MS of **2**, quasimolecular ion peaks were observed at *m*/*z*: 649 [M−H]^−^ and 673 [M+Na]^+^, respectively. The HR-ESI-MS (*m*/*z* 649.3962 [M−H]^−^) analysis revealed the molecular formula of **2** to be C_36_H_58_O_10_ (calcd. for C_36_H_57_O_10_, 649.3968, Δamu 0.9 ppm). The ^1^H- and ^13^C-NMR spectra of **2** in pyridine-*d_5_* showed typical signals for an oleanane pentacyclic triterpenoid skeleton including seven tertiary methyl groups [δ_H_ 0.88, 0.97, 1.06, 1.11, 1.19, 1.23, 1.54, (each 3H, s)], as well as one olefinic proton at δ_H_ 5.57 (1H, br s), a pair of olefinic carbons at δ_C_ 124.3 and 145.3, typical for a double bond at C-12 (13) in an oleanane pentacyclic triterpenoid skeleton [6,7] and a carboxyl carbon at δ_C_ 181.3. The ^1^H-NMR spectra of **2** exhibited three oxymethine protons at δ_H_ 4.46 (1H, m), 4.05 (1H, d, *J* = 2.4 Hz), 3.63 (1H, s), The data thus suggested that the aglycon moiety of **2** is an oleanane-type triterpene with three hydroxy groups, a trisubstituted double bond, and a carboxyl. Comparison of the NMR spectroscopic data of **2** with those of 2α,3α,19α-dihydroxyoleanolic acid 28-*O*-β-d-glucopyranoside [7] demonstrated that the aglycon moiety of the two compounds were almost identical. These data suggesting that **2** has the same aglycon moiety as 2α,3α,19α-dihydroxyoleanolic acid 28-*O*-β-d-glucopyranoside were further confirmed by HMBC and NOESY experiments on **2**. The existence of three hydroxy groups at C-2, C-3 and C-19 was supported by the HMBC spectrum, HMBC correlations (Figure 3) were observed between H-1 (δ_H_ 2.00 and δ_H_ 1.86) and C-25 (δ_C_ 16.9), C-4 (δ_C_ 39.2), C-2 (δ_C_ 76.9); between H-2 (δ_H_ 4.46) and C-3 (δ_C_ 79.0); between H-3 (δ_H_ 4.05) and C-2 (δ_C_ 76.9), C-5 (δ_C_ 49.5); between H-19 (δ_H_ 3.63) and C-21 (δ_C_ 29.6), C-17 (δ_C_ 44.6). The configuration of the hydroxyls at C-2, C-3 and C-19 were determined using NOESY correlations. The NOESY correlation of H-2 (δ_H_ 4.46) with H-24 (δ_H_ 0.88) and H-25 (δ_H_ 0.97) indicated that the hydroxyl at C-2 should be in an α-orientation; the NOESY correlations of H-3 (δ_H_ 4.05) with H-24 (δ_H_ 0.88) implied that 3-OH group had an α-orientation; the NOESY correlations of H-19 (δ_H_ 3.63) with H-30 (δ_H_ 1.54) implied that 19-OH group had an α-orientation. Therefore, the aglycon moiety of **2** was identified as 2α,3α,19α-trihydroxyoleanolic acid. In the ^1^H-NMR spectrum of **2**, the relatively large ^3^*J*_H-1,H-2_ coupling constant of the anomeric proton at δ_H_ 5.16 of the d-glucopyranosyl moiety (*J* = 7.8 Hz) indicated a β-configuration for d-Glc. HMBC correlations between the anomeric proton at δ_H_ 6.36 (1H, d, *J* = 7.8 Hz) and the carbon signal at C-2 (δ_C_ 76.9) indicated that a β-d-glucopyranosyl moiety was attached to the C-2 position of the aglycone. On the basis of the foregoing evidence, the structure of **2** was determined as 2-*O*-β-d-glucopyranosyloxy-3α,19α-dihydroxyoleanolic acid.

**Figure 4:**
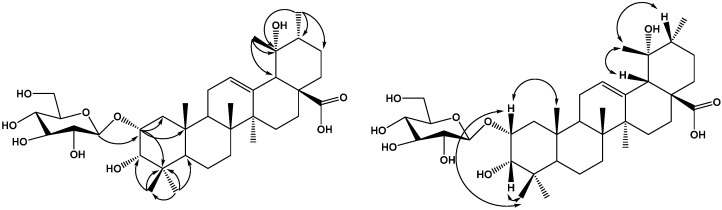
Key HMBC and NOESY correlations of compound **3**.

Compound **3** was obtained as a white amorphous powder, which gave a positive result in the Liebermann-Burchard test. Acid hydrolysis of compound **3** with 2 mol/L HCl/1,4-dioxane (1:1, *v/v*) furnished glucose, identified by TLC comparison with an authentic sample. The positive optical rotation (${\left[\alpha \right]}_{D}^{20}$ +45.7, *c* 0.03, H_2_O) indicated the d-configuration of glucose. In the (−) and (+)-ESI-MS of **3**, quasimolecular ion peaks observed at *m*/*z*: 649 [M−H]^−^ and 673 [M+Na]^+^ indicated the molecular weight of **3** is 650. The HR-ESI-MS of **3** showed a quasi-molecular ion peak at *m*/*z* 649.3952 [M−H]^−^, indicating a molecular formula of C_36_H_58_O_10_ (calcd. for C_36_H_57_O_10_, 649.3958, Δamu 0.41 ppm). The ^1^H and ^13^C-NMR spectra of **3** in pyridine-d_5_ showed typical signals for an ursane pentacyclic triterpenoid skeleton, including six tertiary methyl groups [δ_H_ 0.86, 0.96, 1.09, 1.22, 1.44, 1.62 (each 3H, s)] and one secondary methyl signal at δ_H_ 1.14 (3H, d, *J* = 6.6 Hz), as well as one olefinic proton at δ_H_ 5.56 (1H, br s), two olefinic carbons (δ_C_ 128.4 and 140.4) and a carboxyl carbon at δ_C_ 181.1. The ^1^H and ^13^C-NMR spectra of **3** exhibited two oxymethine protons at δ_H_ 4.46 (1H, m), 4.03 (1H, d, *J* = 2.4 Hz) and one hydroxy group attached to a tertiary carbon. The data thus suggested that **3** is an ursane-type triterpene with three hydroxy groups, a trisubstituted double bond, and a carboxyl. Comparison of the NMR spectroscopic data of **3** with those of 2α,3β,19α-trihydroxyurs-12-en-28-*O*-β-d-glucopyranoside. [7] demonstrated that the two compounds have the same aglycon moiety, only differing in the orientation of the hydroxy group at C-3. The existence of three hydroxy groups at C-2, C-3 and C-19 was supported by the HMBC spectrum, HMBC correlations (Figure 4) were observed between H-1 (δ_H_ 1.80 and δ_H_ 1.94) and C-25 (δ_C_ 17.0), C-4 (δ_C_ 39.0), C-2 (δ_C_ 76.6), C-3 (δ_C_ 79.1); between H-2 (δ_H_ 4.46) and C-3 (δ_C_ 79.1); H-3 (δ_H_ 4.03) and C-2 (δ_C_ 76.6), C-4 (δ_C_ 39.0), C-24 (δ_C_ 22.8); between H-18 (δ_H_ 3.05), H-30 (δ_H_ 1.14), H-29 (δ_H_ 1.44) and C-19 (δ_C_ 73.1). The configuration of the hydroxyls at C-2, C-3 and C-19 were determined using NOESY correlations. The NOESY correlation of H-3 (δ_H_ 4.03) with H-24 (δ_H_ 0.86) indicated that the hydroxyl at C-3 should be α-oriented; the NOESY correlations of H-2 (δ_H_ 4.46) with H-24 (δ_H_ 0.86) and H-25 (δ_H_ 0.96) implied that the 2-OH group had an α-orientation; the NOESY correlations of H-29 (δ_H_ 1.44) with H-18 (δ_H_ 3.05) and H-20 (δ_H_ 1.50) implied that 19-OH group had an α-orientation; Therefore, the aglycon moiety of **3** was identified as 2α,3α,19α-trihydroxyoursolic acid. In the ^1^H-NMR spectrum of **3**, the relatively large ^3^*J*_H-1,H-2_ coupling constant of the anomeric proton at δ_H_ 5.14 of d-glucopyranosyl moiety (*J* = 7.8 Hz) indicated a β-configuration for d-Glc. HMBC correlations between the anomeric proton at δ_H_ 5.14 (1H,d, *J* = 7.8 Hz) and the carbon signal at C-2 (δ_C_ 76.6) indicated that a β-d-glucopyranosyl moiety was attached to the C-2 position of the aglycone. On the basis of the foregoing evidence, the structure of **3** was determined as 2-*O*-β-d-glucopyranosyloxy-3α,19α-dihydroxyursolic acid.

**Figure 5:**
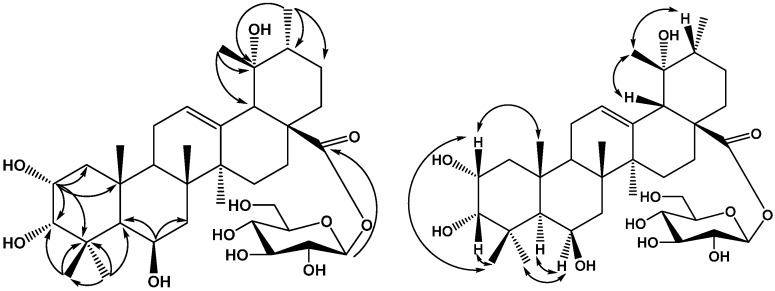
Key HMBC and NOESY correlations of compound **4**.

Compound **4** was obtained as a white amorphous powder, which gave a positive result in the Liebermann-Burchard test. Acid hydrolysis of compound **4** with 2 mol/L HCl/1,4-dioxane (1:1, *v/v*) furnished glucose, identified by TLC comparison with an authentic sample. The positive optical rotation (${\left[\alpha \right]}_{D}^{20}$ +45.7, *c* 0.03, H_2_O) indicated the d-configuration of glucose. The HR-ESI-MS of **4** showed a quasi-molecular ion peak at *m*/*z* 665.3901 [M−H]^−^, indicating a molecular formula of C_36_H_58_O_10_ (calcd. for C_36_H_57_O_10_, 665.3909, Δamu 1.2 ppm). The ^1^H and ^13^C-NMR spectra of **4** in pyridine-d_5_ showed typical signals for an ursane pentacyclic triterpenoid skeleton, including six tertiary methyl groups [δ_H_ 1.04, 1.07, 1.22, 1.25, 1.33, 1.38 (each 3H, s)] and one secondary methyl signals at δ_H_ 0.94 (3H, d, *J* = 6.6 Hz), as well as one olefinic proton at δ_H_ 5.36 (1H, br s), two olefinic carbons (δ_C_ 129.9 and 139.9) and an ester carbonyl carbon at δ_C_ 178.5. The ^1^H and ^13^C-NMR spectra of **4** exhibited three oxymethine protons at δ_H_ 4.38 (1H, s), 4.02 (1H, m), 3.29 (1H, d, *J* = 2.4 Hz) and one hydroxy group attached to a tertiary carbon. The data thus suggested that **4** is an ursane-type triterpene with four hydroxy groups, a trisubstituted double bond, and a carboxyl. Comparison of the NMR spectroscopic data of **4** with those of 2α,3β,19α-trihydroxyurs-12-en-28-O-β-d-glucopyranoside [7] demonstrated that two compounds were almost identical, except for an additional hydroxyl group at C-6 (δ_C_ 69.2). This was further confirmed by HMBC and NOESY experiments on **4**. The existence of four hydroxy groups at C-2, C-3, C-6 and C-19 was supported by the HMBC spectrum, HMBC correlations (Figure 5) were observed between H-1 (δ_H_ 1.54 and δ_H_ 1.28) and C-25 (δ_C_ 18.5), C-4 (δ_C_ 40.1), C-2 (δ_C_ 67.1), C-3 (δ_C_ 81.5); between H-2 (δ_H_ 4.02) and C-3 (δ_C_ 81.5); H-3 (δ_H_ 3.29) and C-2 (δ_C_ 67.1), C-4 (δ_C_ 40.1), C-24 (δ_C_ 24.4); between H-19 (δ_H_ 3.63) and C-21 (δ_C_ 29.5), C-17 (δ_C_ 47.0); between H-5 (δ_H_ 1.28), H-7 (δ_H_ 1.53) and C-6 (δ_C_ 69.2). The configuration of the hydroxyls at C-2, C-3, C-6 and C-19 were determined using NOESY correlations. The NOESY correlation of H-3 (δ_H_ 3.29) with H-24 (δ_H_ 1.25) indicated that the hydroxyl at C-3 should be in an α-orientation; the NOESY correlations of H-2 (δ_H_ 4.02) with H-24 (δ_H_ 1.25) and H-25 (δ_H_ 1.38) implied that the 2-OH group had an α-orientation; the NOESY correlations of H-6 (δ_H_ 4.38) with H-5 (δ_H_ 1.28) and H-23 (δ_H_ 1.07) implied that the 6-OH group had a β-orientation; the NOESY correlations of H-19 (δ_H_ 3.63) with H-30 (δ_H_ 1.66) the NOESY correlations of H-29 (δ_H_ 1.22) with H-18 (δ_H_ 2.54) and H-20 (δ_H_ 1.36) implied that the 19-OH group had an α-orientation. Therefore, the aglycon moiety of **4** was identified as 2α,3α,6β,19α-tetrahydroxyursolic acid. In the ^1^H-NMR spectrum of **4**, the relatively large ^3^*J*_H-1,H-2_ coupling constant of the anomeric proton at δ_H_ 5.32 of the d-glucopyranosyl moiety (*J* = 8.4 Hz) indicated a β-configuration for d-Glc. HMBC correlations between the anomeric proton at δ_H_ 5.32 (1H, d, *J* = 8.4 Hz) and the carbon signal at C-28 (δ_C_ 178.5) indicated that a β-d-glucopyranosyl moiety was attached to the C-28 position of the aglycone. On the basis of the foregoing evidence, the structure of **4** was determined as 2α,3α,6β,19α-tetrahydroxyursolic acid 28-*O*-β-d-glucopyranoside.

The structures of the three known triterpenoids 2α,3β,21β-trihydroxy ursolic acid 28-*O*-β-d-glucopyranoside (**5**) [8], 2α,3α,19α,23-tetrahydroxyoleanolic acid 28-*O*-β-d-glucopyranoside (**6**) [9] and 2α,3α,19α,23-tetrahydroxyursolic acid 28-*O*-β-d-glucopyranoside (**7**) [10] were determined by comparison of their NMR spectral data with those reported in the literature. 

## 3. Experimental Section

### 3.1. General Experimental Procedures

Optical rotations were measured on an Autopol IV-T/V (Rudolph Research Analytical, Hackettstown, NJ, USA). UV spectra were recorded in MeOH on a Jasco V650 spectrophotometer (JASCO, Inc., Easton, MD, USA). The ^1^H (600 MHz), ^13^C- (150 MHz), and 2D-NMR spectra were recorded on a Bruker AVANCE III 600 instrument using TMS (tetramethylsilane) as an internal reference (Bruker Company, Billerica, MA, USA). HRTOFMS data were obtained on an Agilent 7890–7000 A mass spectrometer (Agilent Technologies, Santa Clara, CA, USA). Preparative HPLC (high-performance liquid chromatography) was conducted with an Agilent Technologies 1200 series instrument with an multiple wavelength detector using a YMC-pack ODS (Octadecylsilyl)-A column (5 µm, 250 × 20 mm). Column chromatography was performed with silica gel (200–300 mesh, Qingdao Marine Chemical Ltd., Qingdao, China), Develosil ODS (50 µm, Nomura Chemical Co. Ltd., Osaka, Japan), and Sephadex LH-20 GE Healthcare Bio-Sciences AB, Uppsala, Sweden). TLC (thin layer chromatography) was carried out with glass precoated with silica gel GF254. Spots were visualized under UV light or by spraying with 10% sulfuric acid in EtOH followed by heating. All reagents and solvents are analystical grade.

### 3.2. Plant Material

The aerial parts of *C. kwangtungensis* Chun were collected from Pingxiang, Jiangxi province of China, in July 2012. The plant was identified by Guiping Yuan at Jiangxi Provincial Institute for Drug and Food Control, China. A voucher specimen (No. 20120715) is deposited in the Herbarium of Jiangxi Provincial Institute for Drug and Food Control.

### 3.3. Extraction and Isolation

The aerial parts of *C. kwangtungensis* (10.5 kg) were extracted three times with 95% EtOH under reflux (2 h each). The extracted solution was evaporated under reduced pressure to yield a dark-brown residue (1.2 kg). The residue was suspended in water (20 L) and then successively partitioned with petroleum ether (3 × 20 L), EtOAc (3 × 20 L), and *n*-BuOH (3 × 20 L). After removing the solvent, the EtOAc-soluble portion (130 g) was fractionated via silica gel column chromatography (CC), eluting with CHCl_3_/MeOH (5:1, *v*/*v*), to give 10 major fractions A1-A10 on the basis of TLC analysis. Fraction A2 (7.8 g) was subjected to silica gel CC and eluted with CHCl_3_/MeOH (30:1–1:1, *v*/*v*) to afford nine fractions (A2-1-A2-9). Fraction A2-4 (2.5 g) was separated by ODS CC (50 µm, 20%–100%, MeOH/H_2_O) to give four subfractions (A2-4-1-A2-4-4). Subfraction A2-4-3 (1.1 g) was separated by Sephadex LH-20 CC using MeOH to afford five fractions (A2-4-3-1–A2-4-3-5) on the basis of TLC analysis. Fraction A2-4-3-3 (108 mg) was further separated by preparative HPLC (YMC-ODS-A, 5 µm, 250 mm × 20 mm, detection at 210 nm) using 23% CH_3_CN–H_2_O (7 mL/min) as mobile phase to yield **1** (20.2 mg) and **2** (4.0 mg). A2-4-2 (1.1 g) was subjected to silica gel CC and eluted with CHCl_3_/MeOH (12:1–4:1) to afford three fractions (A2-4-2-1-A2-4-2-3). Subfraction A2-4-2-2 (108 mg) was separated by preparative HPLC (YMC-ODS-A, 5 µm, 250 mm × 20 mm, detection at 210 nm) using 23% CH_3_CN–H_2_O (7 mL/min) to yield **3** (4.5 mg), **4** (21.3 mg) and **5** (5.8 mg). Fraction 5 (8.9 g) was subjected to silica gel CC and eluted with CHCl_3_/MeOH (30:1–1:1, *v*/*v*) to afford five fractions (A5-1-A5-5). A5-4 (2.7 g) was subjected to ODS CC (50 µm, 20%–100%, MeOH-H_2_O) to afford four subfractions (A5-4-1-A5-4-4). A5-4-3 (1.31 g) was subjected to silica gel CC and eluted with CHCl_3_/MeOH (30:1–1:1, *v*/*v*) to afford three fractions (A5-4-3-1~A5-4-3-3), A5-4-3-1 (217 mg) separated by preparative HPLC (YMC-ODS-A, 5 µm, 250 mm × 20 mm, detection at 210 nm) using 23% CH_3_CN–H_2_O (7 mL/min) to yield **6** (5.3 mg)and **7** (16.8 mg).

### 3.4. Acid Hydrolysis of Compounds **1**–**4**

Compounds **1** (1.0 mg), **2** (1.0 mg), **3** (1.0 mg) and **4** (1.0 mg) were heated in an ampule with aqueous 2 mol/L HCl/1,4-dioxane (1:1, 2 mL) at 80 °C for 6 h. The aglycone was extracted with chloroform (3 × 3 mL). The aqueous layer was evaporated under reduced pressure and subjected to the column chromatography over Sephadex LH-20, eluting with CH_3_CN/H_2_O (8:1) to yield the sugar residue. Compound **1**, **2**, **3** and **4** gave d-glucose which was identified by TLC comparison with a standard sample (CH_3_CN/H_2_O (6:1); R*_f_* = 0.35 and its positive optical rotation. 

### 3.5. The Physicochemical Data of Compounds **1**–**7**

*2α,3β,6β,19α-Tetrahydroxyoleanolic Acid 28-O-β-d-Glucopyranoside* (**1**). White amorphous powder; ${\left[\alpha \right]}_{D}^{20}$ –12.5 (*c* 0.12, MeOH); UV (MeOH) λ_max_ (log*ε*): 207.6 (3.33) nm; for ^1^H-NMR (600 MHz, C_5_D_5_N) and ^13^C-NMR (150 MHz, C_5_D_5_N) spectral data, see Table 1; HR-ESI-MS *m*/*z* 665.3901 [M−H]^−^, (calcd for C_36_H_57_O_10_, 665.3909, Δamu 2.6 ppm).

*2-O-β-d-Glucopyranosyloxy-3α,19α-dihydroxyoleanolic Acid* (**2**). White amorphous powder; ${\left[\alpha \right]}_{D}^{20}$ −23.3 (*c* 0.03, MeOH); UV (MeOH) λ_max_ (log*ε*): 206.2 (3.22) nm; for ^1^H-NMR (600 MHz, C_5_D_5_N) and ^13^C-NMR (150 MHz, C_5_D_5_N) spectral data, see Table 1; HR-ESI-MS *m*/*z* 649.3962 [M−H]^−^ (calcd for C_36_H_57_O_10_, 649.3968, Δamu 0.9 ppm).

**Table 1:** ^1^H-NMR (600 MHz) and ^13^C-NMR (150 MHz) spectral data of **1**–**4** (δ in ppm, *J* in Hz, in pyridine-*d*_5_).

No.	1		2		3		4	
	C	H	C	H	C	H	C	H
1	50.4	1.40, 1H, br t, *J* = 12.0 Hz	39.8	1.86, 1H, br t, *J* = 12.0 Hz	39.9	1.80, 1H, br t, *J* = 12.0 Hz	44.9	1.28, 1H, s
		2.35, 1H, m		2.00, 1H, m		1.94, 1H, m		1.54, 1H, m
2	69.3	4.30, 1H, m	76.9	4.46, 1H, m	76.6	4.46, 1H, m	67.1	4.02, 1H, m
3	84.4	3.43, 1H, d, *J* = 9.0 Hz	79	4.05,1H, d, *J* = 2.4 Hz	79.1	4.03, 1H, d, *J* = 2.4 Hz	81.5	3.29, 1H, d, *J* = 2.4 Hz
4	40.2		39.2		39.0		40.1	
5	57.4	1.21, 1H, s	49.5	1.68, 1H, m	48.8	1.66, 1H, m	49.7	1.28, 1H, s
6	68.3	4.87, 1H, s	19.1	1.34, 1H, m	19.0	1.33, 1H, m	69.2	4.38, 1H, s
				1.49 m		1.47 m		
7	41.3	2.00, 2H, m	34.1	1.36, 1H, m	33.9	1.35, 1H, m	40.6	1.53, 2H, m
				1.58, 1H, m		1.65, 1H, m		
8	42.1		40.7		41.0		41.7	
9	49.5	2.14, 1H, m	48.7	2.08, 1H, m	48.1	2.03, 1H, m	49.5	1.90, 1H, m
10	39.0		39.2		39.0		38.3	
11	24.8	2.33, 1H, m	24.8	2.01, 1H, m	24.6	2.00, 1H, s	24.4	2.09, 2H, m
12	124.5	5.61, 1H, s	124.3	5.57, 1H, s	128.4	5.56, 1H, s	129.9	5.36, 1H, s
13	144.2		145.3		140.4		139.9	
14	43.3		42.7		42.7		43.2	
15	29.6	1.32, 1H, m	29.7	1.33, 1H, m	29.7	1.25, 1H, m	29.3	1.03, 1H, m
		2.08, 1H, m		2.12, 1H, m		2.33, 1H, m		1.91, 1H, m
16	28.7	2.17, 1H, m	28.9	2.16, 1H, m	27.4	1.33, 1H, m	27.1	1.26, 1H, m
		2.83, 1H, m		2.80, 1H, m		2.08, 1H, m		1.77, 1H, m
17	47.0		46.6		49.3		49.6	
18	45.1	3.58, 1H, s	45.3	3.60, 1H, m	55.0	3.05, 1H, t	55.0	2.54, 1H, s
19	81.6	3.63, 1H, s	81.8	3.63, 1H, t	73.2		73.7	
20	36.0		36.2		42.8	1.50, 1H, m	43.0	1.36, 1H, m
21	29.5	1.07, 1H, m	29.6	1.25, 1H, m	26.9	2.04, 1H, m	26.6	1.64, 1H, m
		2.48, 1H, t *J* = 6.0 Hz		1.35, 1H, m		3.11, 1H, m		2.62, 1H, m
22	33.4	2.00, 1H, m	33.8	2.04, 1H, m	39.2	2.08, 1H, m	38.8	1.66, 1H, m
		2.08, 1H, m		2.17, 1H, m		2.16, 1H, m		1.79, 1H, m
23	29.6	1.49, 3H, s	29.9	1.23, 3H, s	29.8	1.22, 3H, s	29.6	1.07, 3H, s
24	19.0	1.79, 3H, s	22.8	0.88, 3H, s	22.8	0.86, 3H, s	24.4	1.25, 3H, s
25	18.9	1.79, 3H, s	17.0	0.97, 3H, s	17.0	0.96, 3H, s	18.5	1.38, 3H, s
26	19.8	1.83, 3H, s	18.1	1.06, 3H, s	17.7	1.09, 3H, s	18.7	1.04, 3H, s
27	25.3	0.99, 3H, s	25.3	1.54, 3H, s	25.1	1.62, 3H, s	24.7	1.33, 3H, s
28	177.7		181.3		181.1		178.5	
29	29.2	1.17, 3H, s	29.3	1.19, 3H, s	27.5	1.44, 3H, s	27.2	1.22, 3H, s
30	25.3	1.66, 3H, s	25.3	1.54, 3H, s	17.3	1.14, 3H, d, *J* = 6.6 Hz	16.6	0.94, 3H, d, *J* = 6.6 Hz
Glc								
1	96.4	6.36, 1H, d, *J* = 7.8 Hz	104.2	5.16, 1H, d, *J* = 7.2 Hz	104.1	5.14, 1H, d, *J* = 7.8 Hz	95.9	5.32, 1H, d, *J* = 7.8 Hz
2	74.6	4.24, 1H, t, *J* = 7.8 Hz	75.8	4.07,1H, t, *J* = 7.2 Hz	75.9	4.07, 1H, t, *J* = 7.8 Hz	73.9	3.35, 1H, t, *J* = 7.8 Hz
3	79.7	4.02, 1H, d, *J* = 9.0 Hz	78.3	4.28,1H, d, *J* = 9.0 Hz	78.3	4.29, 1H, d, *J* = 8.4 Hz	78.3	3.35, 1H, d, *J* = 8.4 Hz
4	71.7	4.39, 1H, t, *J* = 9.0 Hz	72.3	4.30,1H, t, *J* = 9.0 Hz	72.2	4.31, 1H, t, *J* = 8.4 Hz	71.3	3.40, 1H, t, *J* = 8.4 Hz
5	79.3	4.30, 1H, t, *J* = 9.0 Hz	78.8	4.32, 1H, t, *J* = 9.0 Hz	78.9	4.33, 1H, t, *J* = 8.4 Hz	78.6	4.03, 1H, dd, *J* = 8.4 Hz *J* = 4.2 Hz
6	62.6	4.41, 1H, t, *J* = 9.6 Hz	63.3	4.37, 1H, dd, *J* = 4.8 Hz *J* = 12.0 Hz	63.2	4.37, 1H, dd, *J* = 8.4 Hz *J* = 11.4 Hz	62.4	3.70, 1H, dd, *J* = 4.2 Hz *J* = 12.0 Hz
		4.44, 1H, m		4.54, 1H, dd, *J* = 2.4 Hz *J* = 12.0 Hz		4.54, 1H, dd, *J* = 2.4 Hz *J* = 11.4 Hz		3.81, 1H, dd, *J* = 1.8 Hz *J* = 12.0 Hz

*2-O-β-d-Glucopyranosyloxy-3α,19α-dihydroxyursolic Acid* (**3**). White amorphous powder; ${\left[\alpha \right]}_{D}^{20}$ −13.0 (*c* 0.1, MeOH); UV (MeOH) λmax (logε): 208.6 (3.16) nm; for ^1^H-NMR (600 MHz, C_5_D_5_N) and ^13^C-NMR (150 MHz, C_5_D_5_N) spectral data, see Table 1; HR-ESI-MS *m*/*z* 649.3952 [M−H]^−^ (calcd for C_36_H_57_O_10_, 649.3958, Δamu 0.41 ppm).

*2α,3α,6β,19α-Tetrahydroxyursolic Acid 28-O-β-d-Glucopyranoside* (**4**). White amorphous powder; ${\left[\alpha \right]}_{D}^{20}$ −15.0 (*c* 0.08, MeOH); UV (MeOH) λ_max_ (log*ε*): 210.1 (3.55) nm; for ^1^H-NMR (600 MHz, C_5_D_5_N) and ^13^C-NMR (150 MHz, C_5_D_5_N) spectral data, see Table 1; HR-ESI-MS *m*/*z* 665.3901 [M–H]^−^ (calcd for C_36_H_57_O_10_, 665.3909, Δamu 1.2 ppm).

*2α,3β,21β-Trihydroxyursolic acid 28-O-β-d-glucopyranoside* (**5**), 2α,3α,19α,23-tetrahydroxyoleanolic acid 28-*O*-β-d-glucopyranoside (**6**), and 2α,3α,19α,23-tetrahydroxyursolic acid 28-*O*-β-d-glucopyranoside (**7**), for ^1^H-NMR (600 MHz, C_5_D_5_N) and ^13^C-NMR (150 MHz, C_5_D_5_N) spectral data, see Table 2.

**Table 2:** ^1^H-NMR (600 MHz) and ^13^C-NMR (150 MHz) spectral data of **5**–**7** (δ in ppm, *J* in Hz, in pyridine-*d*_5_).

Position	5		6		7	
1	46.7	0.95 (1H, m)	42.7	1.90 (1H, m)	48	1.23 (1H, m)
		2.25 (1H, m)		2.00 (1H, m)		2.08 (1H, m)
2	67.2	3.31 (1H, m)	66.7	4.08 (1H, m)	69.4	4.10 (1H, m)
3	83.1	2.95 (1H, d, *J* = 9.6 Hz)	79.9	3.78 (1H, d, *J* = 2.4 Hz)	78.8	3.76 (1H, d, *J* = 3.6 Hz)
4	39		42.6		44.2	
5	55.6	0.93 (1H, m)	44	1.41 (1H, s)	48.5	1.33 (1H, m)
		1.23 (1H, m)	19	1.21 (1H, m)		
6	18.5	1.38 (1H, m)		1.34 (1H, m)	19.3	1.48 (2H, m)
		1.25 (1H, m)	33.7	1.25 (1H, m)		
7	32.8	1.42(1H, m)		1.40 (1H, m)	33.9	1.70 (2H, m)
8	39		41.2		40.8	
9	47.8	1.93 (1H, m)	48.3	2.06 (1H, m)	49	2.03 (1H, m)
10	37.7		38.2		39.1	
11	23.3	2.16 (1H, m)	25	2.00 (1H, m)	24.8	2.01 (1H, m)
12	122.3	4.76 (1H, d, *J =* 4.5 Hz)	128.7	5.56 (1H, br s)	1283.6	5.54 (1H, br s)
13	143.7		139.6		144.8	
14	41.4		42.7		42.7	
15	28.5	0.98 (1H, m)	29.7	1.34 (1H, m)	29.7	1.31 (1H, m)
		1.83 (1H, m)		2.10 (1H, m)		2.04 (1H, m)
16	24.3	1.06 (1H, m)	27.1	2.14 (1H, m)	26.7	2.08 (1H, m)
		1.68 (1H, m)		2.62 (1H, m)		2.74 (1H, m)
17	47		49.1		47	
18	41.8	2.52 (1H, s)	54.9	2.52 (1H, s)	45.1	3.52 (1H, s)
19	46.6	1.06 (1H, m)	73.1		81.5	3.57 (1H, s)
		2.16 (1H, m)				
20	36		42.1	1.43 (1H, m)	36	
21	71.4	3.53 (1H, m)	26.6	2.00 (1H, m)	29.5	1.88 (2H, m)
				3.13 (1H, m)		
22	41.5	1.91 (1H, m)	38.9	2.04 (1H, m)	33.5	2.04 (1H, m)
		2.22 (1H, m)		2.14 (1H, m)		2.10 (1H, m)
23	29.5	0.84 (3H, s)	71	3.75 (1H, d, 10.8 Hz)	67	3.57 (1H, d, 10.2 Hz)
				3.92 (1H, d, 10.8 Hz)		3.73 (1H, d, 10.2 Hz)
Table 2. *Cont*. 24	17.3	1.06 (3H, s)	18.2	0.90 (3H, s)	14.7	0.99 (3H, s)
25	16.7	1.00 (3H, s)	17.6	1.09 (3H, s)	17,8	1.22 (3H, s)
26	17	0.91 (3H, s)	18	1.26 (3H, s)	18.2	1.10 (3H, s)
27	25.8	1.41 (3H, s)	27.5	1.65 (3H, s, )	25.9	1.56 (3H, s)
28	176.6		177.9		177.8	
29	29.7	1.12 (3H, s)	24.7	1.39 (3H, s)	29.4	1.15 (3H, s)
30	20.1	1.09 (3H, s)	17.2	1.07 (3H, d, *J* = 6.6 Hz)	25.1	1.17 (3H, s)
Glc						
1′	96.6	5.44 (1H, d, *J* = 7.8 Hz)	96.4	6.30 (1H, d, *J* = 7.8 Hz)	96.4	6.30 (1H, d, *J* = 7.8 Hz)
2′	74	3.33 (1H, m)	74.6	4.22 (1H, m)	74.6	4.21 (1H, m)
3′	78.9	3.43 (1H, m)	79.8	4.02 (1H, m)	79.9	3.98 (1H, m)
4′	71.3	3.3 (1H, m)	71.7	4.33 (1H, m)	71.6	4.32 (1H, m)
5′	79.4	3.31 (1H, m)	79.5	4.27 (1H, m)	79.8	4.25 (1H, m)
6′	61.9	3.64 (1H, m)	62.7	4.35 (1H, m)	62.7	4.37 (1H, m)
		3.85 (1H, m)		4.37 (1H, m)		4.40 (1H, m)

## 4. Conclusions

Four new triterpenoids which were identifed as 2α,3β,6β,19α-tetrahydroxyoleanolic acid 28-*O*-β-d-glucopyranoside (**1**), 2-*O*-β-d-glucopyranosyloxy-3α,19α-dihydroxyoleanolic acid (**2**), 2-*O*-β-d-glucopyranosyloxy-3α,19α-dihydroxyursolic acid (**3**) and 2α,3α,6β,19α-tetrahydroxyursolic acid 28-*O*-β-d-glucopyranoside (**4**), were isolated together with three known triterpenoids identified as 2α,3β,21β-trihydroxyursolic acid 28-*O*-β-d-glucopyranoside (**5**), 2α,3α,19α,23-tetrahydroxyoleanolic acid 28-*O*-β-d-glucopyranoside (**6**), and 2α,3α,19α,23-tetrahydroxyursolic acid 28-*O*-β-d-glucopyranoside (**7**) from the aerial parts of *Callicarpa kwangtungensis*. This finding represents an addition to the ongoing research on the pharmacological activity of this plant, which may be helpful to understand the use of *Callicarpa kwangtungensis* in traditional medicine and should continue to clarify its actual health benefits.

## Supplementary Materials

Supplementary materials can be accessed at: http://www.mdpi.com/1420-3049/20/05/9071/s1.
