# Supplementary material for: Four New Triterpenoids from Callicarpa kwangtungensis
Source: Molecules. 2015 May 19;20(5):9071–83. doi: 10.3390/molecules20059071 (PMC6272150; doi:10.3390/molecules20059071)
Supplement: Supplementary file 1 [file molecules-20-09071-s001.pdf]

# Supplementary Materials

## 1. The Spectrum of Compound 1

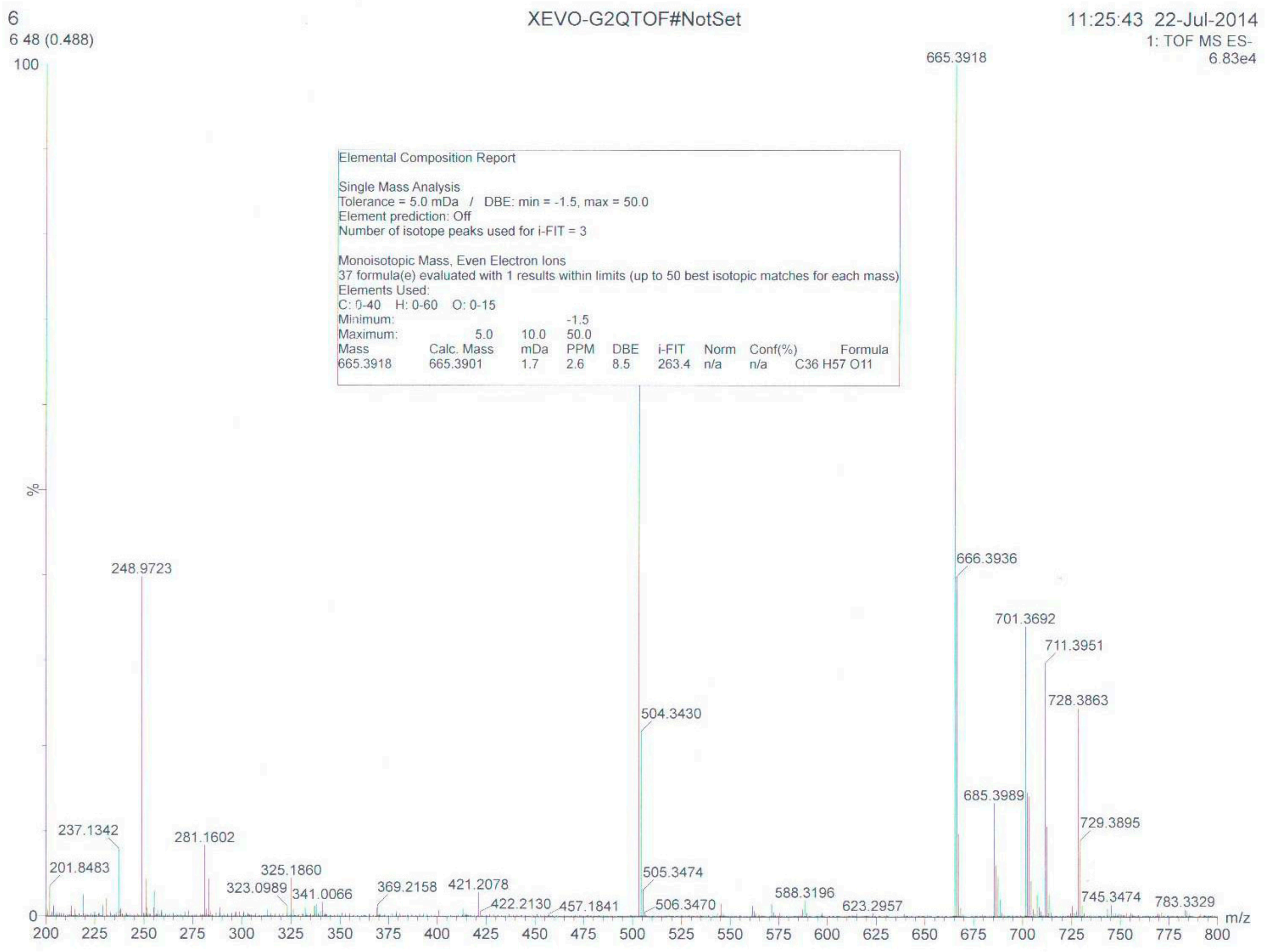


**Figure S1-1.** The HR-ESI-MS Spectrum of Compound **1**.

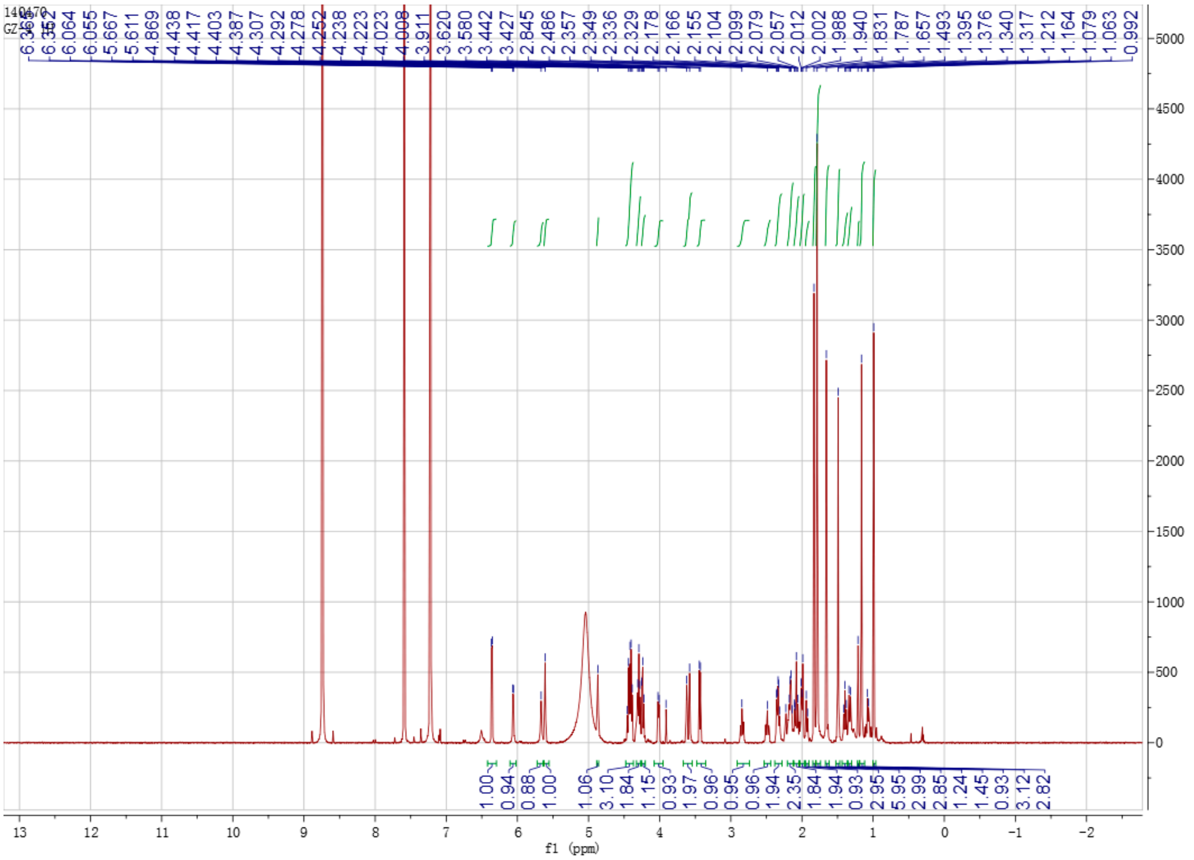

**Figure S1-2.** The Whole $^1$H-NMR Spectrum of Compound **1**.

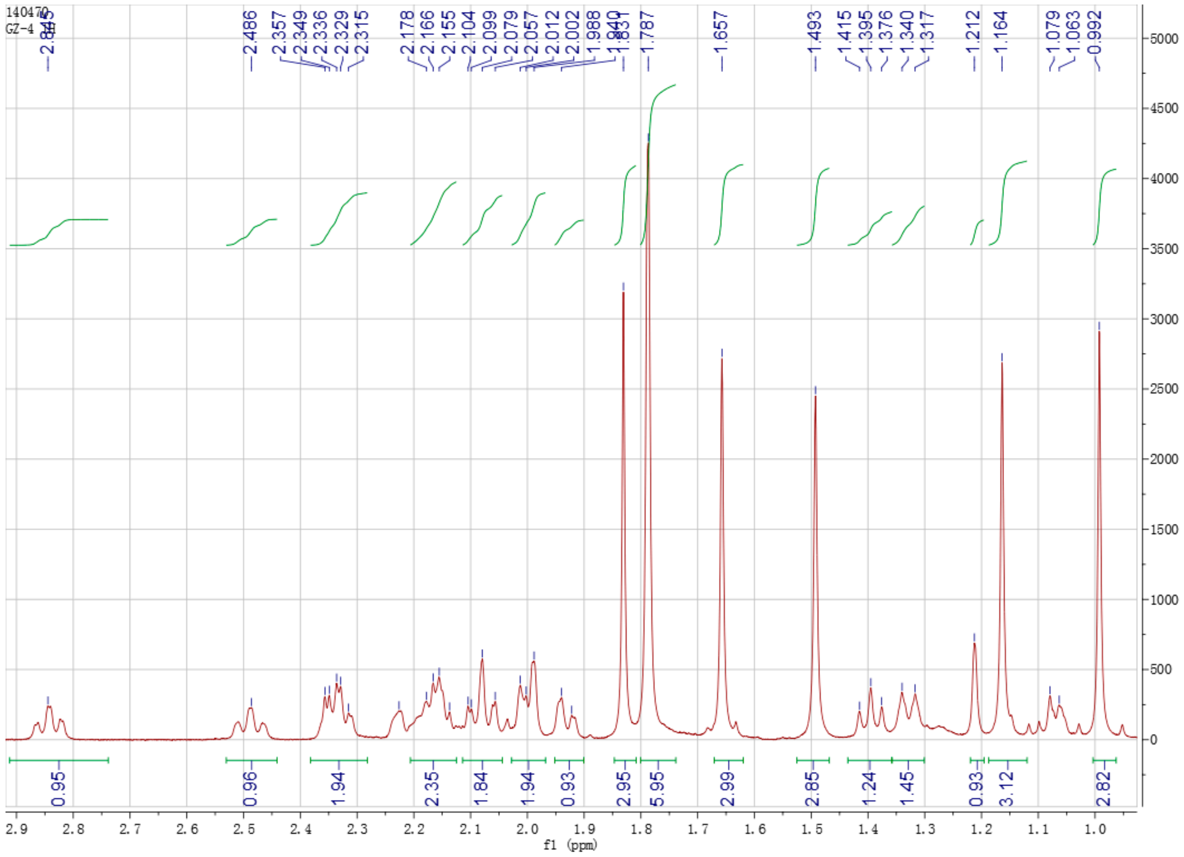

**Figure S1-3.** The Part 1 of the $^1$H-NMR Spectrum of Compound **1**.

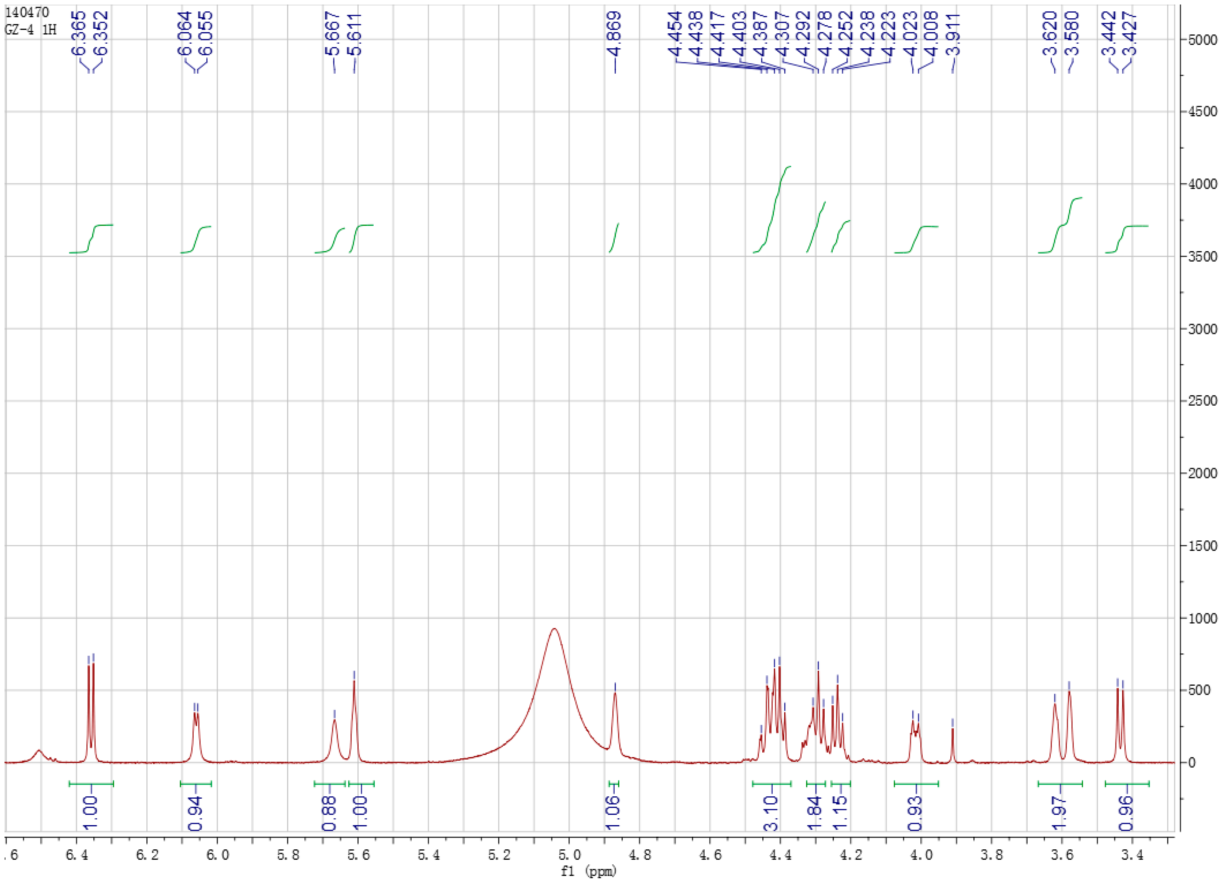


**Figure S1-4.** The Part 2 of the $^{1}$H-NMR Spectrum of Compound **1**.

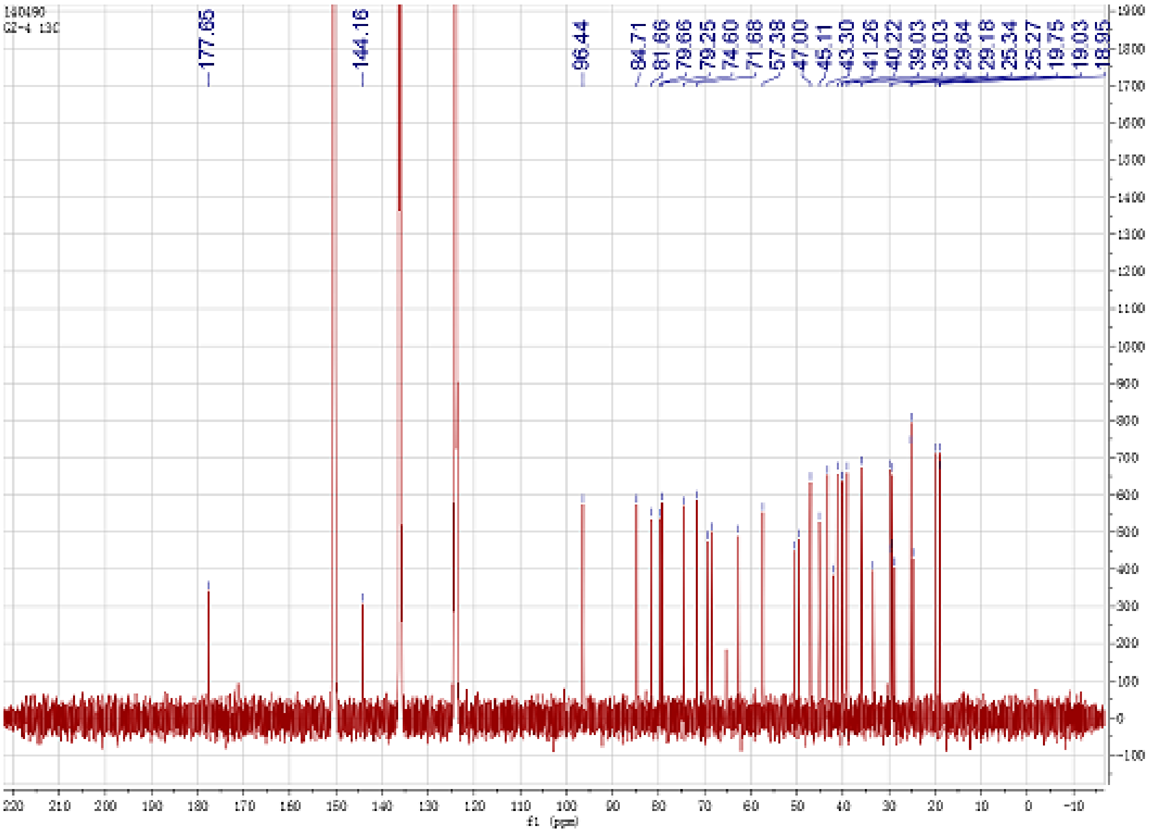


**Figure S1-5.** The $^{13}$C-NMR Spectrum of Compound **1**.

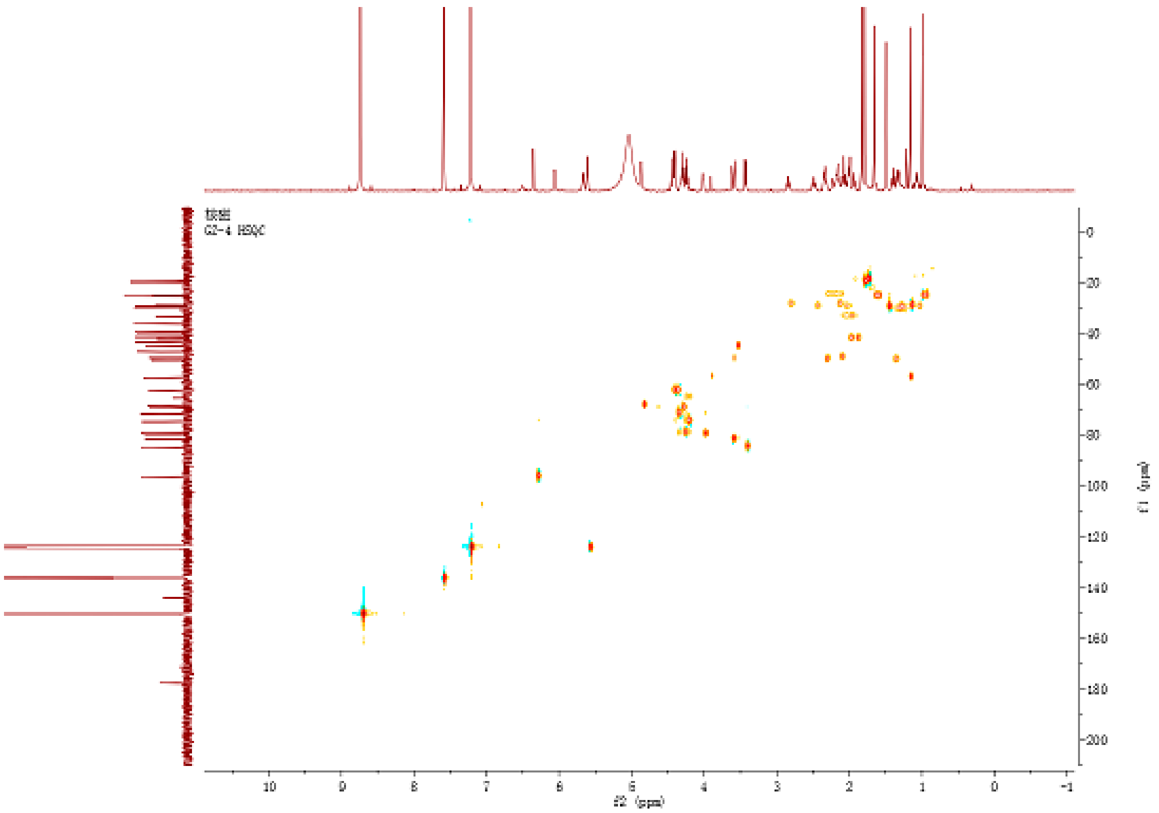


**Figure S1-6.** The Whole HSQC Spectrum of Compound **1**.

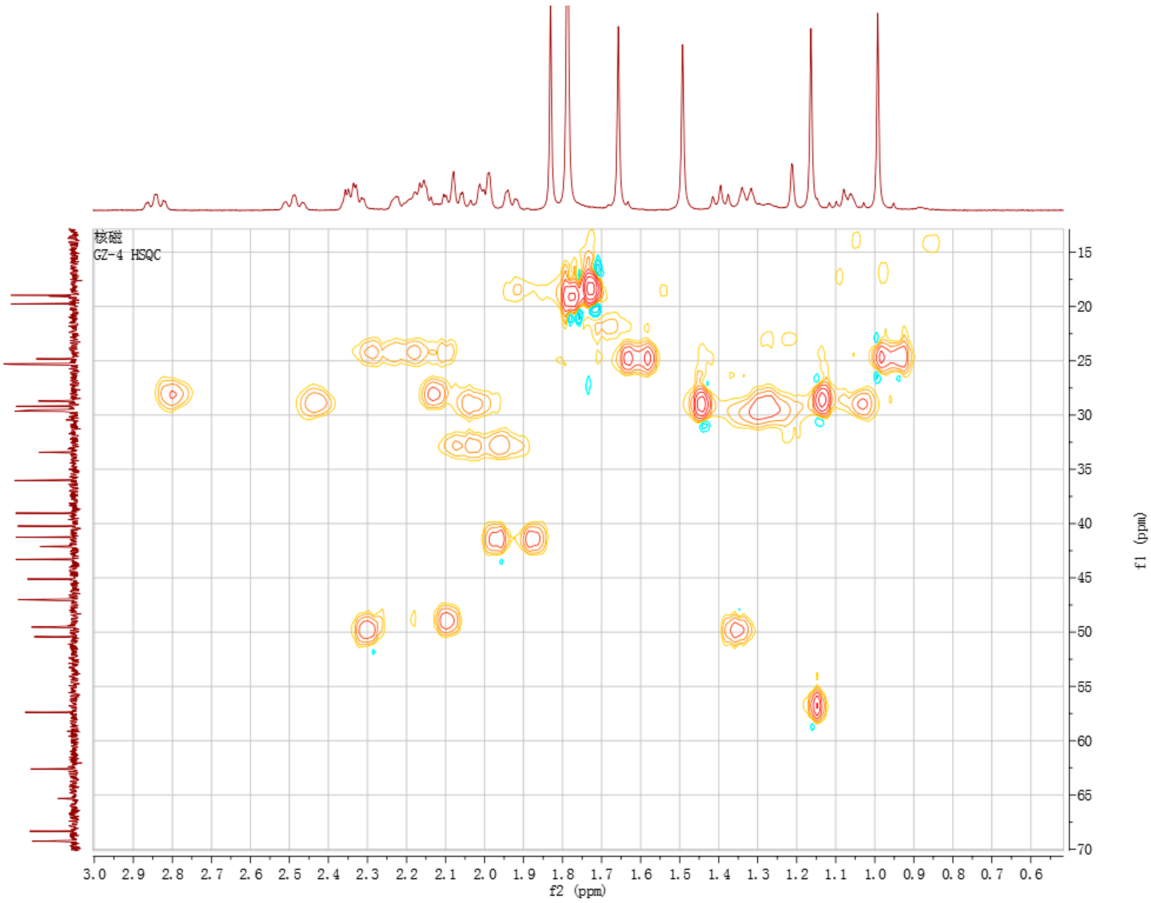


**Figure S1-7.** The Part 1 of the HSQC Spectrum of Compound **1**.

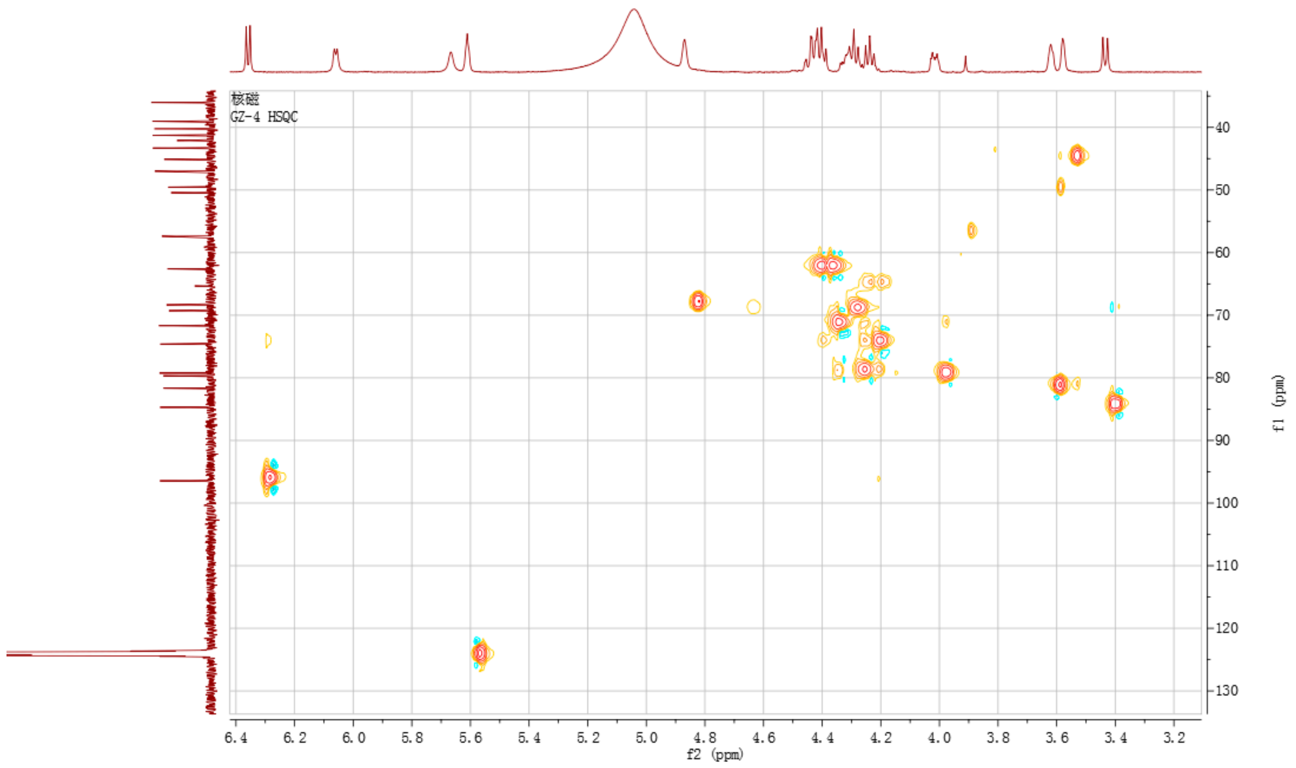


**Figure S1-8.** The Part 2 of the HSQC Spectrum of Compound **1**.

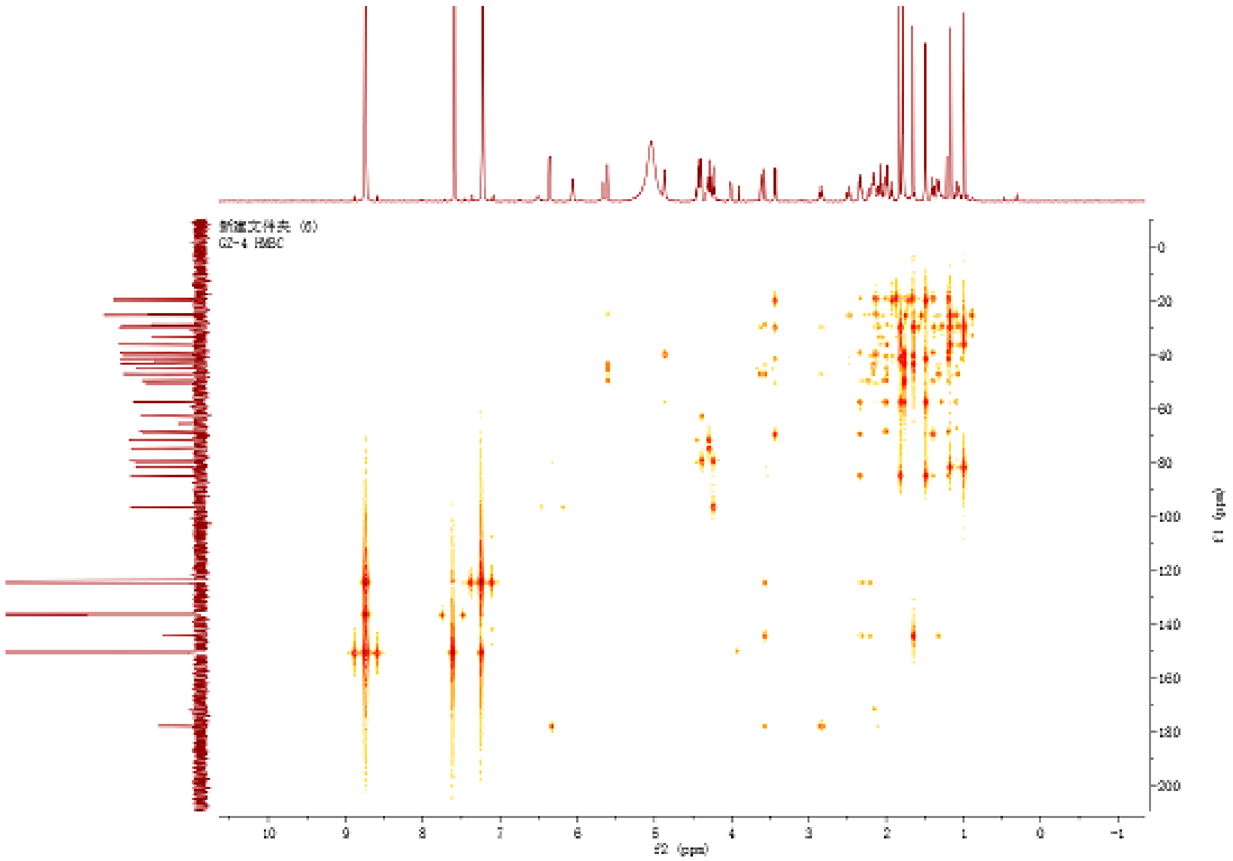


**Figure S1-9.** The Whole HMBC Spectrum of Compound **1**.

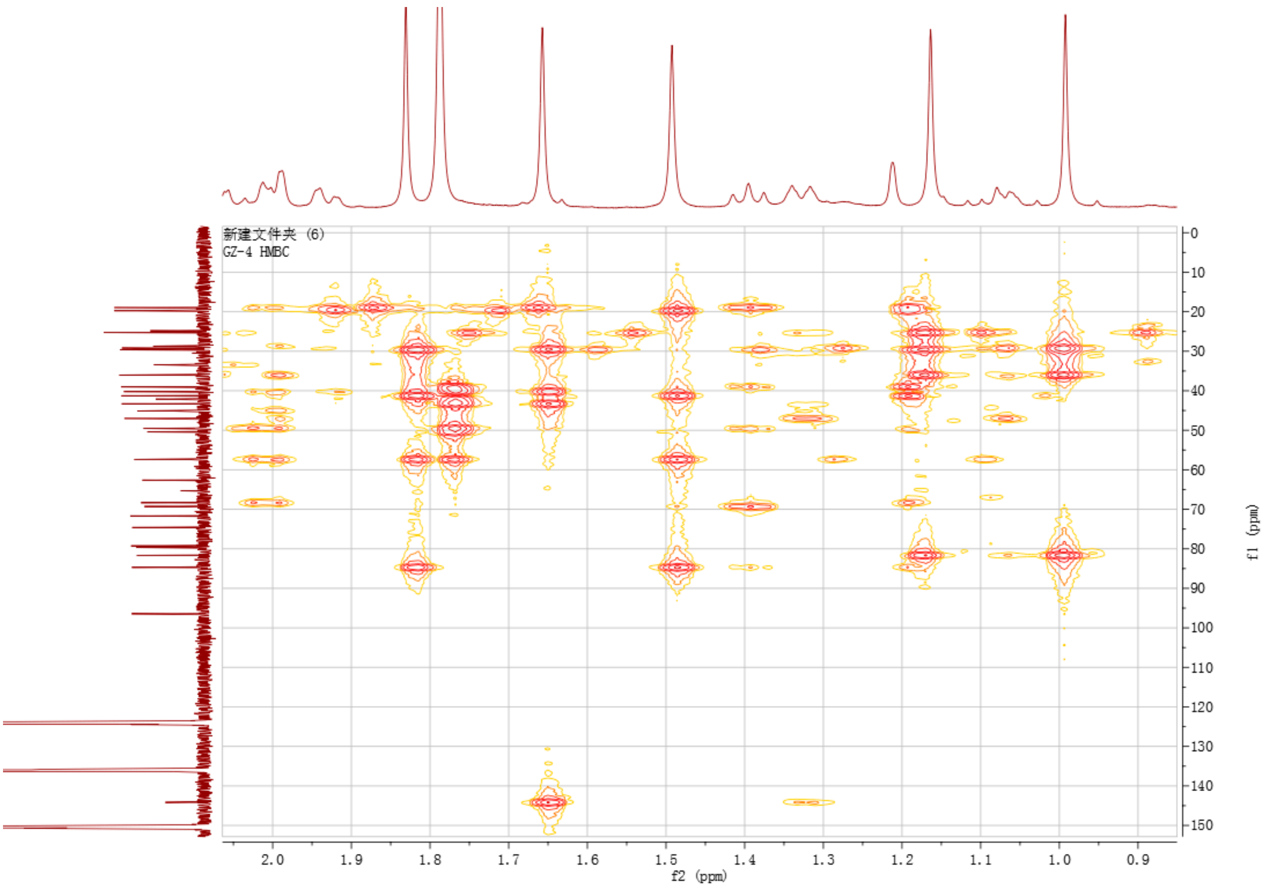


**Figure S1-10.** The Part 2 of HMBC Spectrum of Compound **1**.

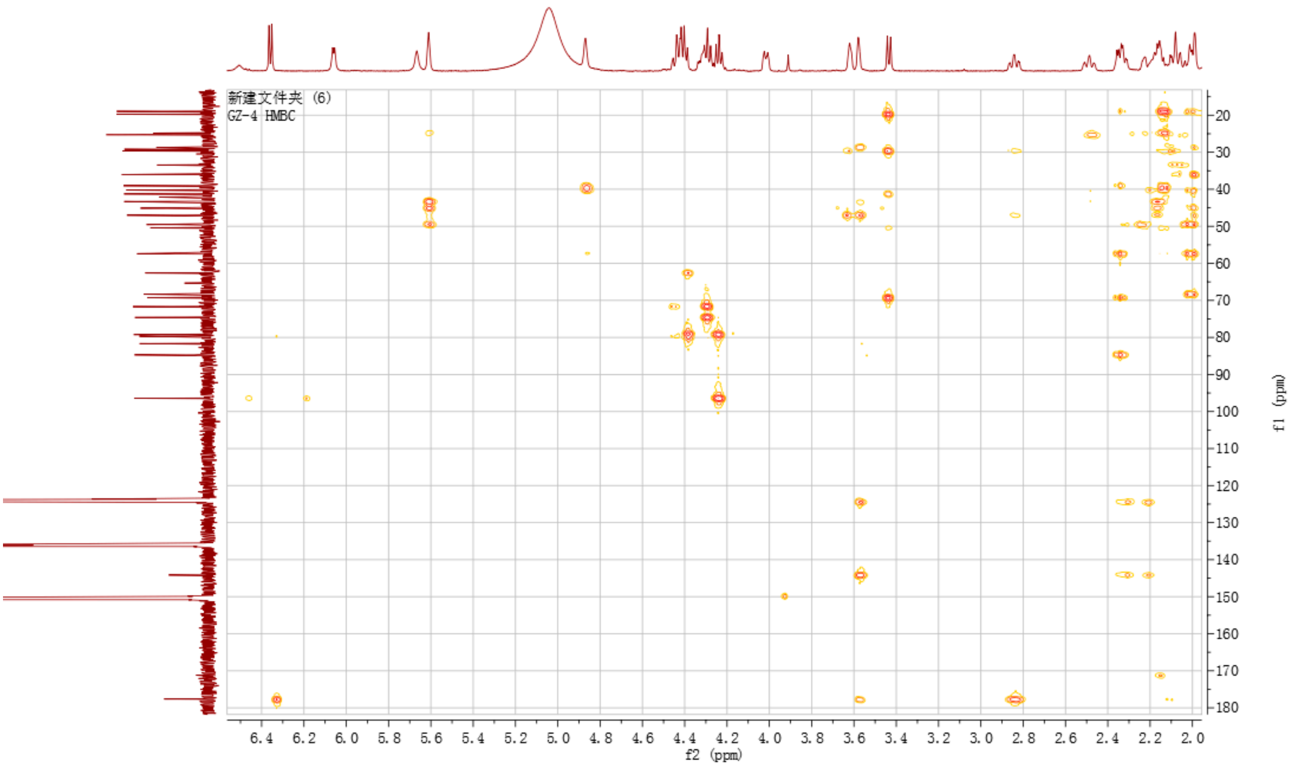


**Figure S1-11.** The Part 1 of the HMBC Spectrum of Compound **1**.

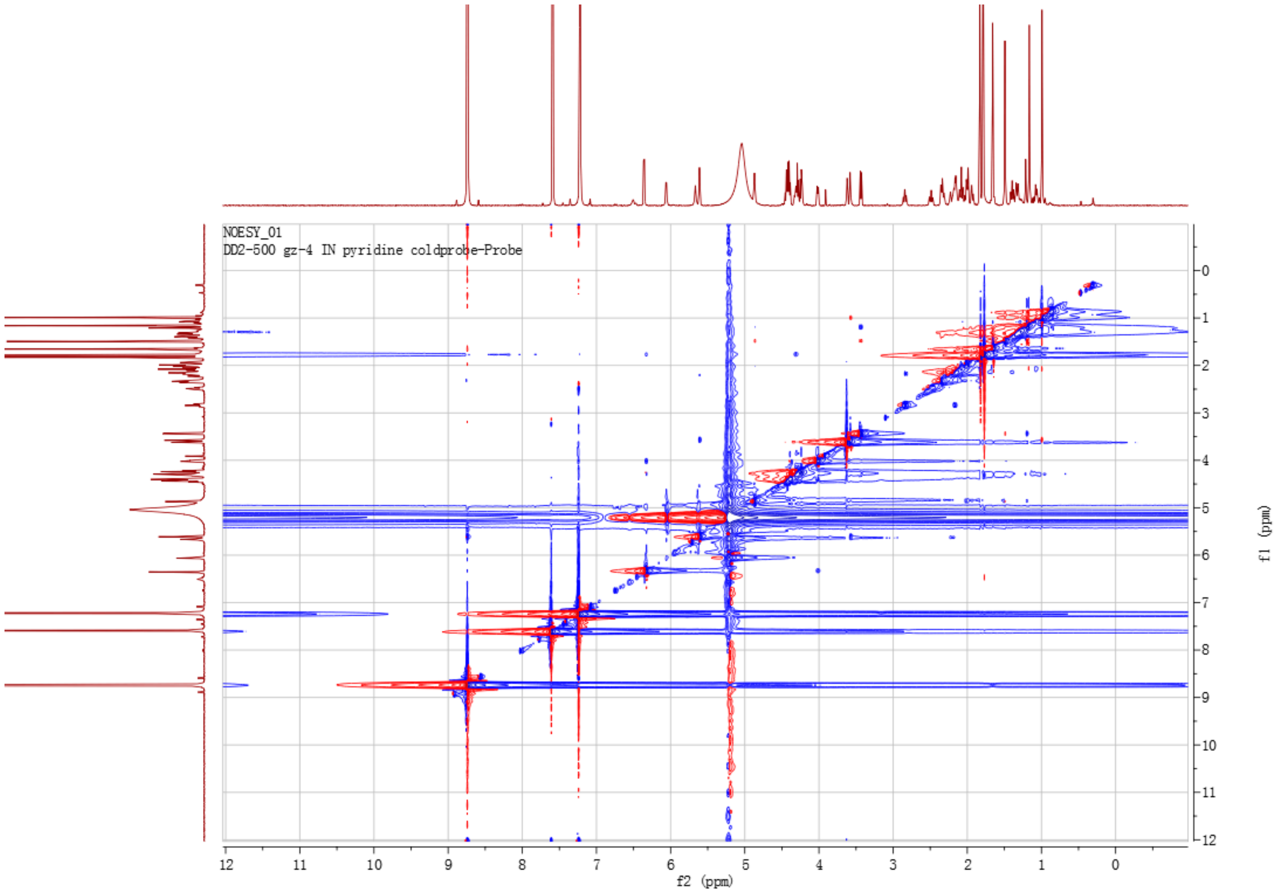


**Figure S1-12.** The Whole NOESY Spectrum of Compound **1**.

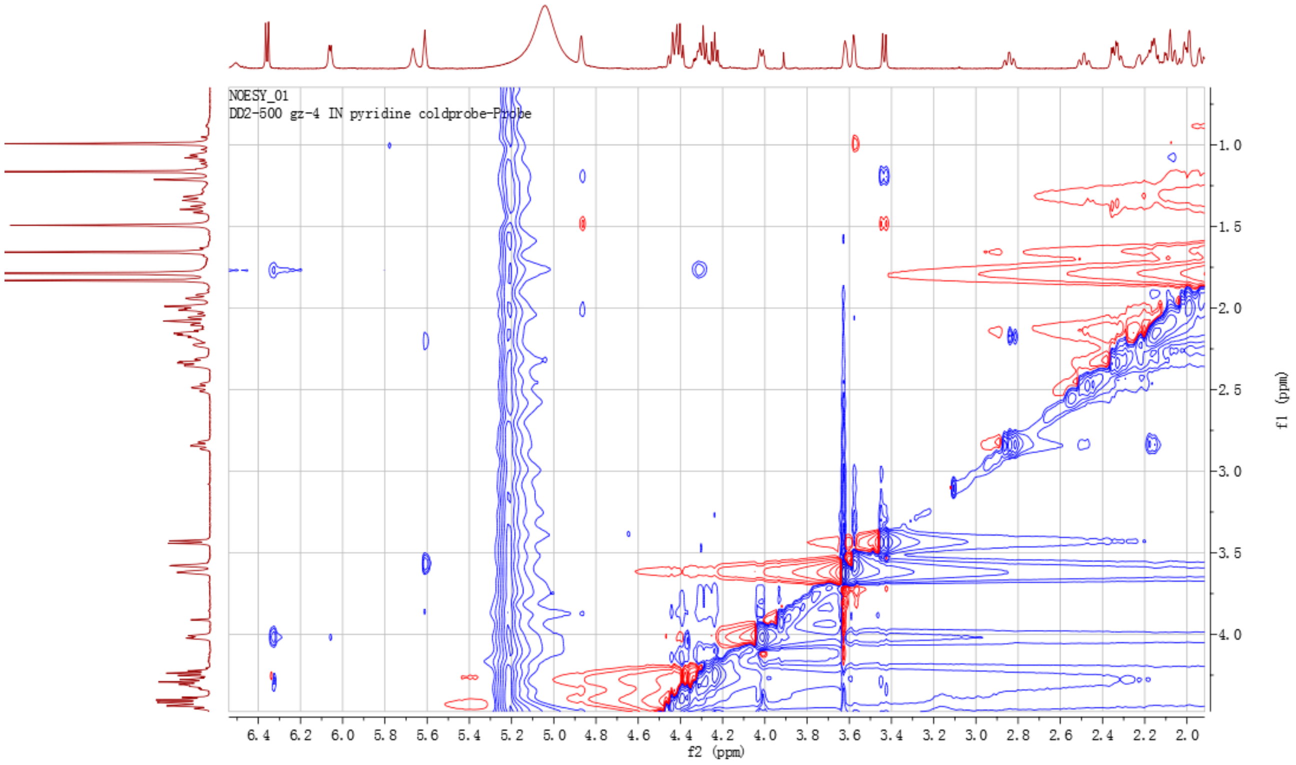


**Figure S1-13.** The Part NOESY Spectrum of Compound **1**.

## 2. The Spectrum of Compound 2

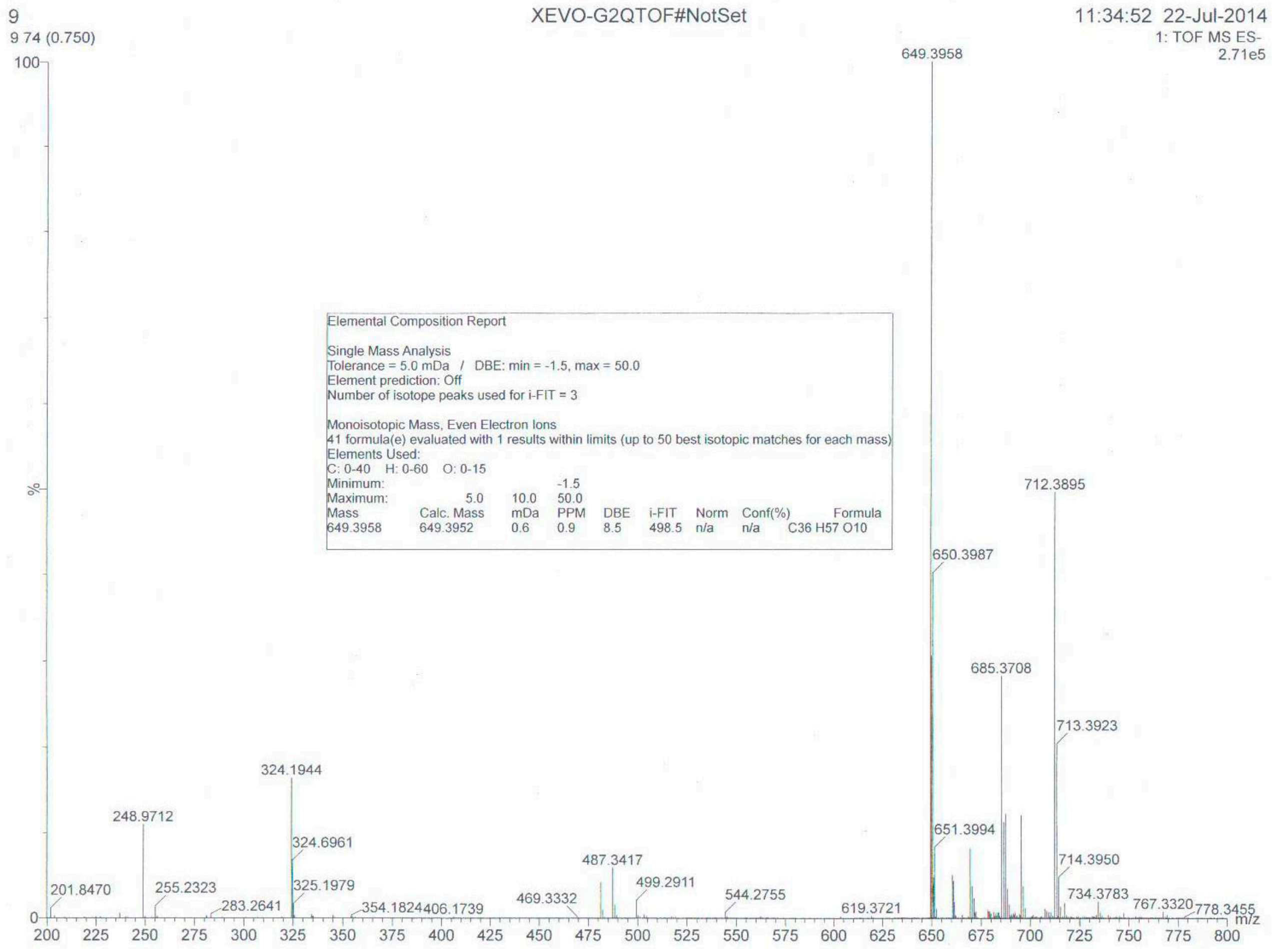


**Figure S2-1.** The HR-ESI-MS Spectrum of Compound **2**.

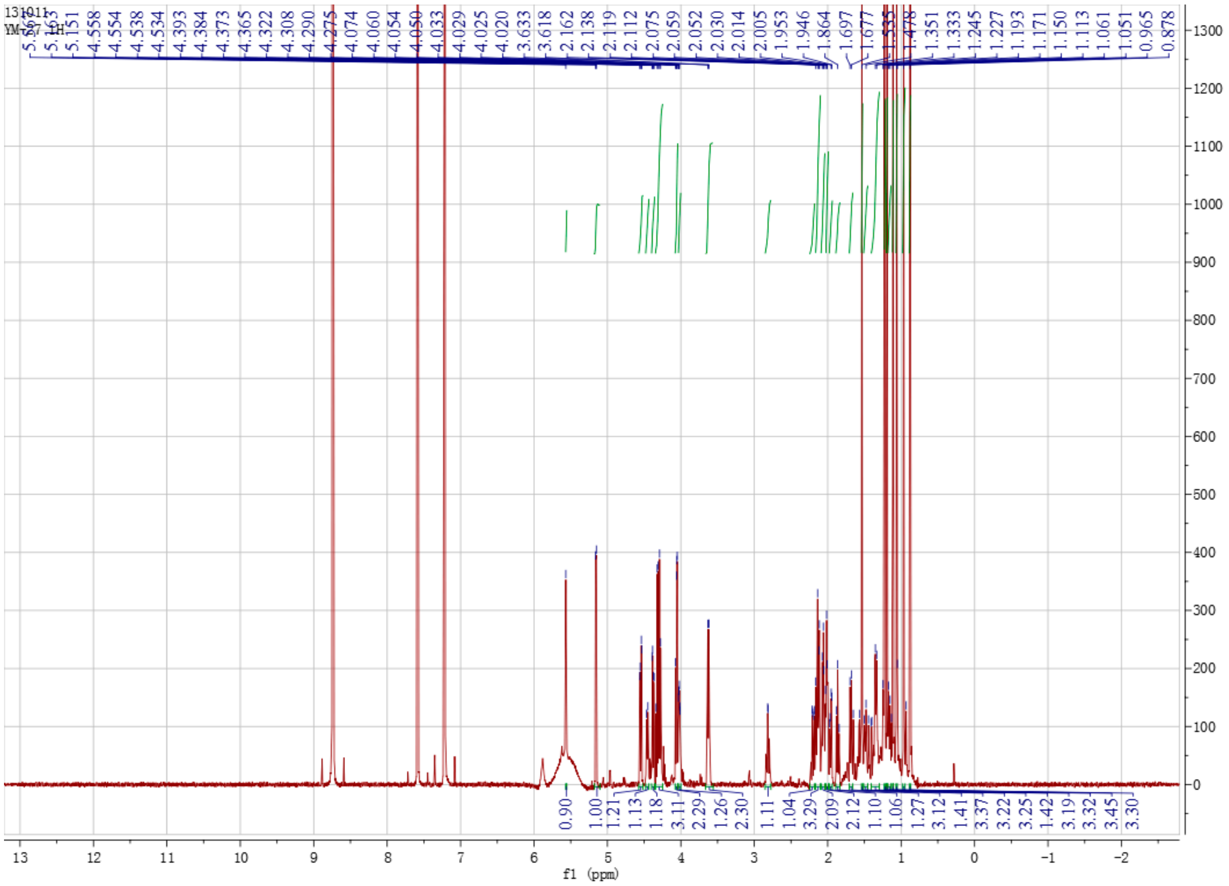

**Figure S2-2.** The Whole $^{1}$H-NMR Spectrum of Compound **2**.

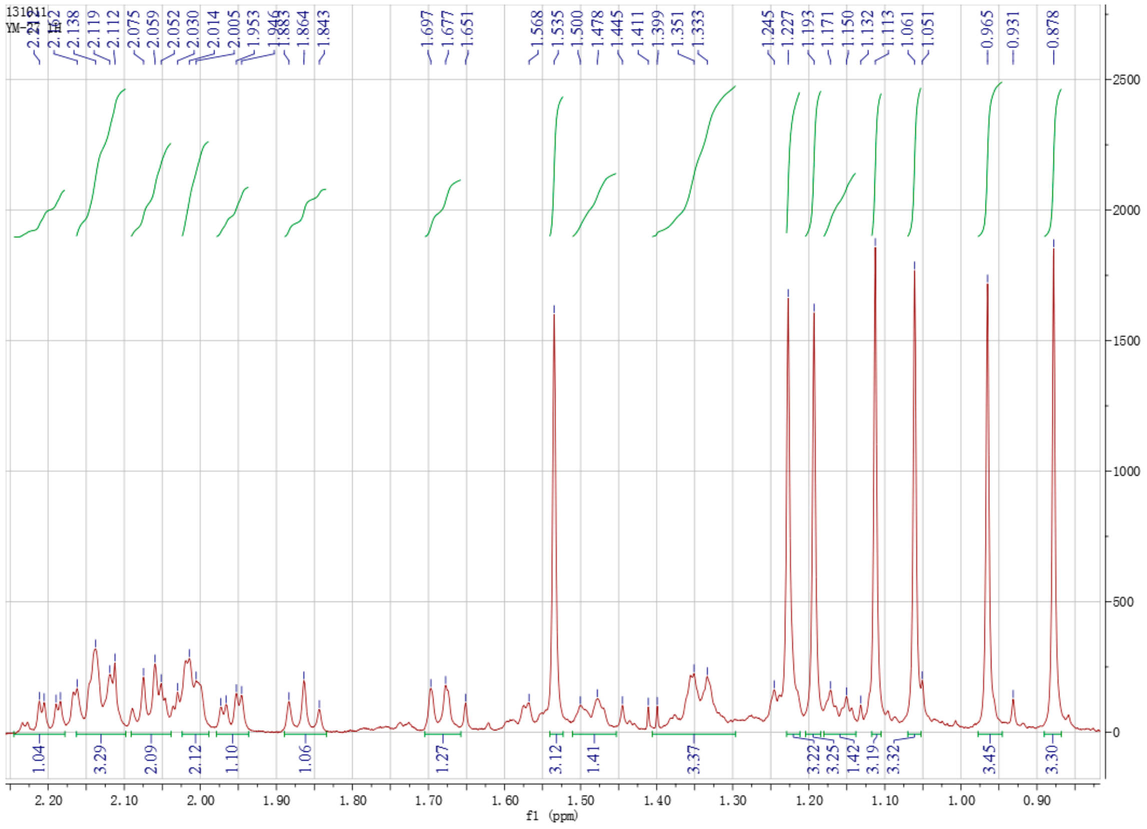

**Figure S2-3.** The Part 1 of the $^{1}$H-NMR Spectrum of Compound **2**.

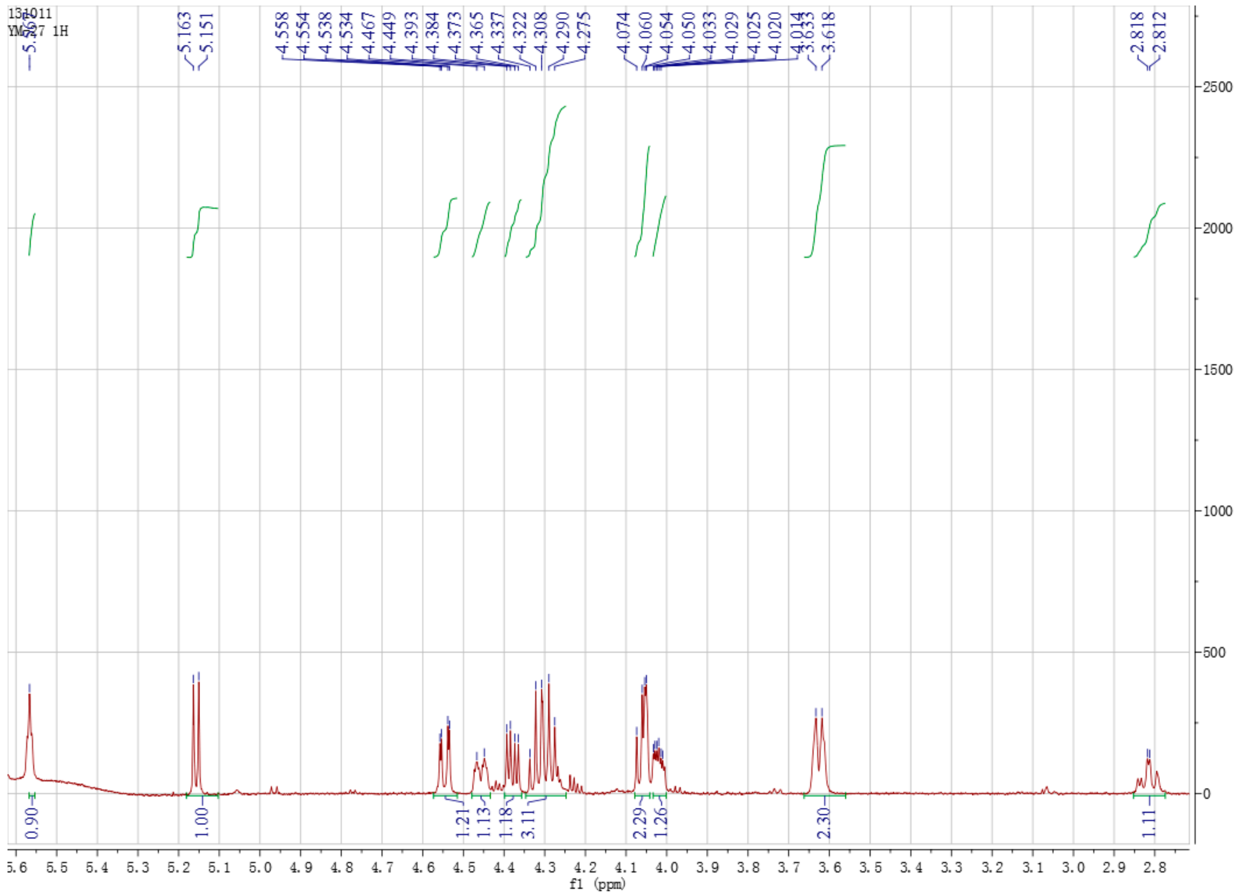

**Figure S2-4.** The Part 1 of the $^{1}$H-NMR Spectrum of Compound **2**.

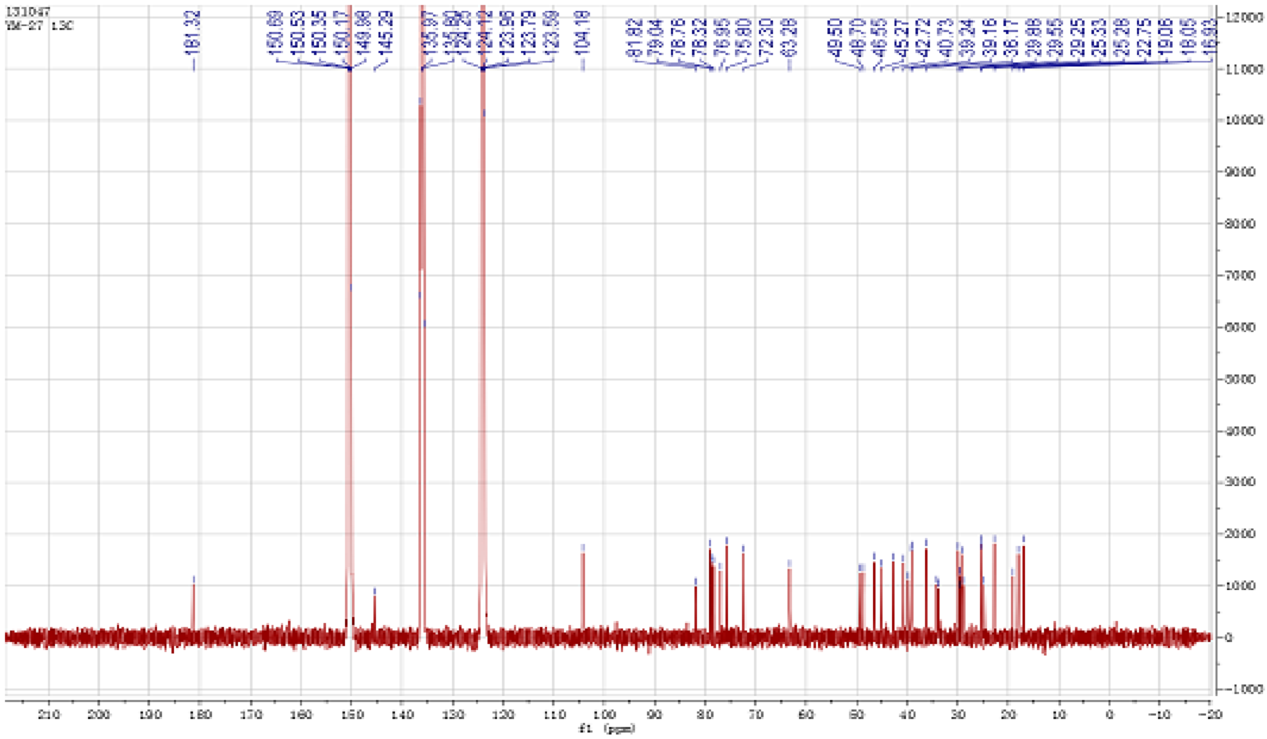

**Figure S2-5.** The $^{13}$C-NMR Spectrum of Compound **2**.

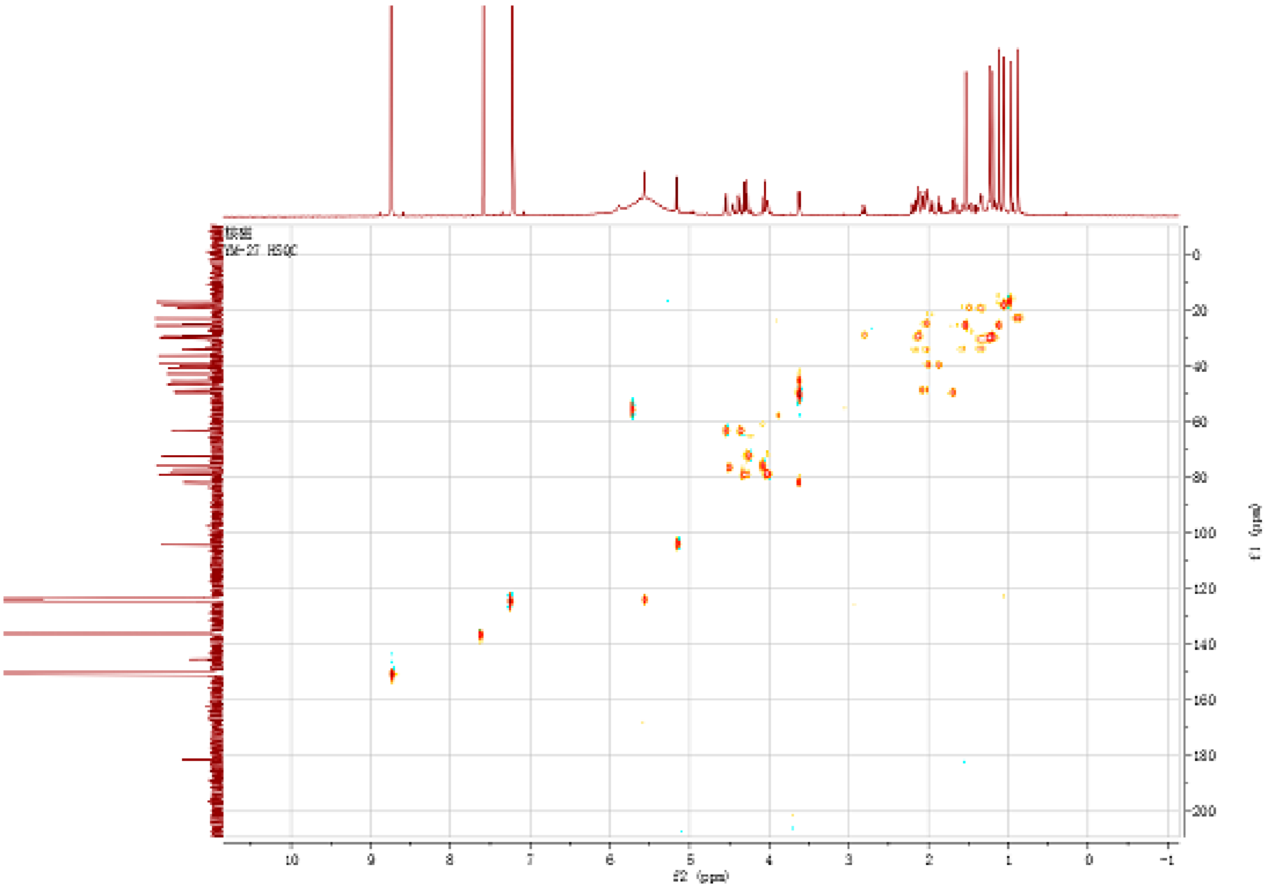


**Figure S2-6.** The Whole HSQC Spectrum of Compound **2**.

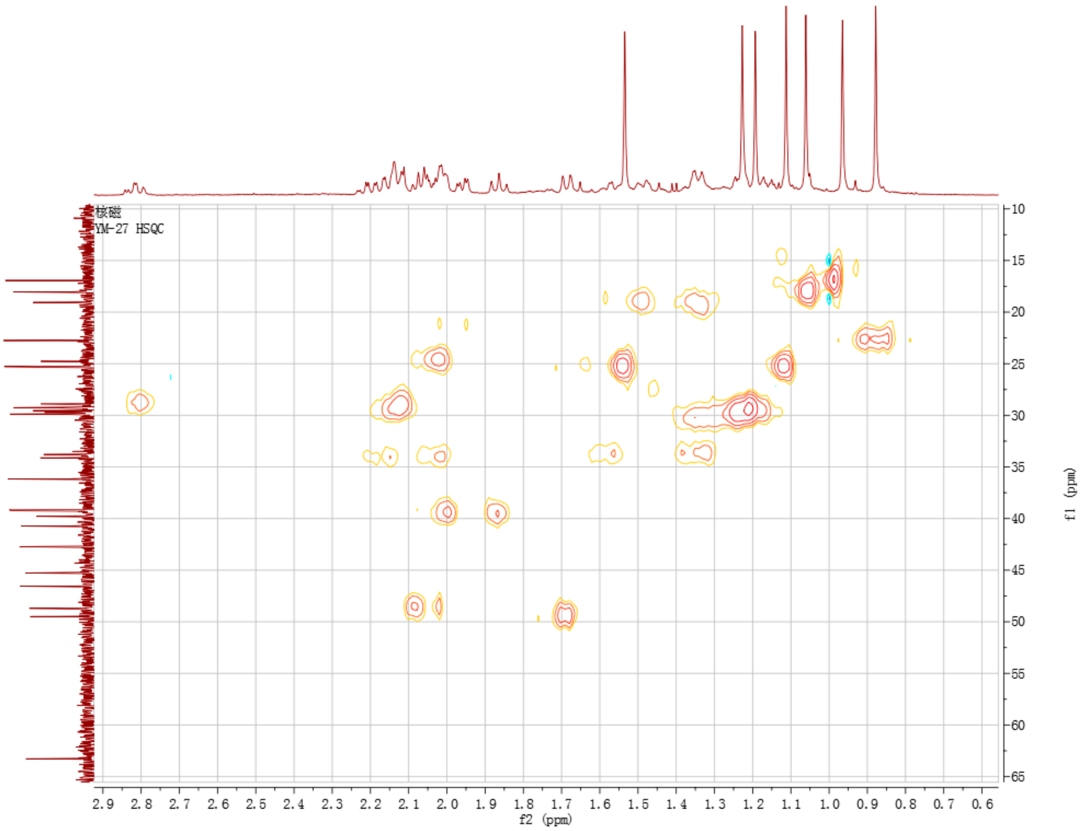


**Figure S2-7.** The Part 1 of the HSQC Spectrum of Compound **2**.

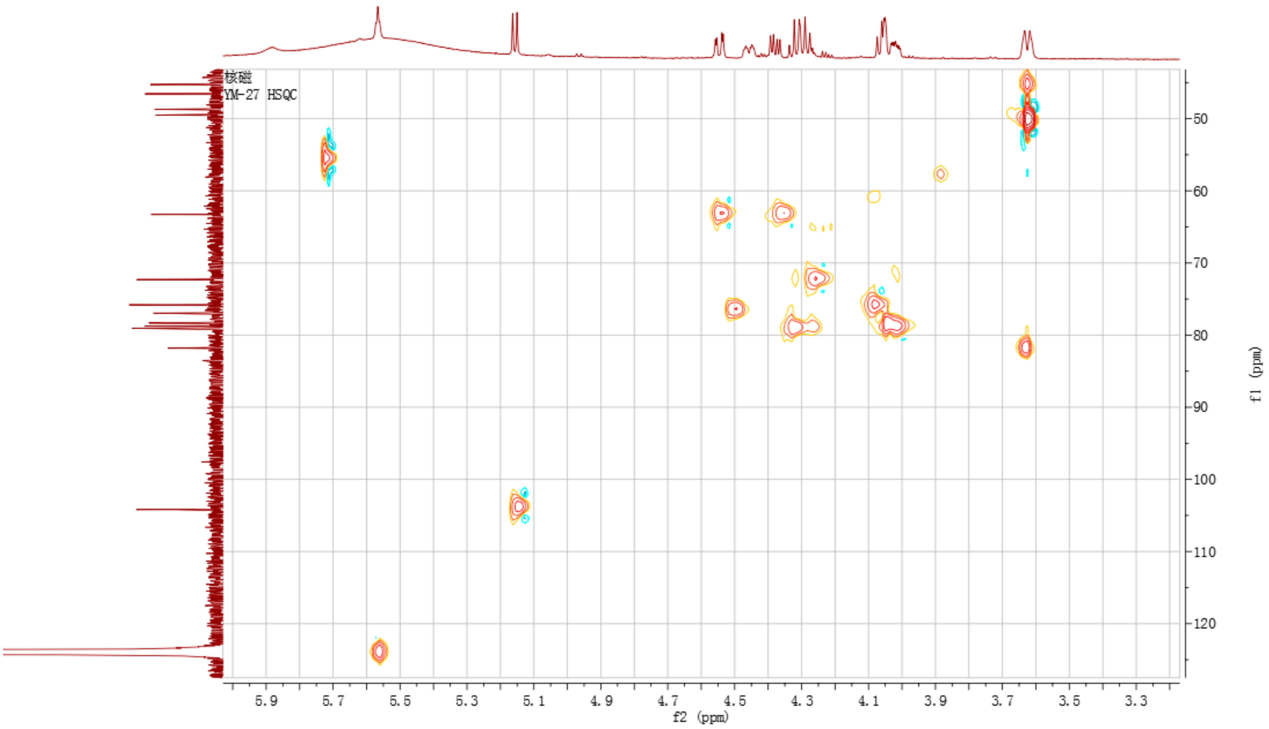


**Figure S2-8.** The Part 2 of the HSQC Spectrum of Compound **2**.

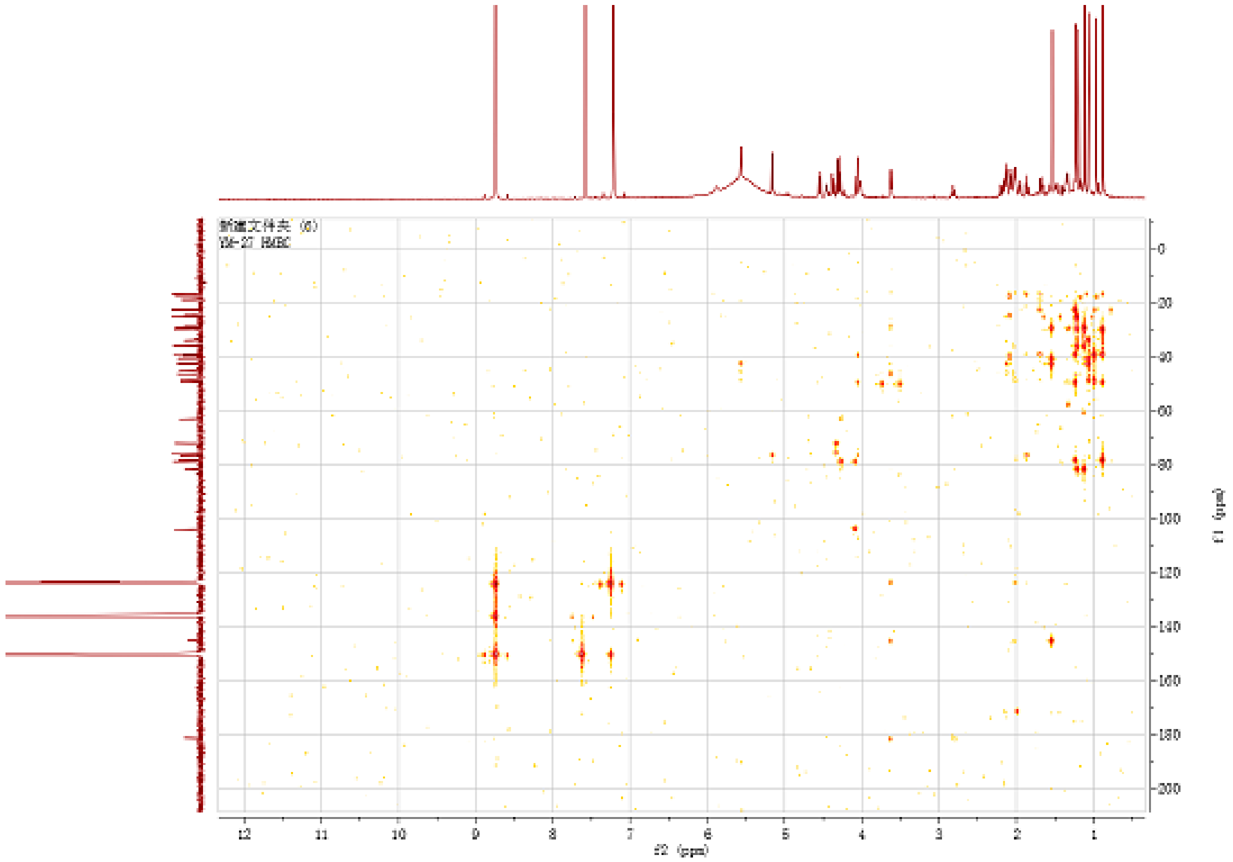


**Figure S2-9.** The Whole HMBC Spectrum of Compound **2**.

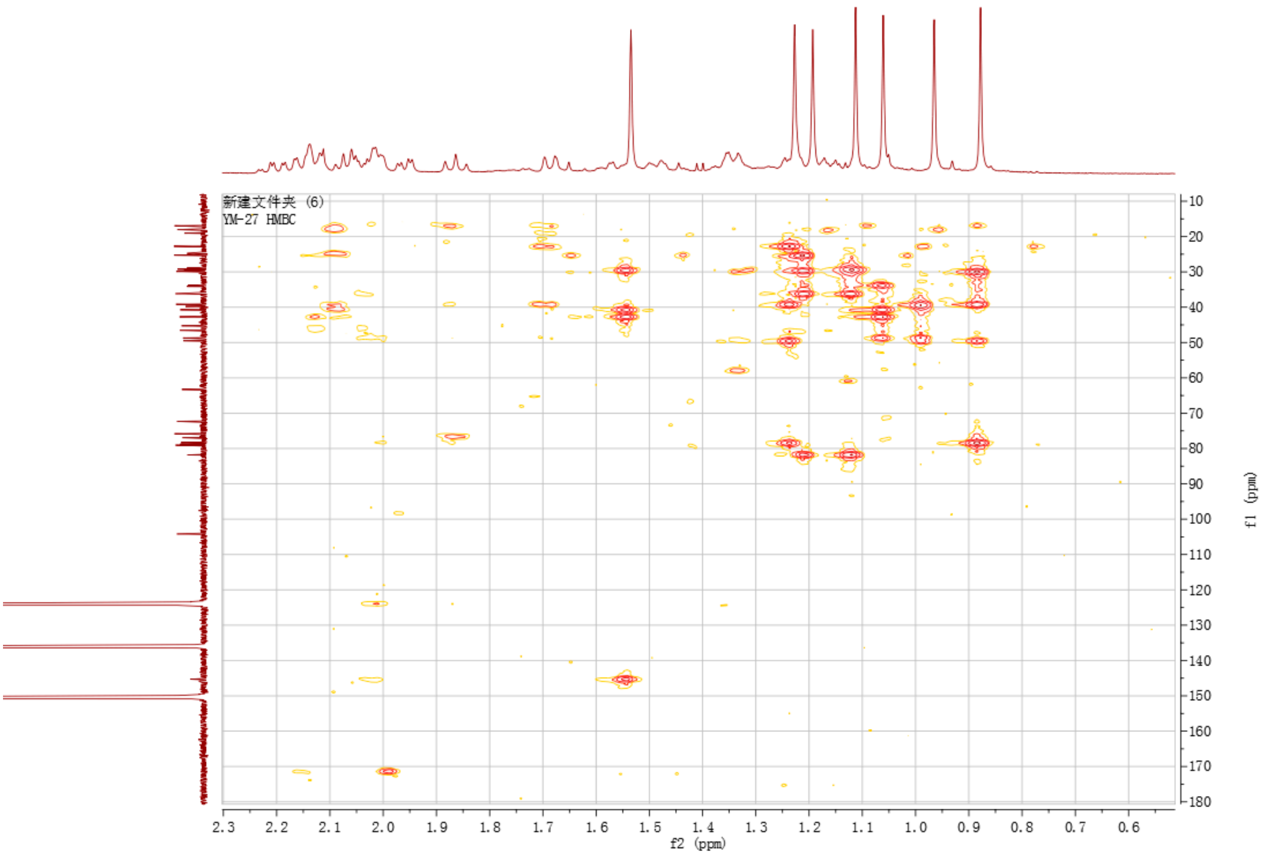


**Figure S2-10.** The Part 1 of the HMBC Spectrum of Compound **2**.

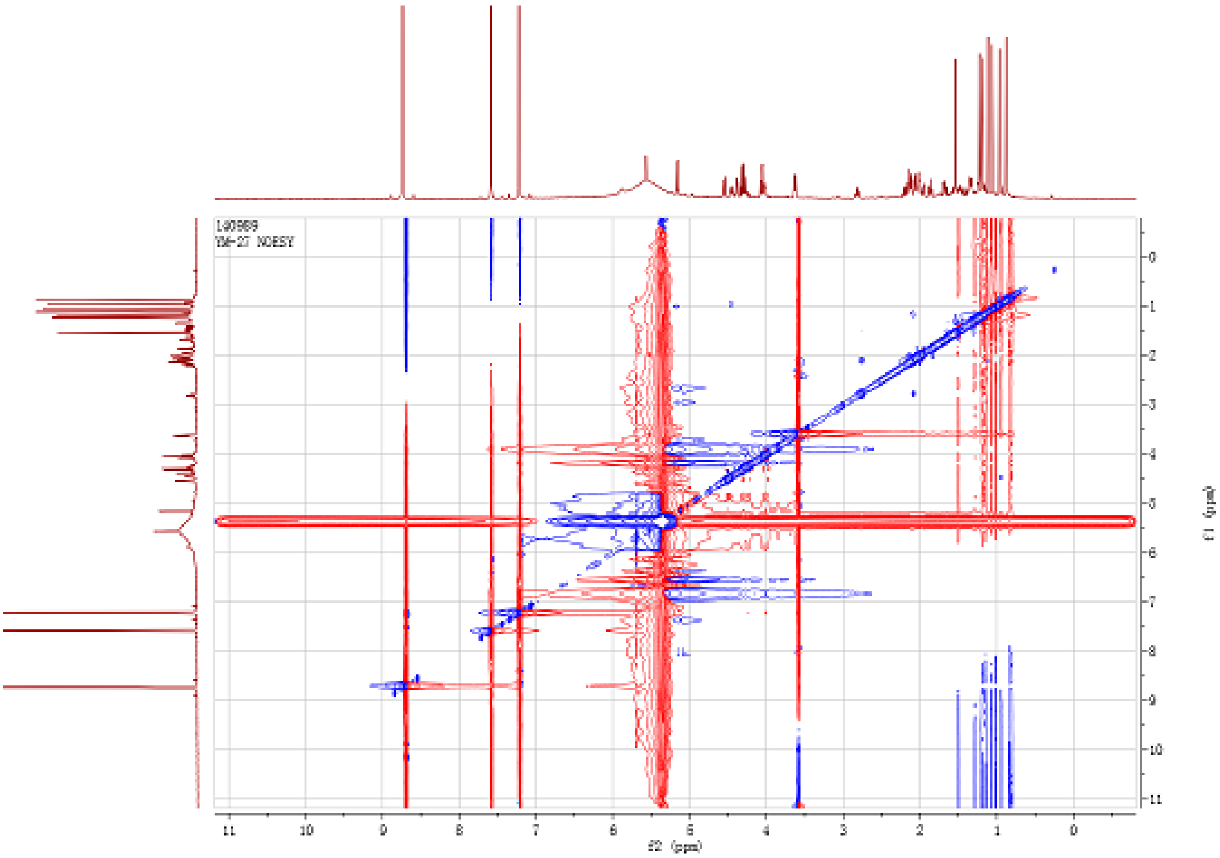


**Figure S2-11.** The Whole NOESY Spectrum of Compound **2**.

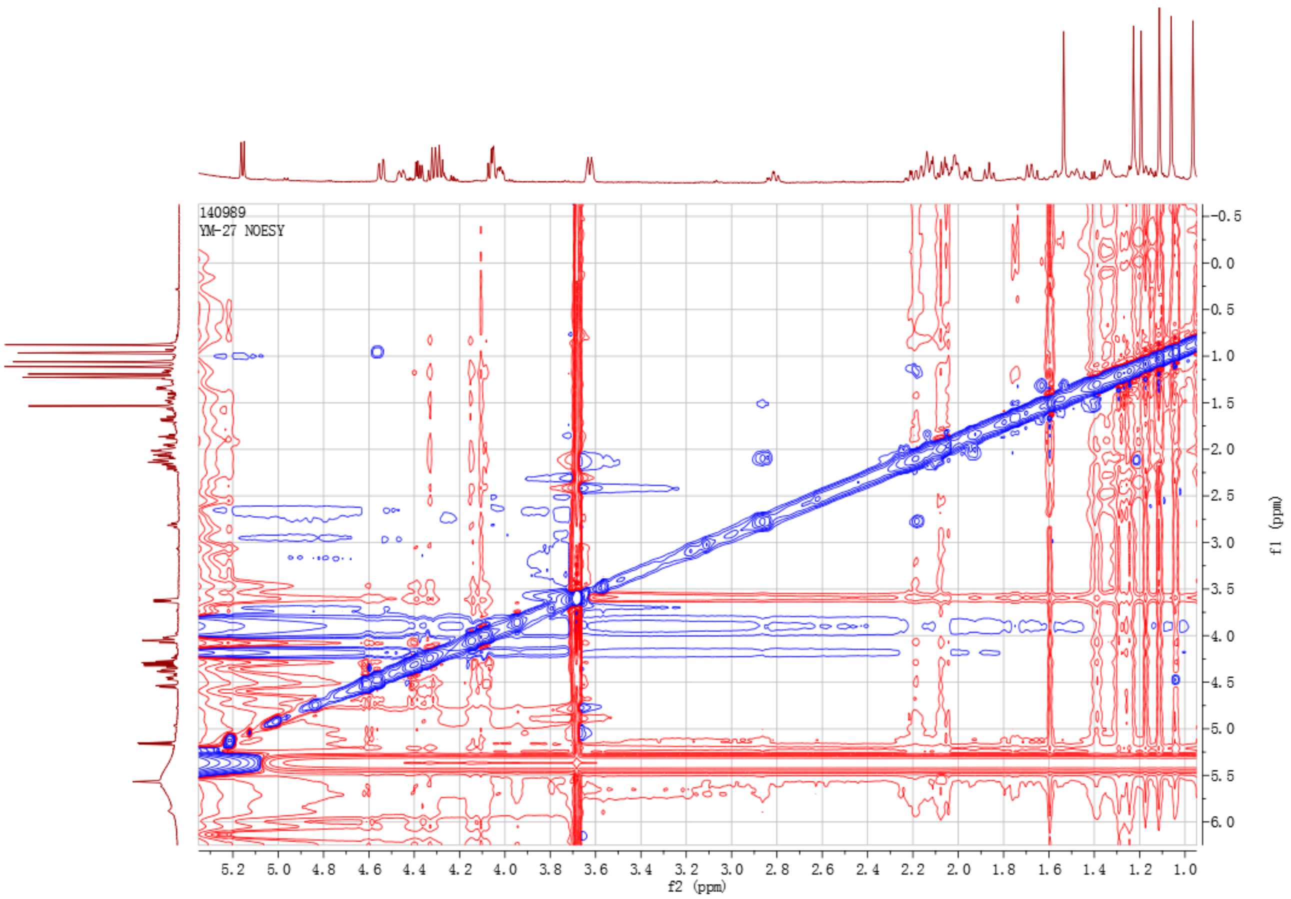


**Figure S2-11.** The Part of the NOESY Spectrum of Compound **2**.

## 3. The Spectrum of Compound 3

# Qualitative Analysis Report

| | | | |
|---|---|---|---|
| Data Filename | 2015041501.d | Sample Name | YM-24 |
| Sample Type | Sample | Position | P1-C1 |
| Instrument Name | Instrument 1 | User Name | |
| Acq Method | | IRM Calibration Status | Success |
| DA Method | TEST LCMS.m | Comment | |

### User Chromatograms

Fragmentor Voltage 120 Collision Energy 0 Ionization Mode ESI

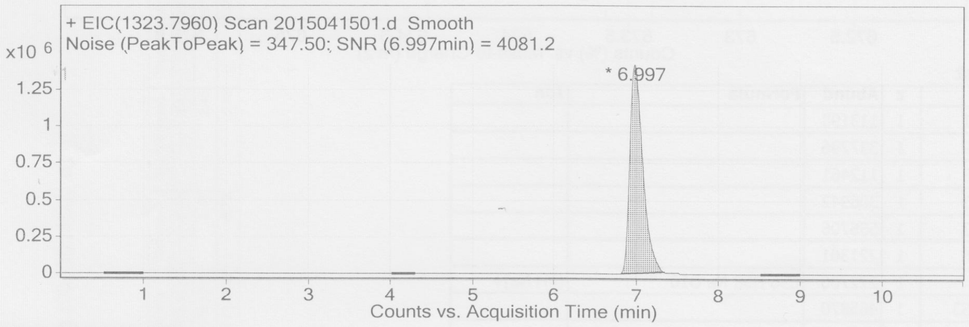


Integration Peak List

| Peak | Start | RT | End | Height | Area | Area % | Signal To Noise |
|---|---|---|---|---|---|---|---|
| 1 | 6.819 | 6.997 | 7.351 | 1418201 | 15579259 | 100 | 4081.2 |

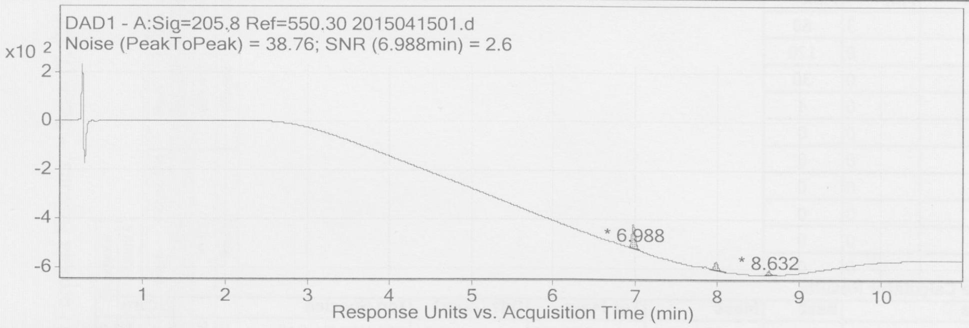


Integration Peak List

| Peak | Start | RT | End | Height | Area | Area % | Signal To Noise |
|---|---|---|---|---|---|---|---|
| 1 | 6.933 | 6.988 | 7.063 | 100.13 | 313.65 | 100 | 2.6 |
| 2 | 7.914 | 7.99 | 8.115 | 36.95 | 124.15 | 39.58 | 1 |
| 3 | 8.577 | 8.632 | 8.731 | 18.52 | 51.25 | 16.34 | 0.5 |

### User Spectra

| Fragmentor Voltage | Collision Energy | Ionization Mode |
|---|---|---|
| 120 | 0 | Esi |

Agilent Technologies Page 1 of 2 Printed at: 11:03 AM on: 4/15/2015

**Figure S3-1.** The Part 1 of the HR-ESI-MS Spectrum of Compound **3**.

## Qualitative Analysis Report

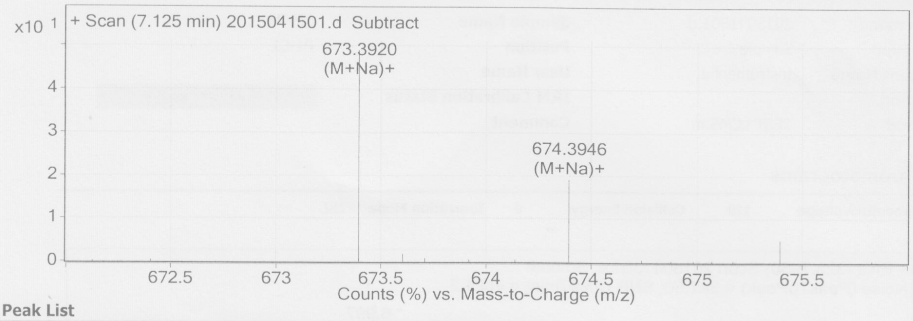


**Peak List**

| m/z | z | Abund | Formula | Ion |
|---|---|---|---|---|
| 453.3361 | 1 | 113198 | | |
| 471.3469 | 1 | 337296 | | |
| 472.3498 | 1 | 112461 | | |
| 489.3573 | 1 | 306942 | | |
| 668.4373 | 1 | 556706 | | |
| 669.4396 | 1 | 221301 | | |
| 673.392 | 1 | 272700 | C36 H58 Na O10 | (M+Na)+ |
| 1323.7953 | 1 | 465870 | | |
| 1324.7992 | 1 | 376913 | | |
| 1325.8003 | 1 | 170143 | | |

**Formula Calculator Element Limits**

| Element | Min | Max |
|---|---|---|
| C | 3 | 60 |
| H | 0 | 120 |
| O | 0 | 30 |
| N | 0 | 4 |
| S | 0 | 0 |
| Cl | 0 | 0 |
| P | 0 | 0 |
| Br | 0 | 0 |
| F | 0 | 0 |
| Si | 0 | 0 |

**Formula Calculator Results**

| Formula | Best | Mass | Tgt Mass | Diff (ppm) | Ion Species | Score |
|---|---|---|---|---|---|---|
| C36 H58 O10 | TRUE | 650.4027 | 650.403 | 0.41 | C36 H58 Na O10 | 99.9 |
| C37 H54 N4 O6 | | 650.4027 | 650.4043 | 2.45 | C37 H54 N4 Na O6 | 99.63 |
| C31 H58 N2 O12 | | 650.4027 | 650.399 | -5.78 | C31 H58 N2 Na O12 | 99.23 |

--- End Of Report ---

Agilent Technologies Page 2 of 2 Printed at: 11:03 AM on: 4/15/2015

**Figure S3-2.** The Part 2 of the HR-ESI-MS Spectrum of Compound **3**.

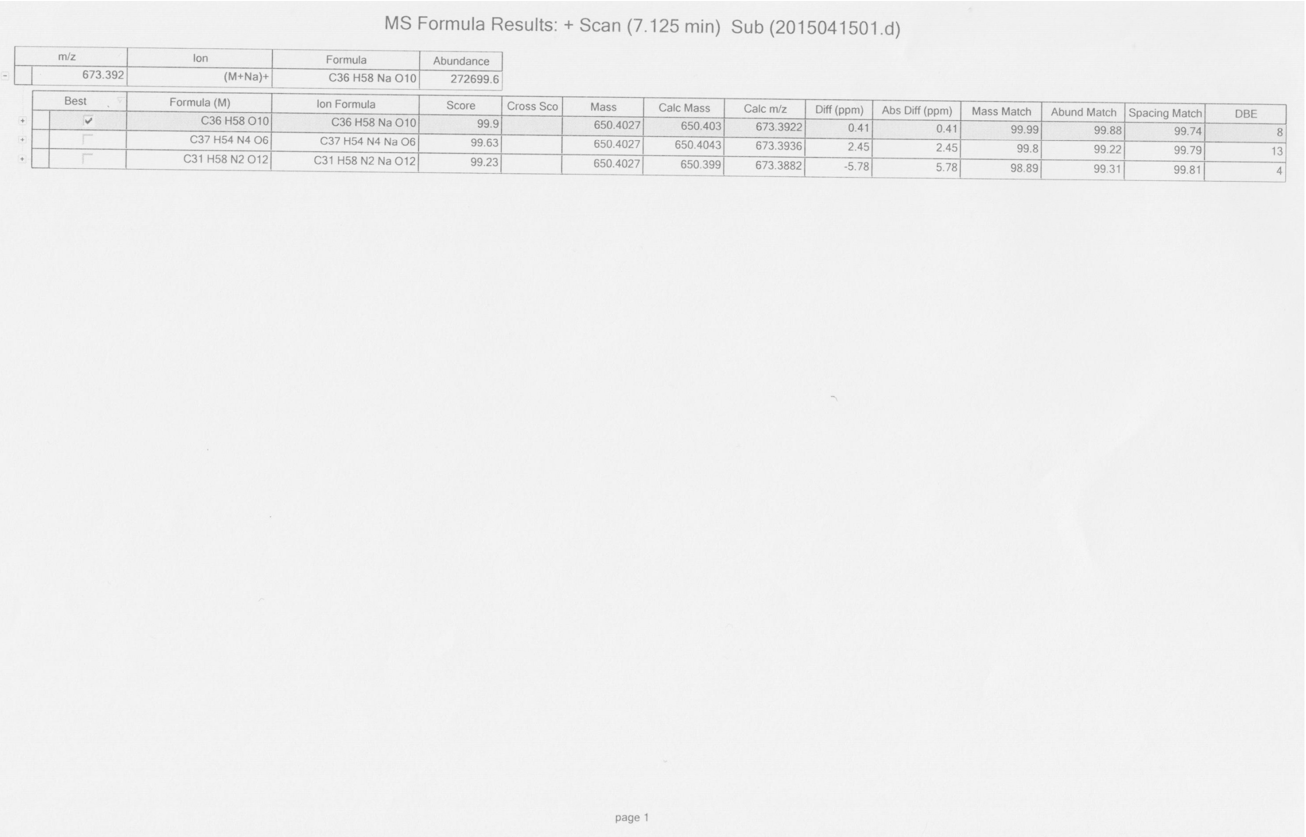

MS Formula Results: + Scan (7.125 min) Sub (2015041501.d)

| m/z | Ion | Formula | Abundance |
|---|---|---|---|
| 673.392 | (M+Na)+ | C36 H58 Na O10 | 272699.6 |

| Best | Formula (M) | Ion Formula | Score | Cross Sco | Mass | Calc Mass | Calc m/z | Diff (ppm) | Abs Diff (ppm) | Mass Match | Abund Match | Spacing Match | DBE |
|---|---|---|---|---|---|---|---|---|---|---|---|---|---|
| ✓ | C36 H58 O10 | C36 H58 Na O10 | 99.9 | | 650.4027 | 650.403 | 673.3922 | 0.41 | 0.41 | 99.99 | 99.88 | 99.74 | 8 |
| | C37 H54 N4 O6 | C37 H54 N4 Na O6 | 99.63 | | 650.4027 | 650.4043 | 673.3936 | 2.45 | 2.45 | 99.8 | 99.22 | 99.79 | 13 |
| | C31 H58 N2 O12 | C31 H58 N2 Na O12 | 99.23 | | 650.4027 | 650.399 | 673.3882 | -5.78 | 5.78 | 98.89 | 99.31 | 99.81 | 4 |

page 1

**Figure S3-3.** The Part 3 of the HR-ESI-MS Spectrum of Compound **3**.

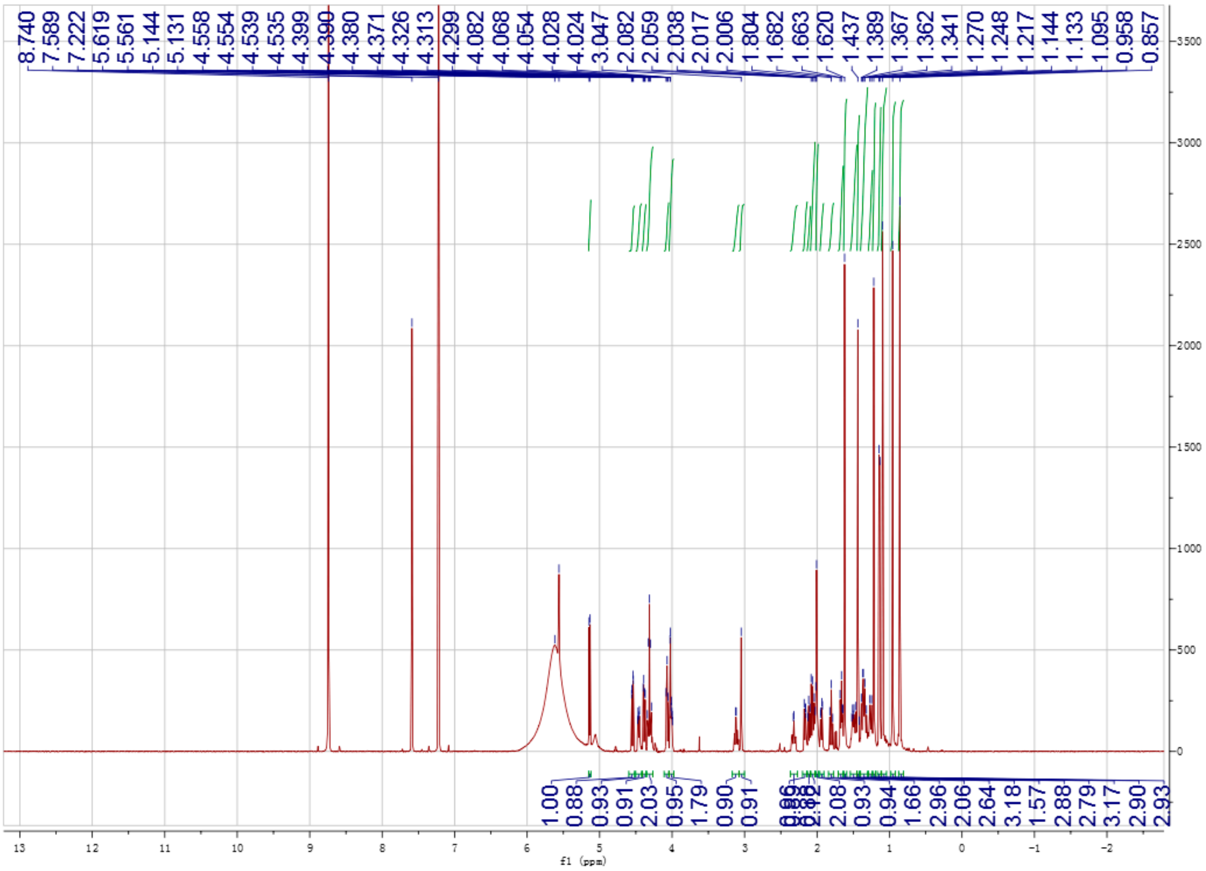


**Figure S3-4.** The Whole $^1$H-NMR Spectrum of Compound **3**.

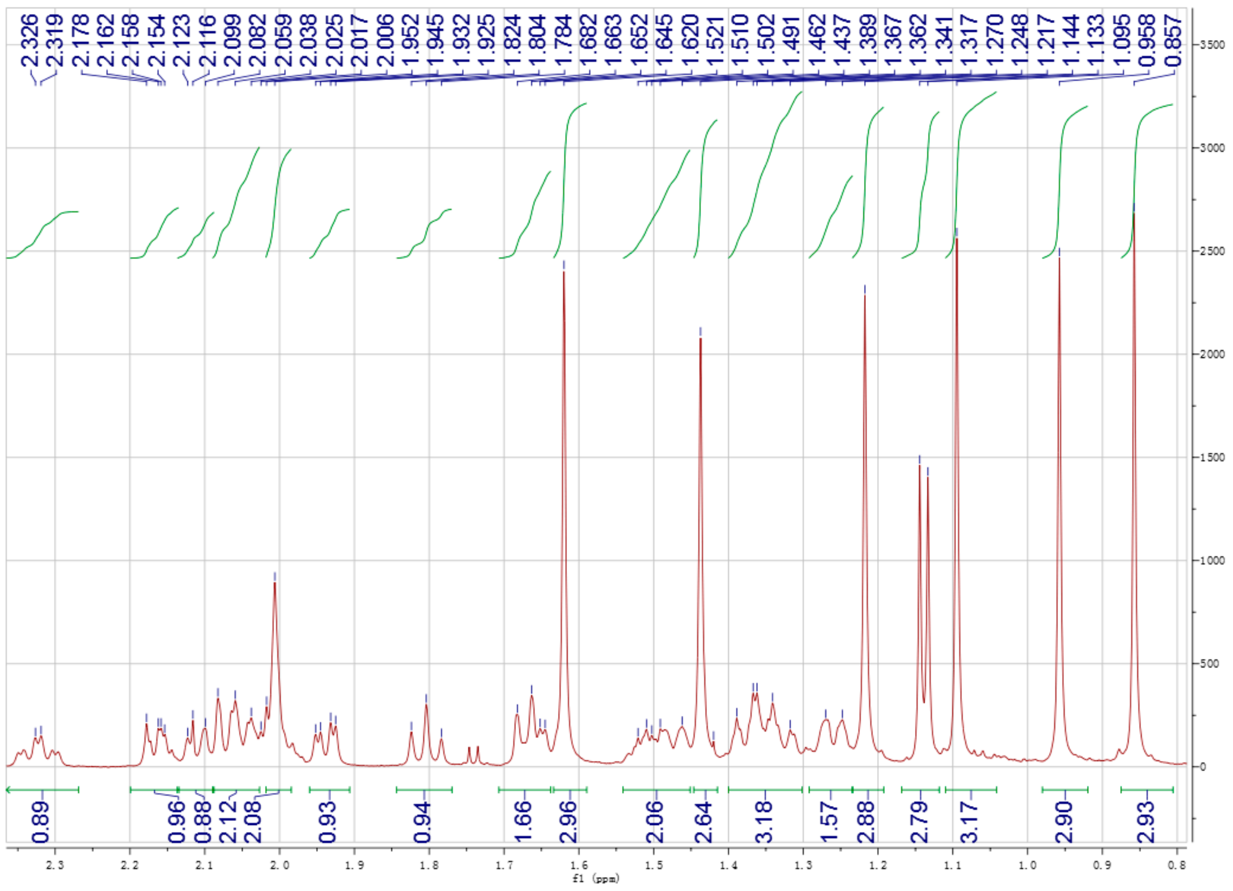

**Figure S3-5.** The Part 1 of the $^{1}$H-NMR Spectrum of Compound **3**.

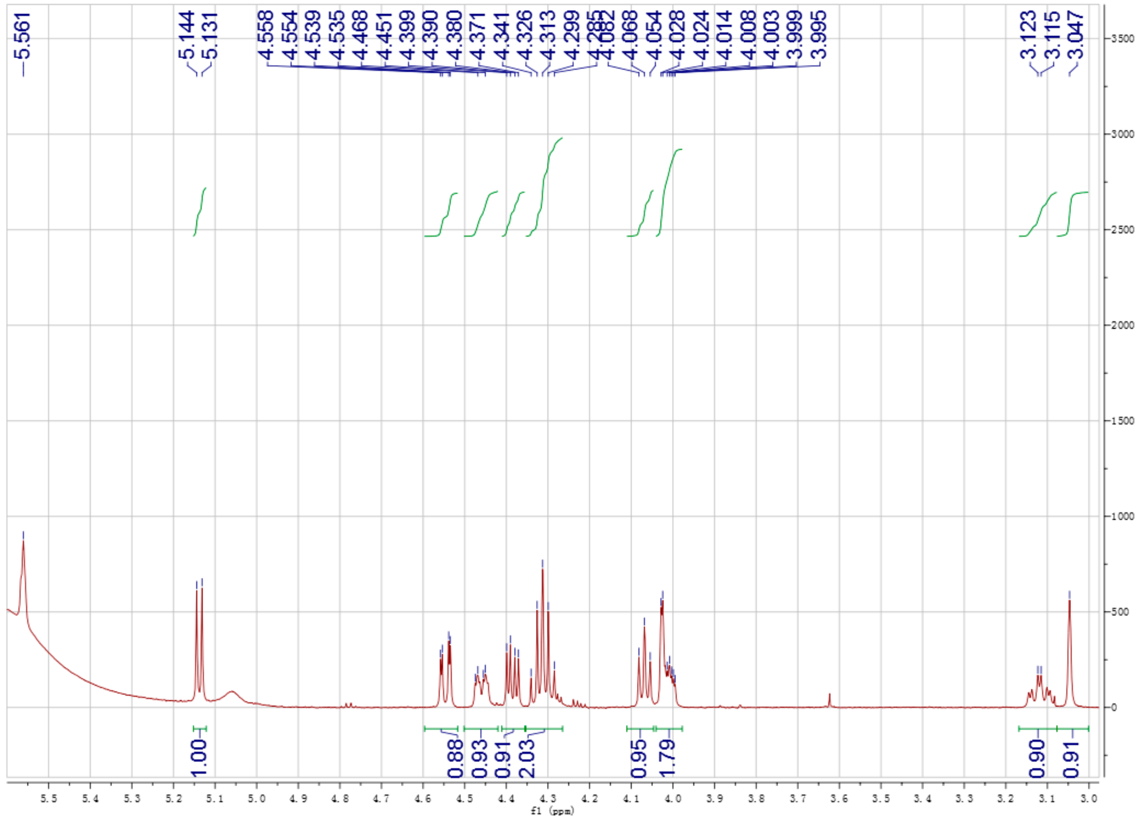

**Figure S3-6.** The Part 2 of the $^{1}$H-NMR Spectrum of Compound **3**.

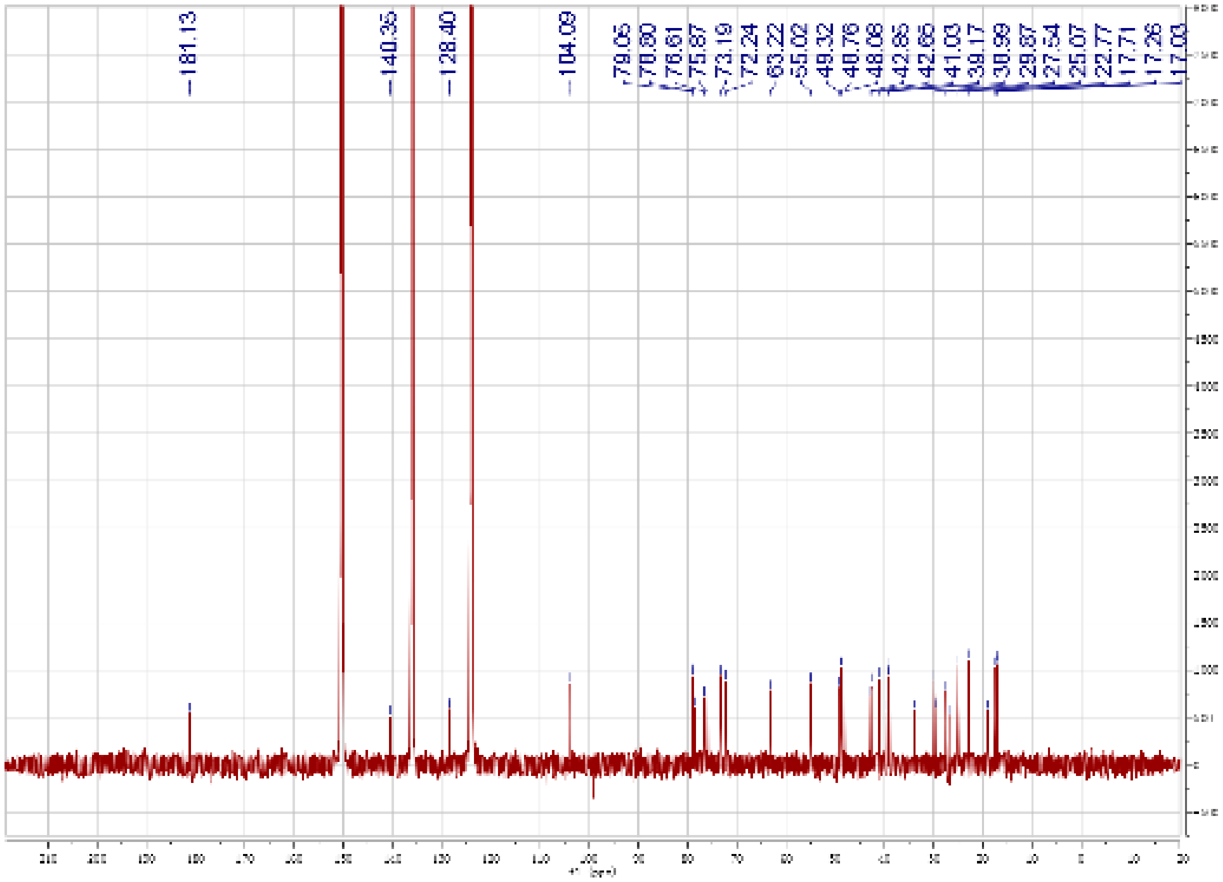


**Figure S3-7.** The $^{13}$C-NMR Spectrum of Compound **3**.

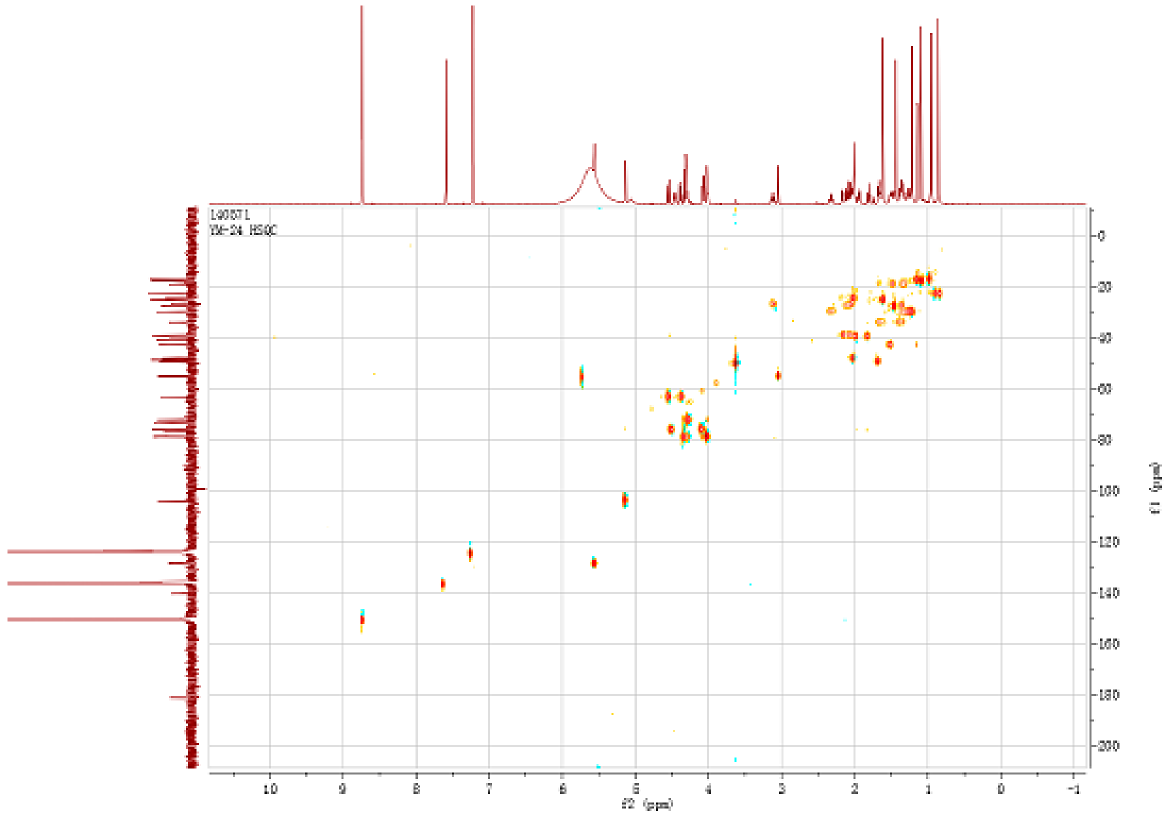

**Figure S3-8.** The Whole HSQC Spectrum of Compound **3.**

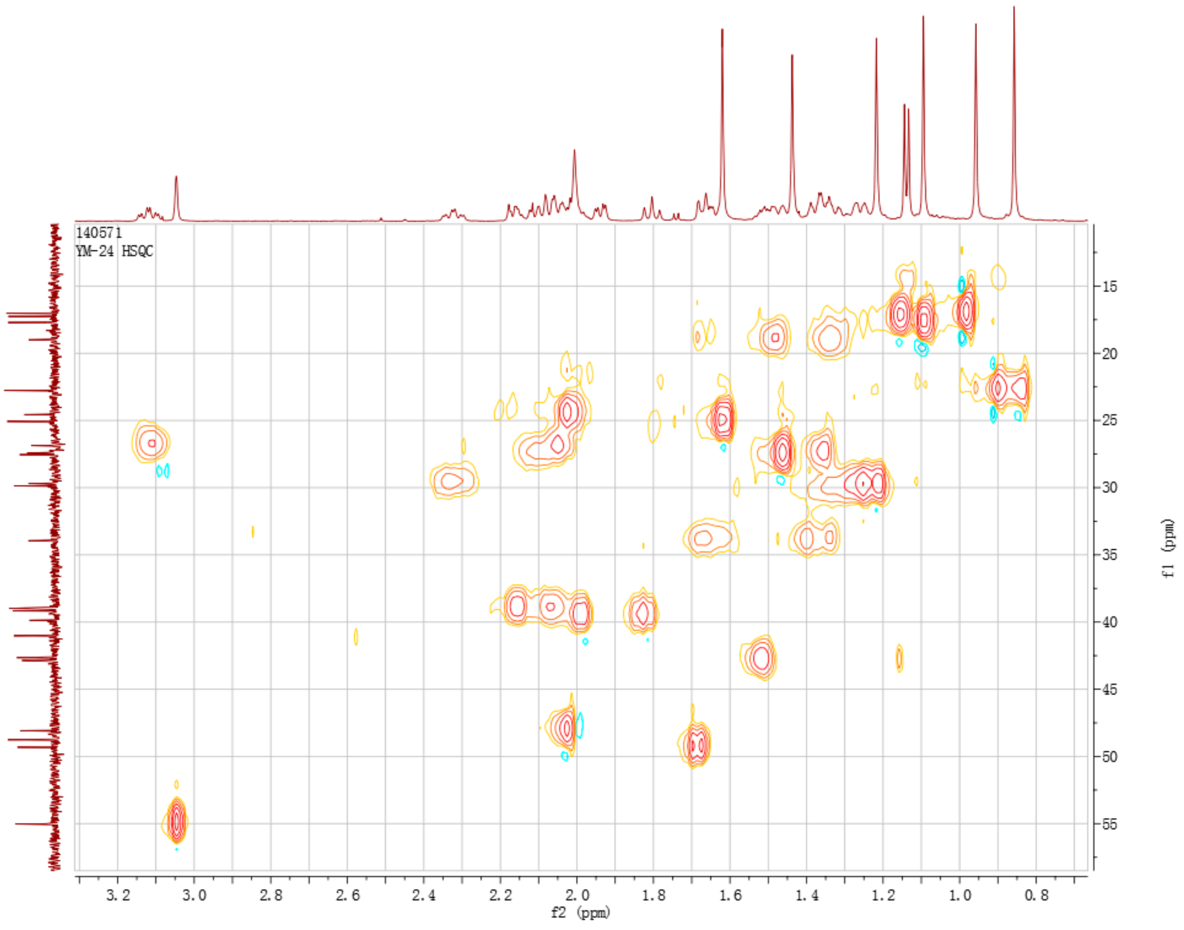


**Figure S3-9.** The Part 1 of the HSQC Spectrum of Compound **3**.

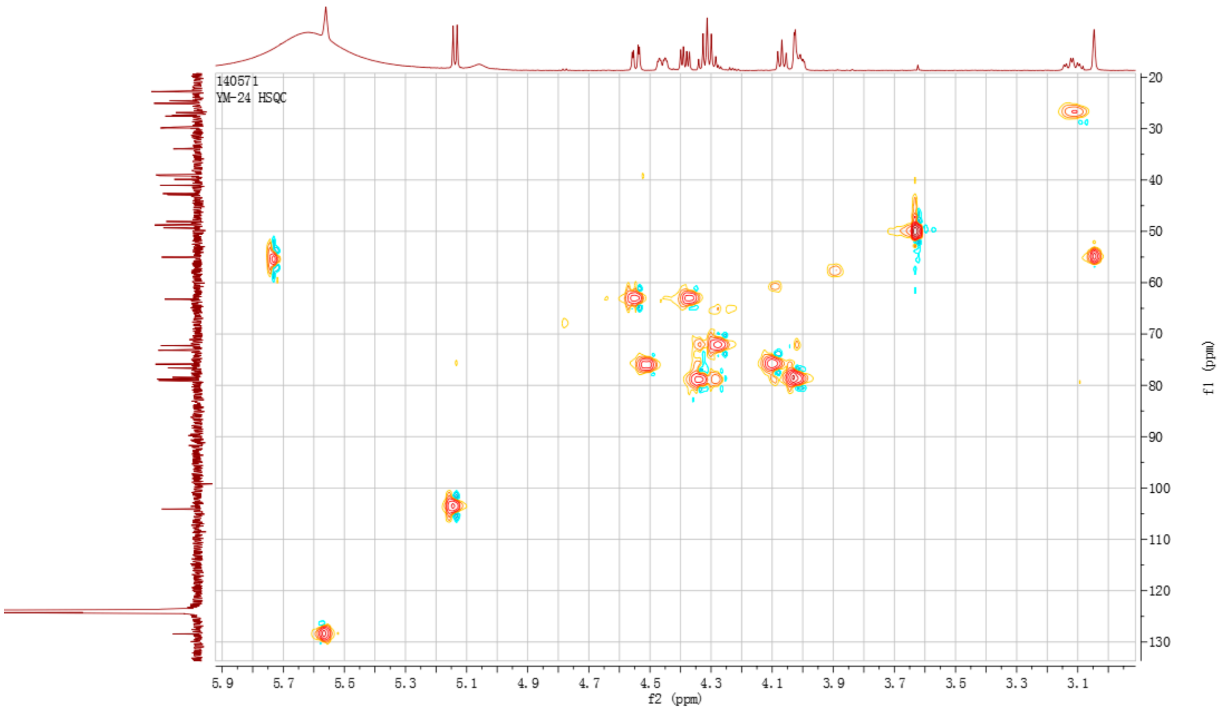


**Figure S3-10.** The Part 2 of the HSQC Spectrum of Compound **3**.

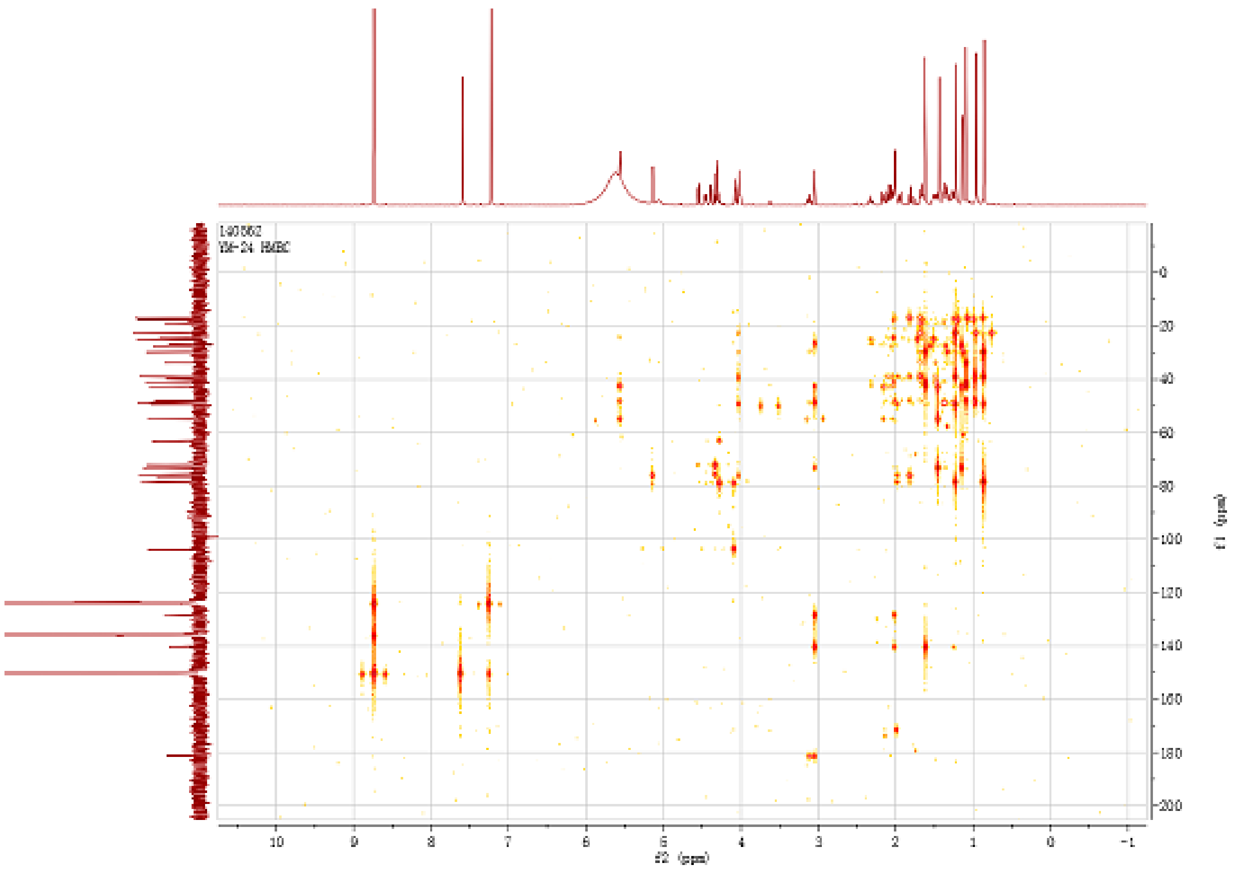


**Figure S3-11.** The Whole HMBC Spectrum of Compound **3**.

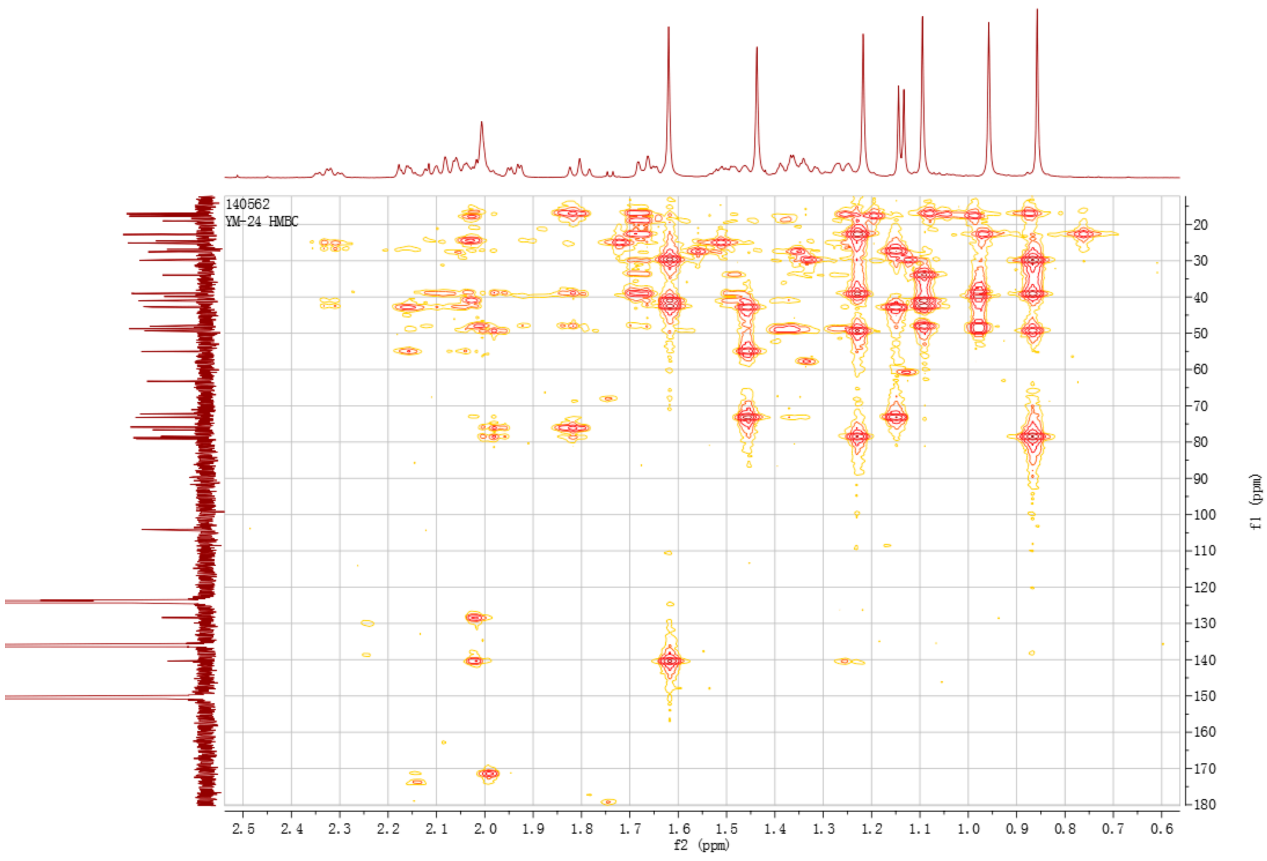


**Figure S3-12.** The Part 1 of the HMBC Spectrum of Compound **3**.

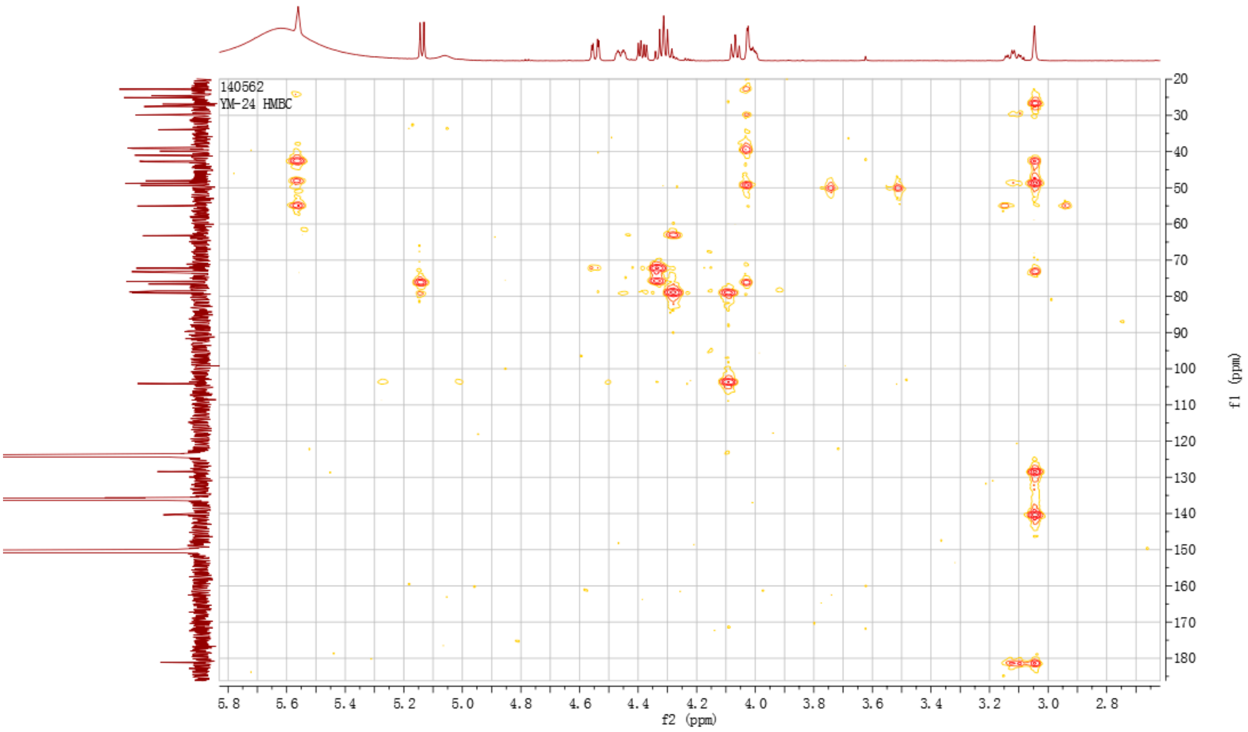


**Figure S3-13.** The Part 2 of the HMBC Spectrum of Compound **3**.

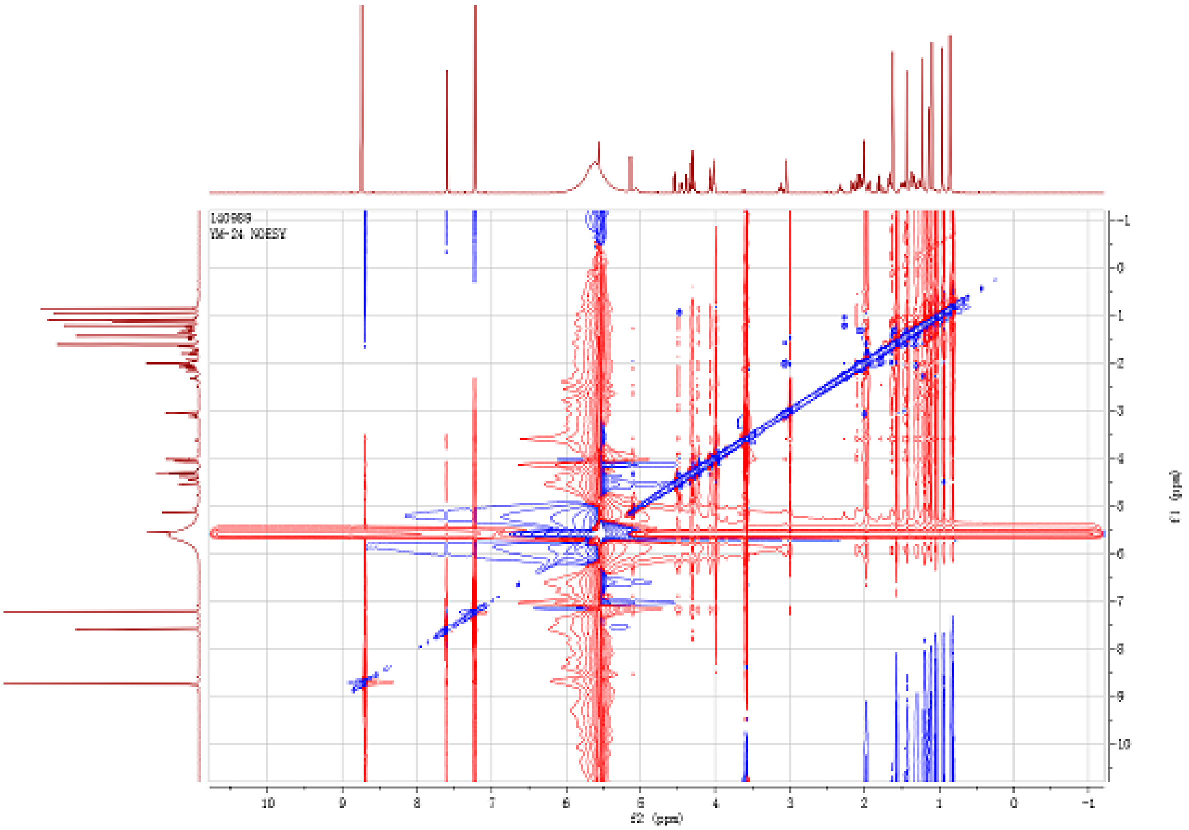


**Figure S3-14.** The NOESY Spectrum of Compound **3**.

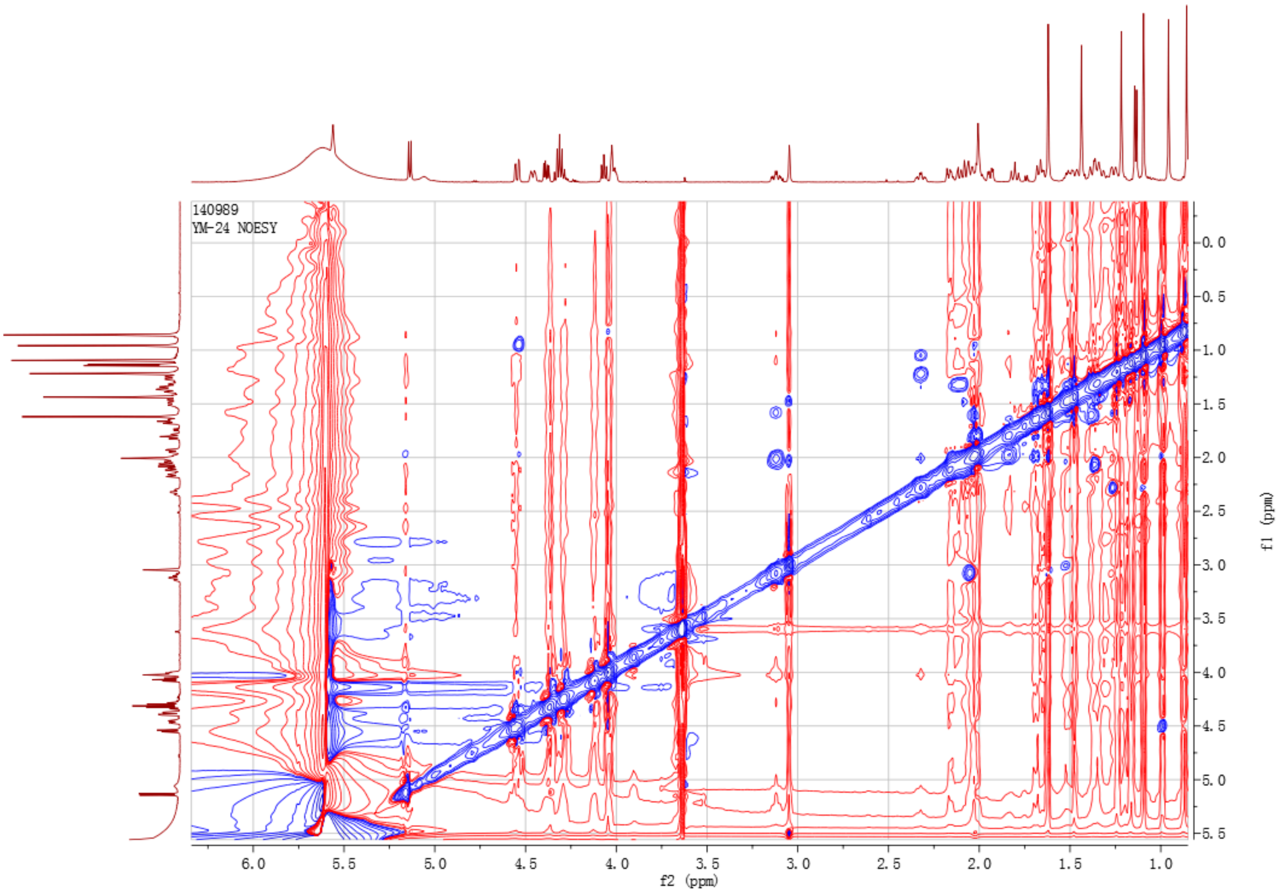


**Figure S3-15.** The Part of the NOESY Spectrum of Compound **3**.

## 4. The Spectrum of Compound 4

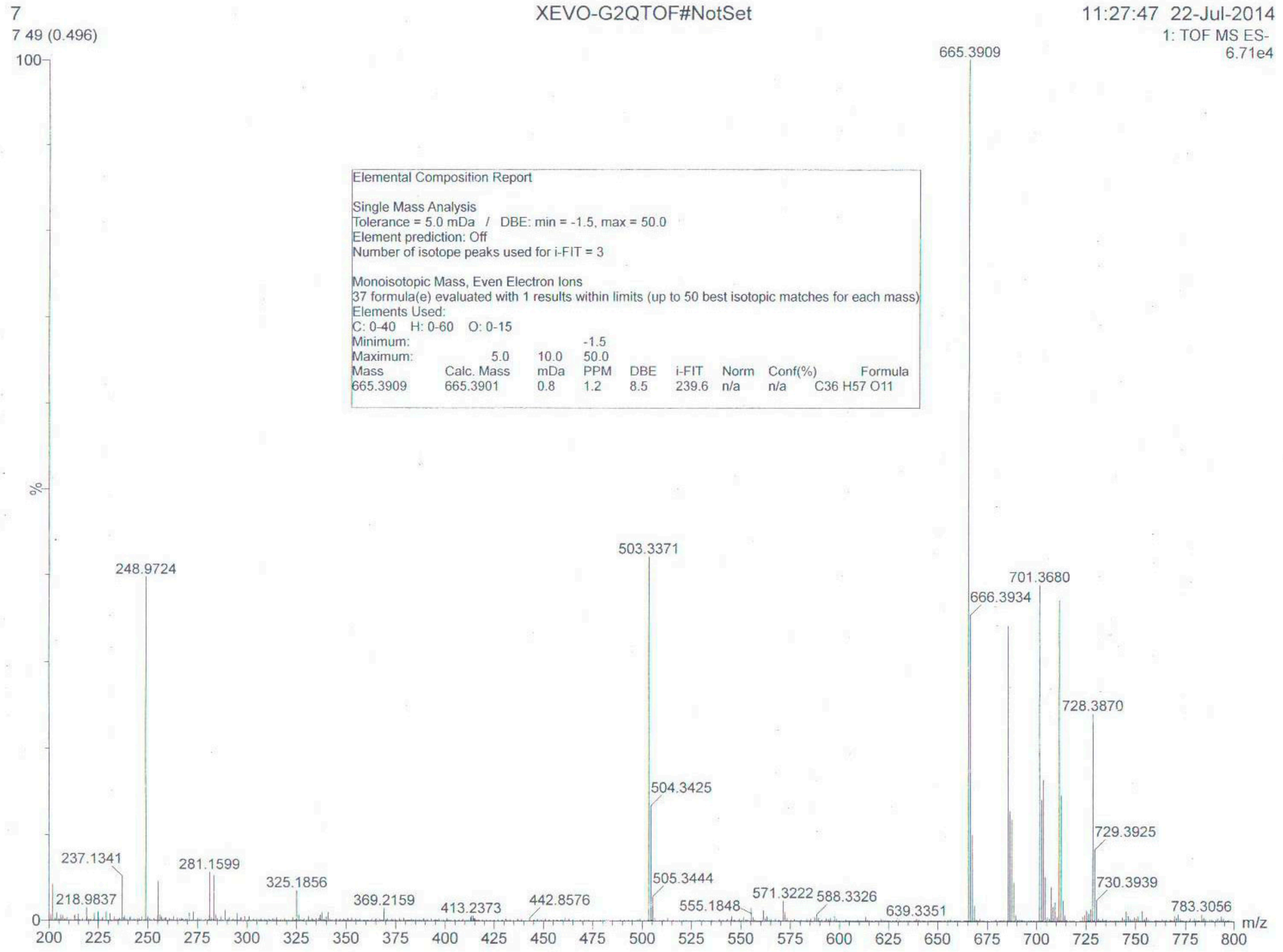


**Figure S4-1.** The HR-ESI-MS Spectrum of Compound **4**.

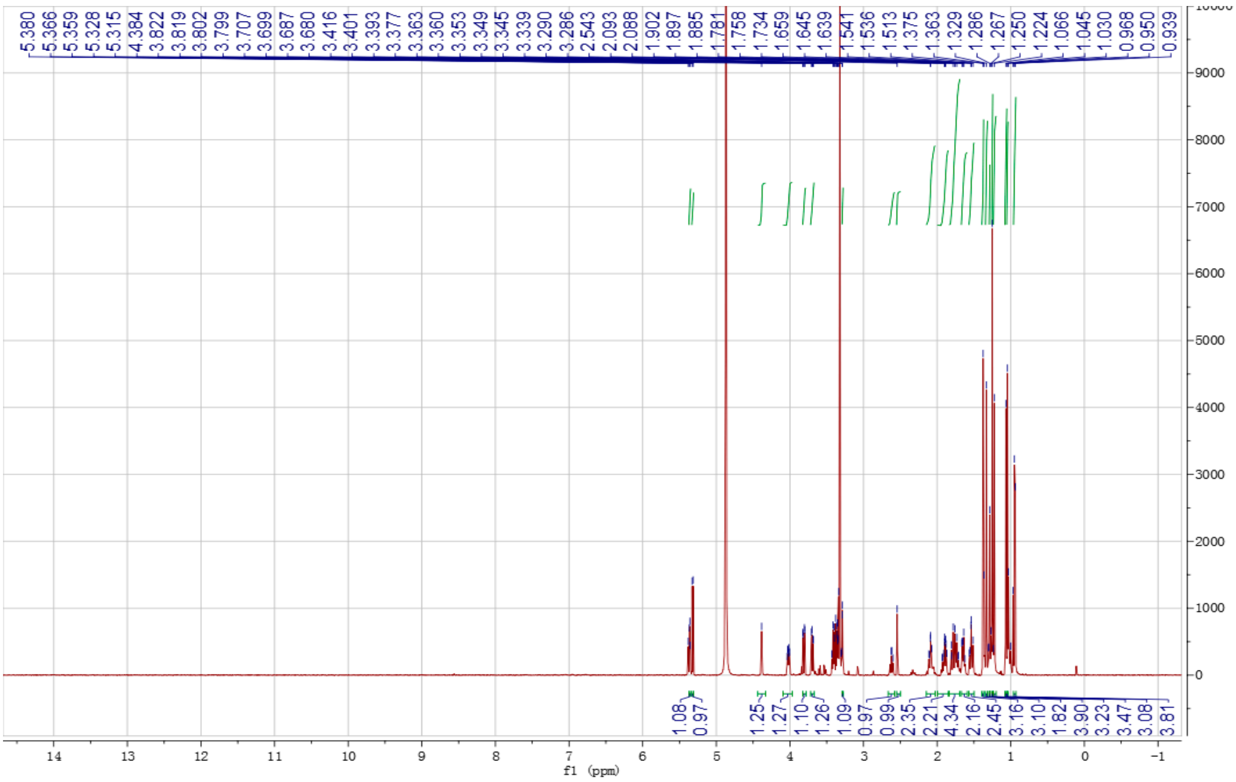


**Figure S4-2.** The Whole $^{1}$H-NMR Spectrum of Compound **4**.

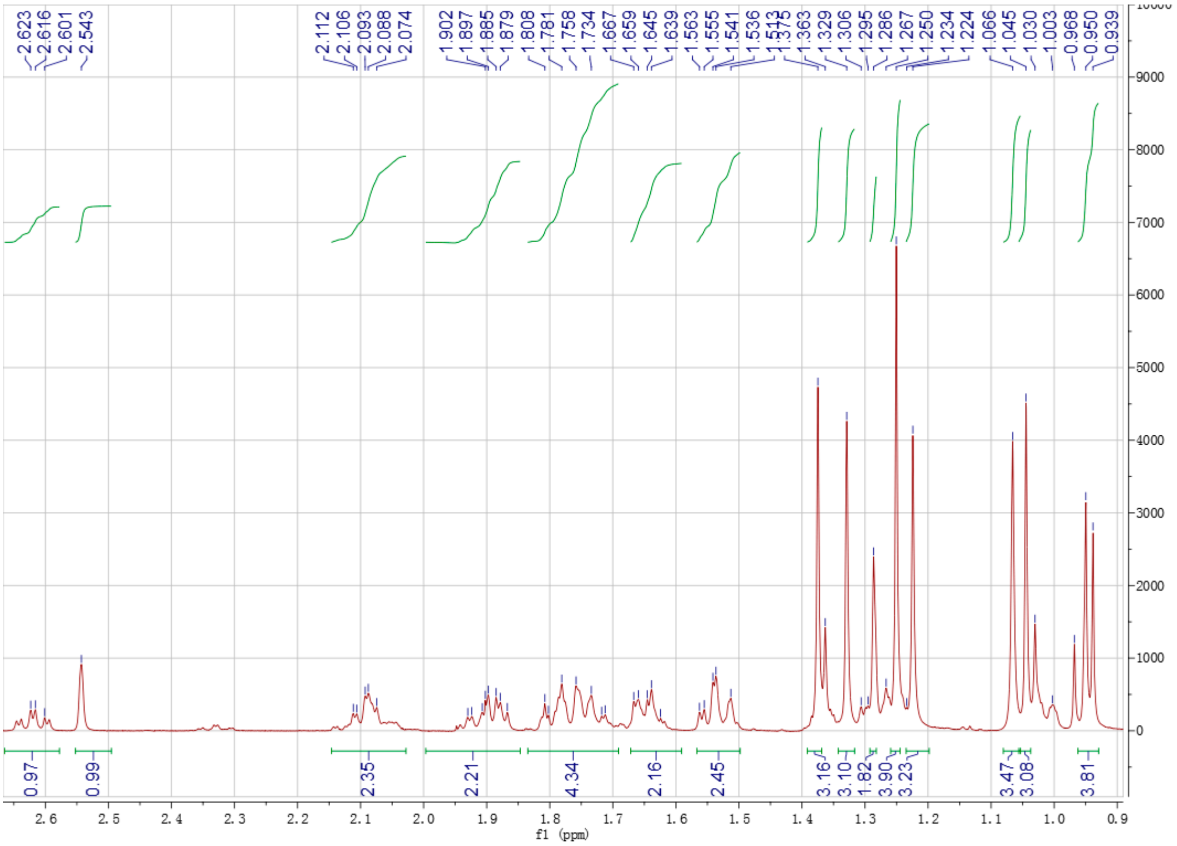


**Figure S4-3.** The Part 1 of the $^{1}$H-NMR Spectrum of Compound **4**.

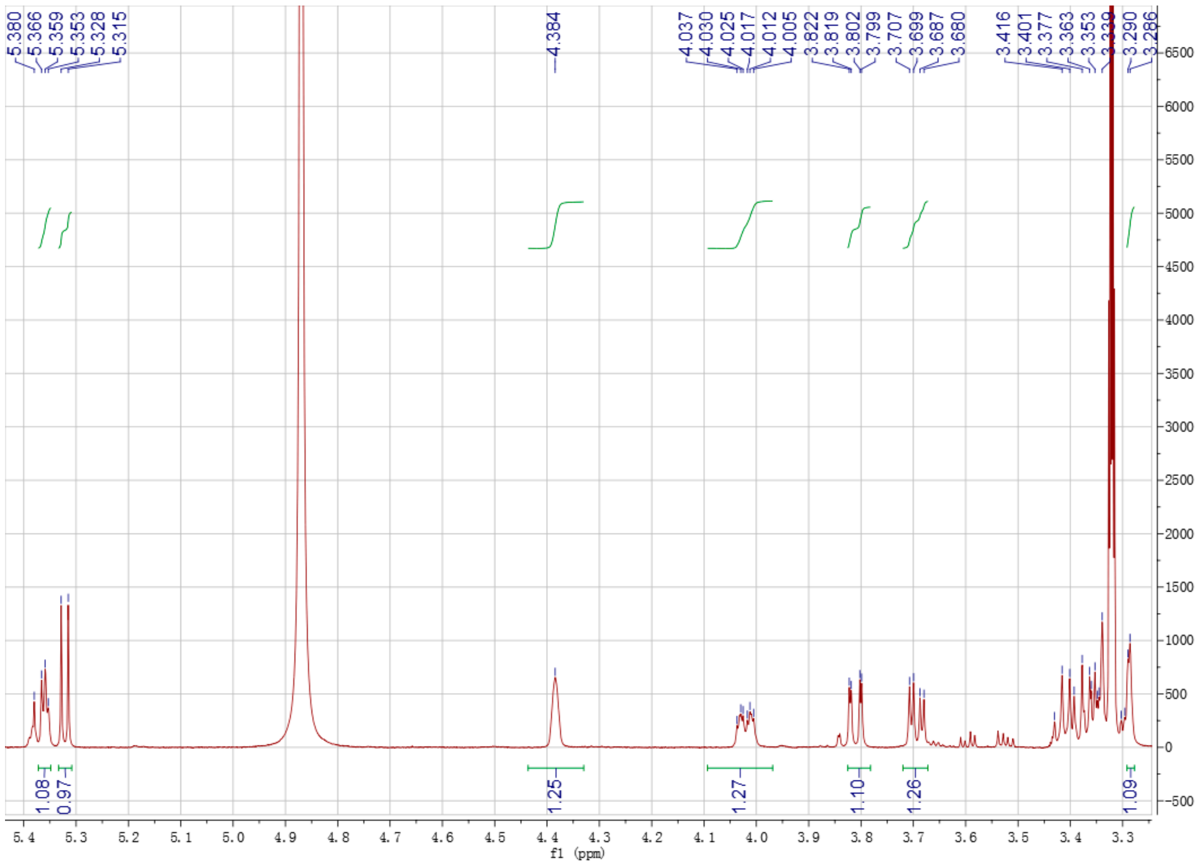

**Figure S4-4.** The Part 2 of the $^{1}$H-NMR Spectrum of Compound **4**.

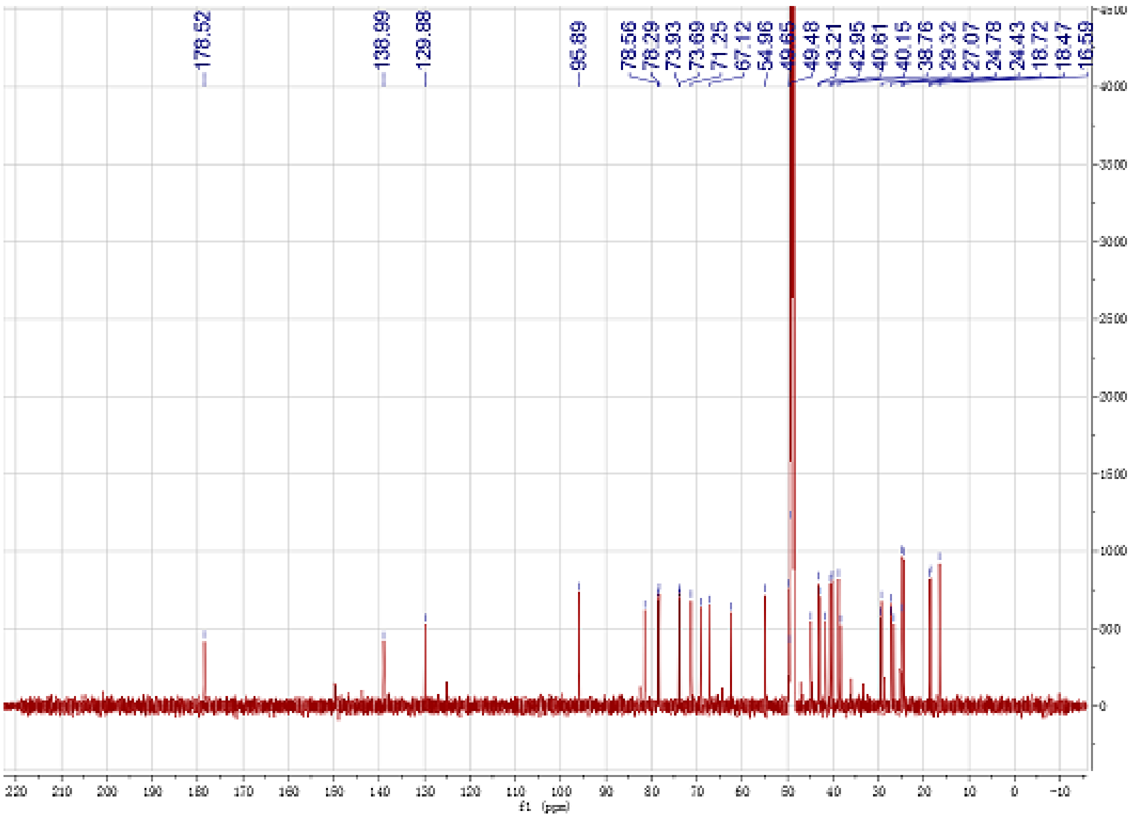

**Figure S5-5.** The $^{13}$C-NMR Spectrum of Compound **4**.

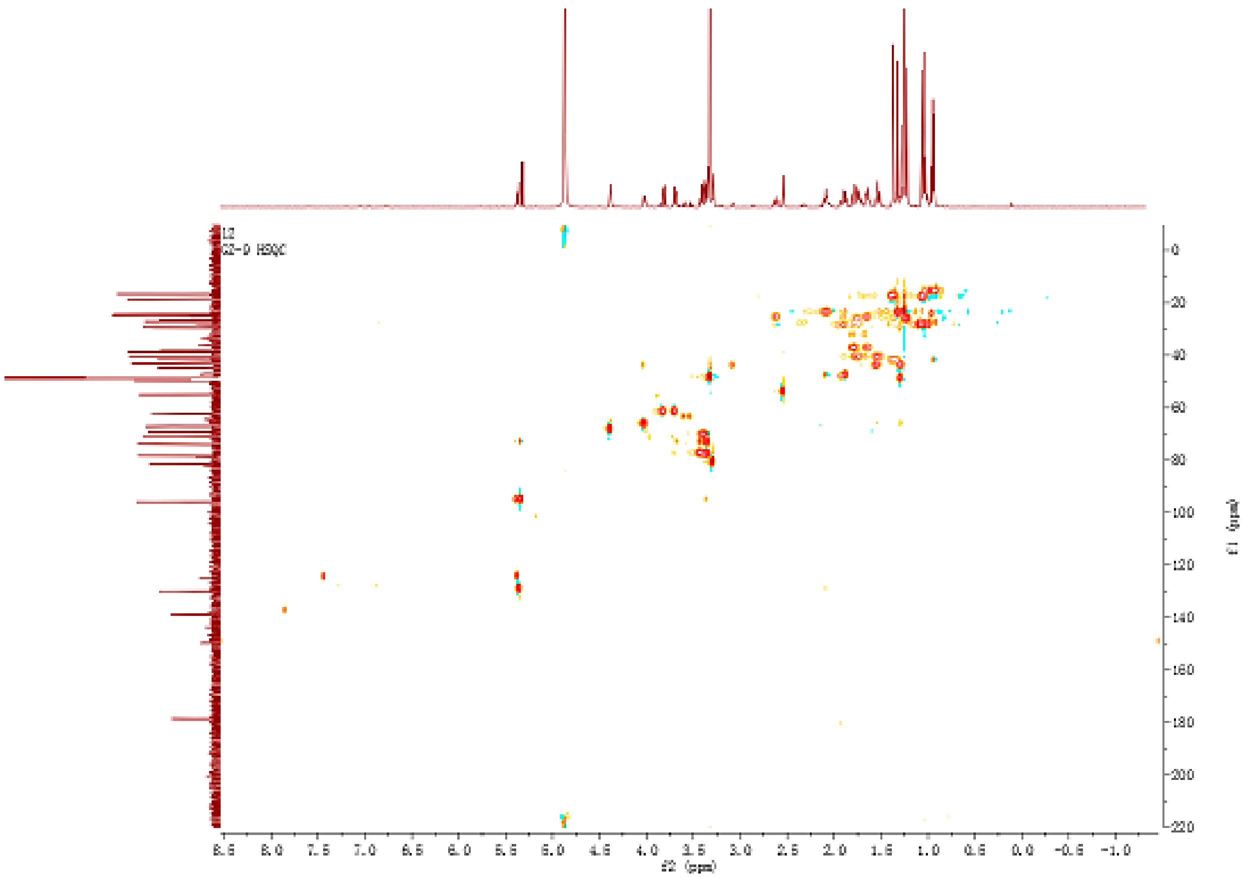


**Figure S4-6.** The Whole HSQC Spectrum of Compound **4**.

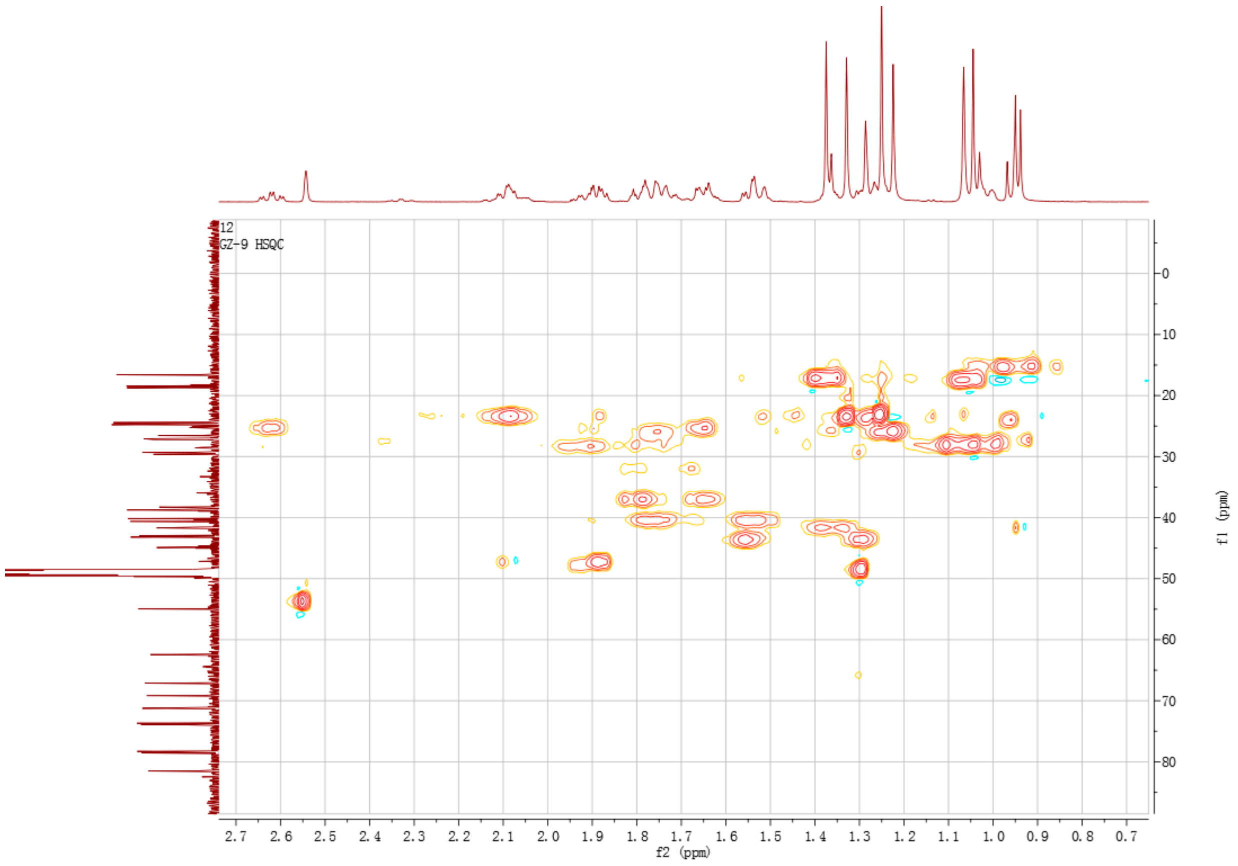


**Figure S4-7.** The Part 1 of the HSQC Spectrum of Compound **4**.

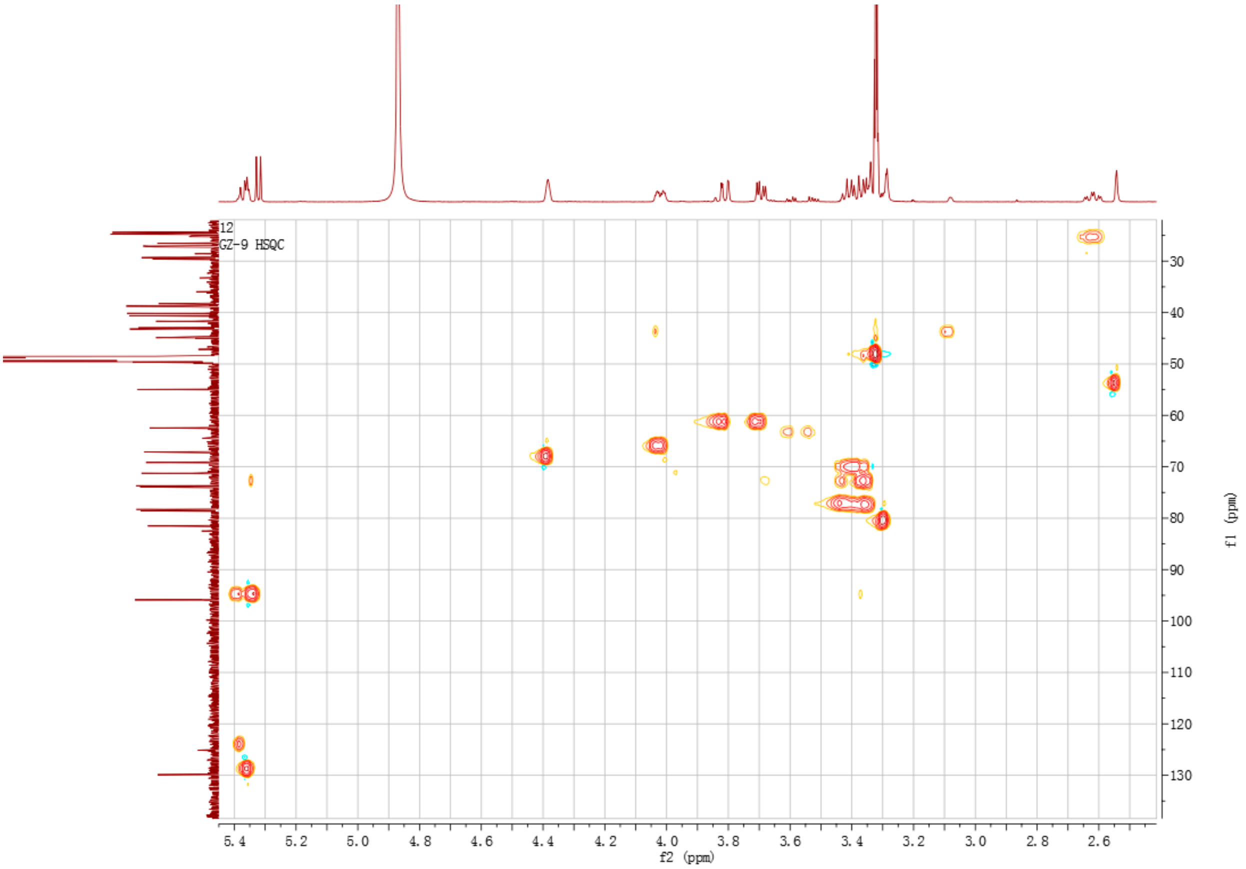


**Figure S4-8.** The Part 2 of the HSQC Spectrum of Compound **4**.

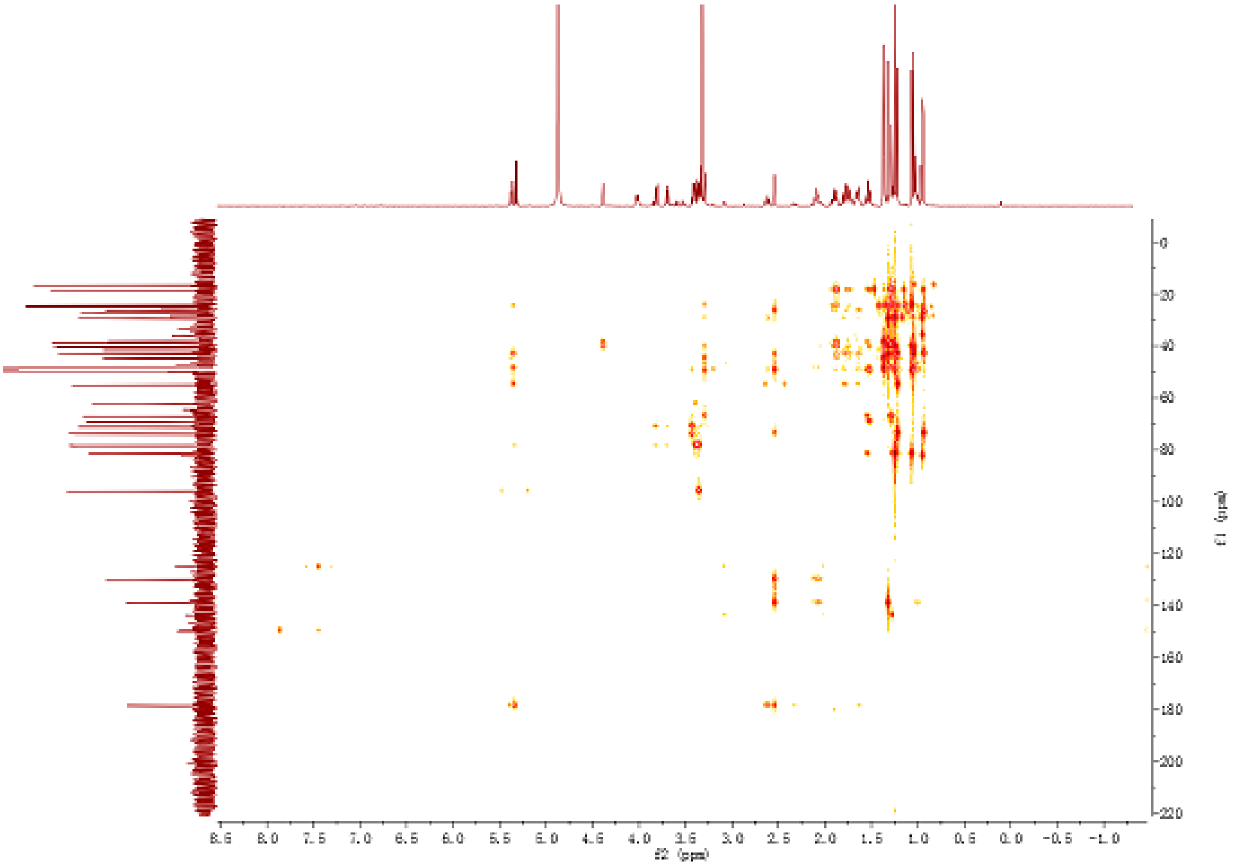


**Figure S4-9.** The Whole HMBC Spectrum of Compound **4**.

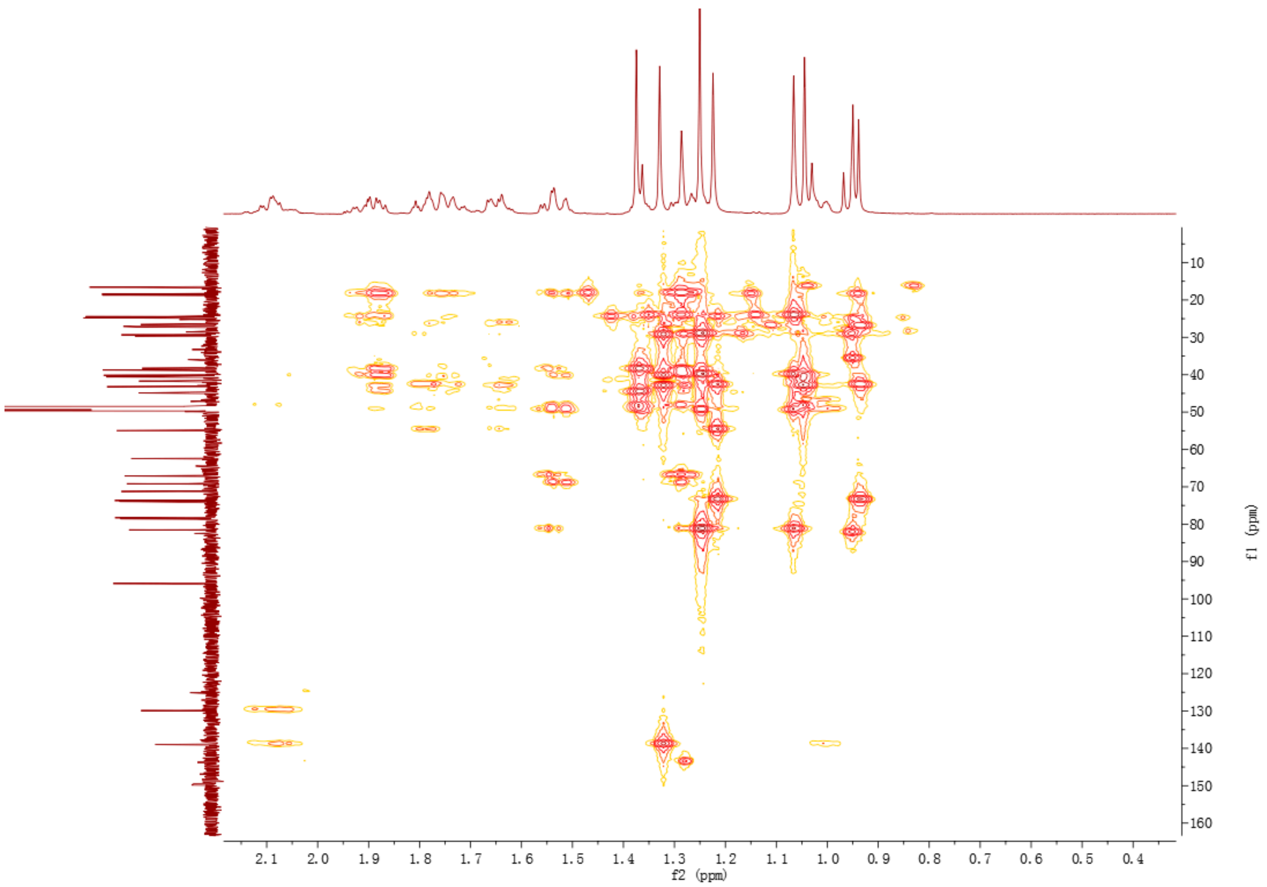


**Figure S4-10.** The Part 1 of the HMBC Spectrum of Compound **4**.

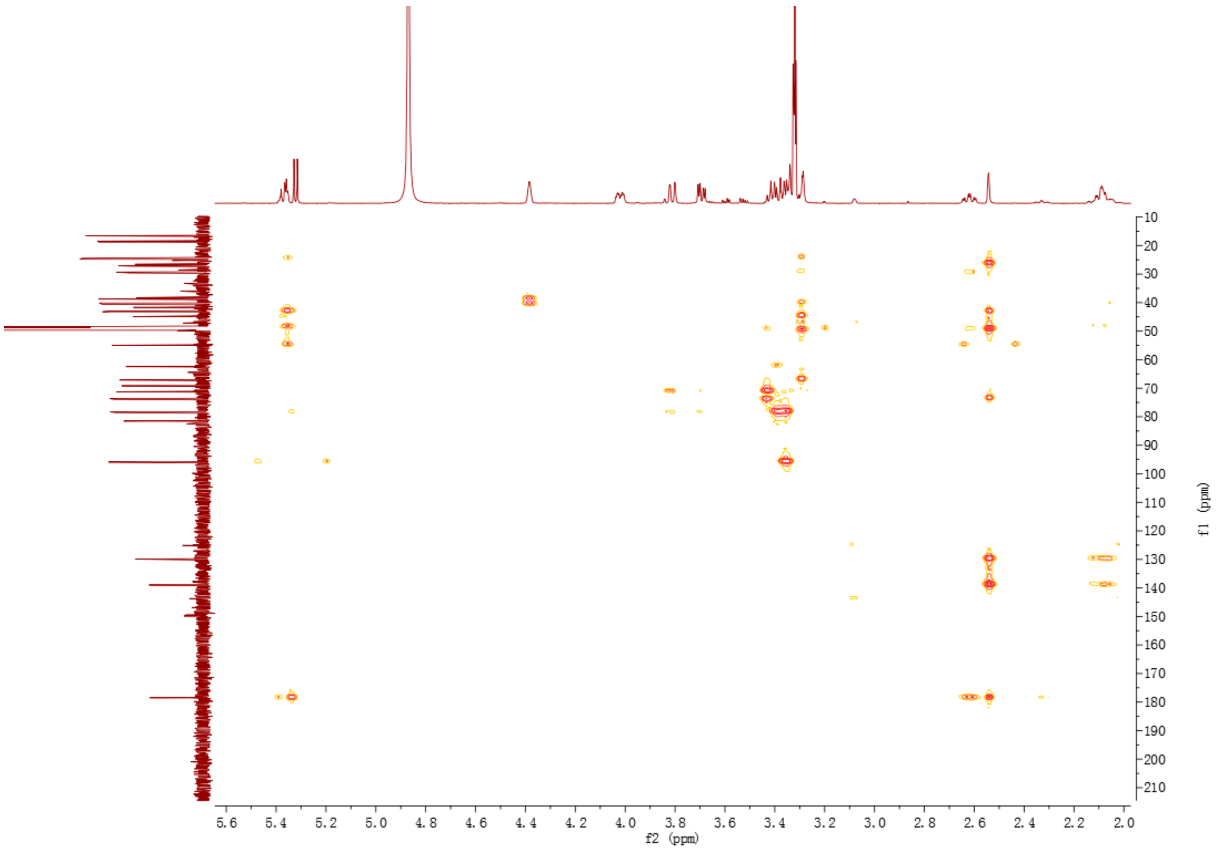


**Figure S4-11.** The Part 2 of the HMBC Spectrum of Compound **4**.

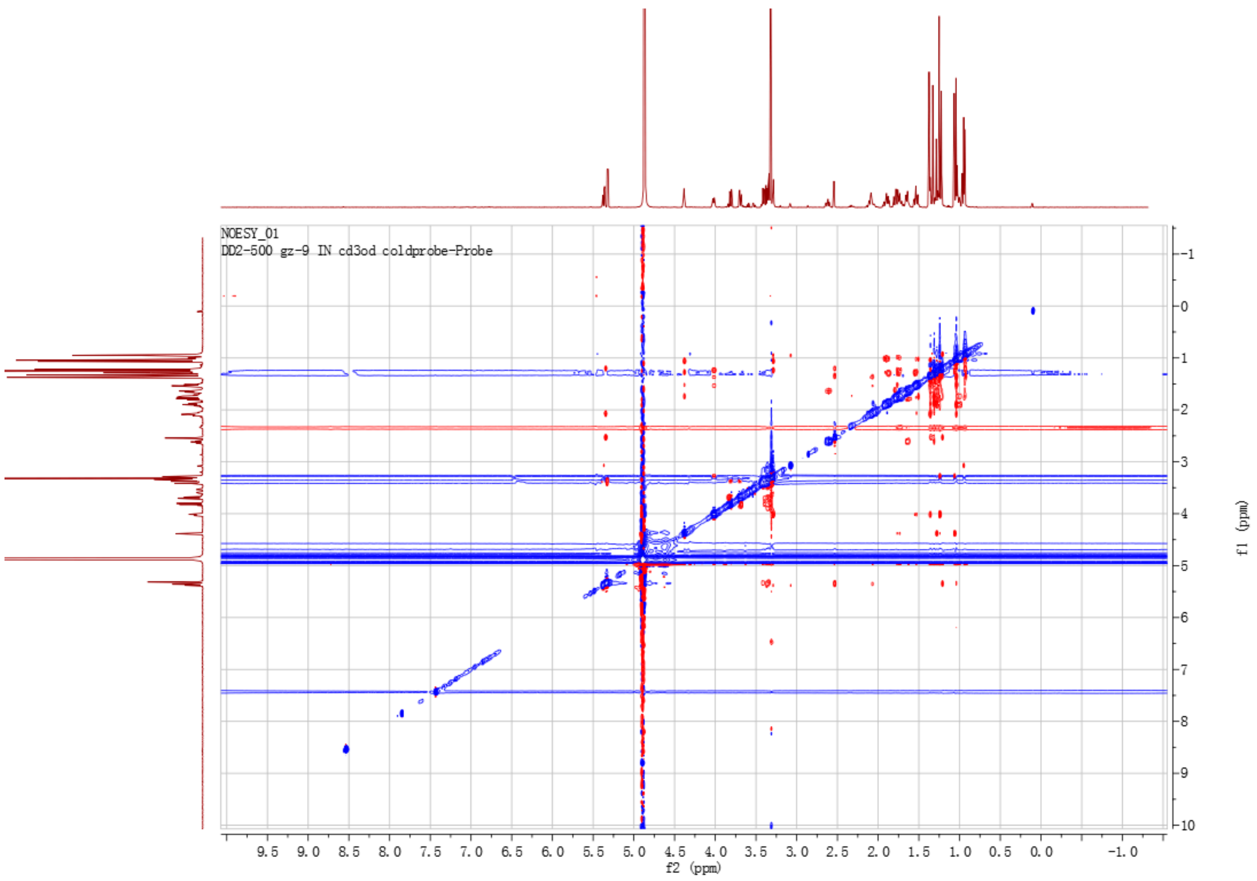


**Figure S4-12.** The Whole NOESY Spectrum of Compound **4**.

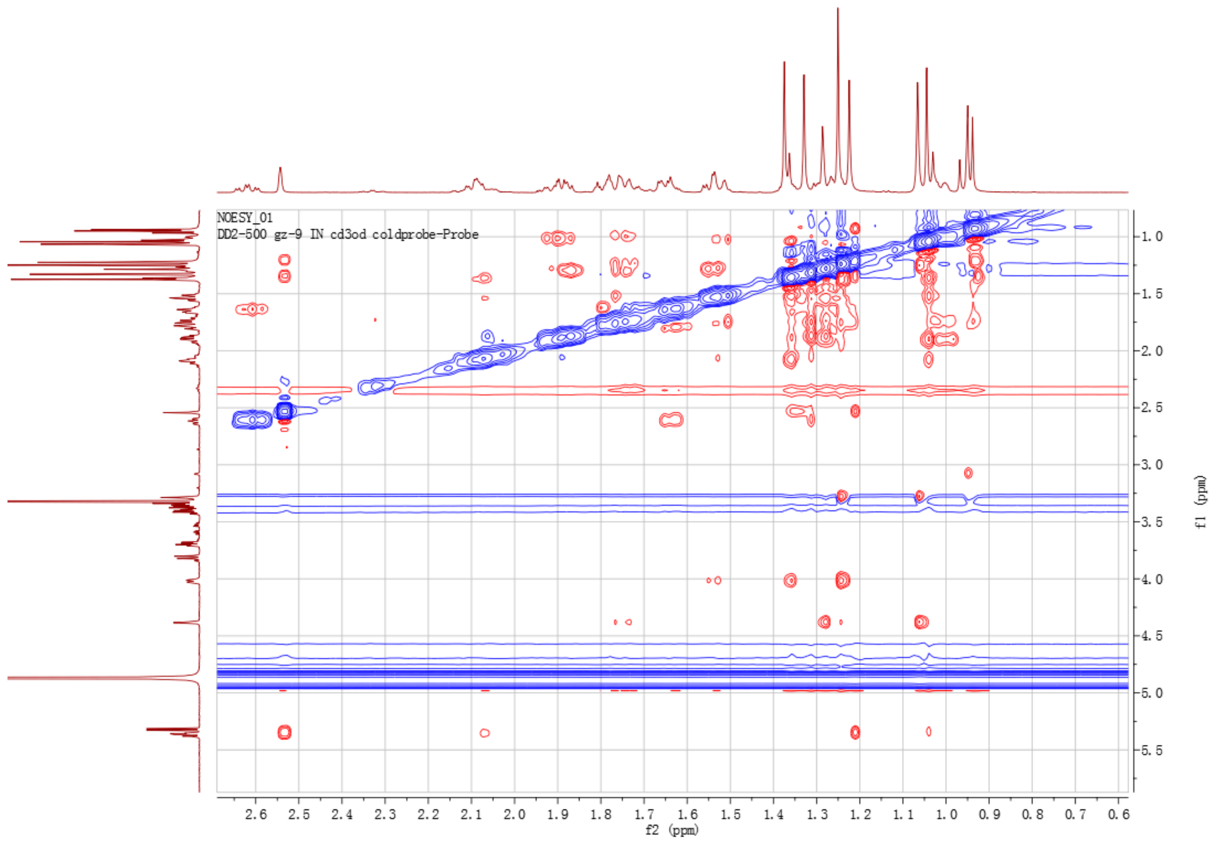


**Figure S4-13.** The Part 1 of the NOESY Spectrum of Compound **4**.

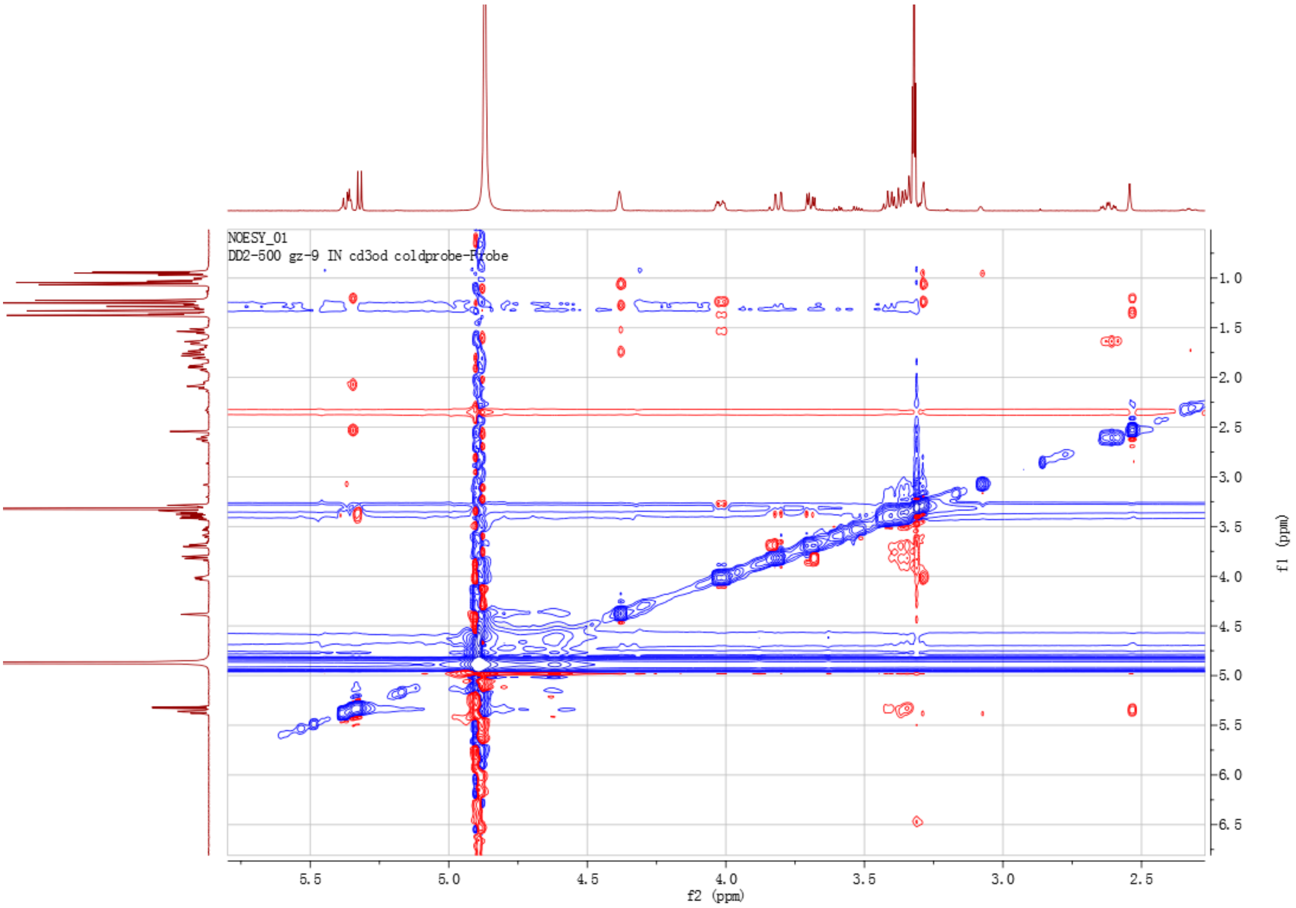


**Figure S4-14.** The Part 2 of the NOESY Spectrum of Compound **4**.
